# Purification of an insect juvenile hormone receptor complex enables insights into its post-translational phosphorylation

**DOI:** 10.1016/j.jbc.2021.101387

**Published:** 2021-11-07

**Authors:** Marek Jindra, William J. McKinstry, Thomas Nebl, Lenka Bittova, Bin Ren, Jan Shaw, Tram Phan, Louis Lu, Jason K.K. Low, Joel P. Mackay, Lindsay G. Sparrow, George O. Lovrecz, Ronald J. Hill

**Affiliations:** 1 Biology Center, Czech Academy of Sciences, Institute of Entomology, Ceske Budejovice, Czech Republic; 2 CSIRO Manufacturing, CSIRO, Parkville, Victoria, Australia; 3 CSIRO Health and Biosecurity, CSIRO, North Ryde, New South Wales, Australia; 4 School of Life and Environmental Sciences, University of Sydney, Sydney, New South Wales, Australia

**Keywords:** juvenile hormone, insect, hormone receptor, basic helix–loop–helix/transcription factor, ligand-binding protein, protein purification, protein phosphorylation, nuclear translocation, methoprene, PAS domain, λPP, lambda protein phosphatase, AaMET, *Aedes aegypti* methoprene-tolerant, AaTAI, *Aedes aegypti* taiman, AHR, aryl hydrocarbon receptor, ARNT, aryl hydrocarbon receptor nuclear translocator, bHLH–PAS, basic helix–loop–helix/Per–Arnt–Sim, BSA, bovine serum albumin, EcR, ecdysone receptor, ESI, electrospray ionization, *ET*, early trypsin, FA, formic acid, FBS, fetal bovine serum, FDR, false discovery rate, GCE, germ cell–expressed, Ha, *Helicoverpa armigera*, HEK293, human embryonic kidney 293 cell line, HSP, heat shock protein, IMAC, immobilized metal affinity chromatography, JH, juvenile hormone, JHR, juvenile hormone receptor, JHRE, juvenile hormone response element, *Kr-h1*, *Krüppel-homolog 1*, MET, methoprene-tolerant, MFBS1, MET–FISC binding site 1, NCBI, National Center for Biotechnology Information, NLS, nuclear localization signal, SEC–MALLS, size-exclusion chromatography in combination with multiangle laser-light scattering, *Sf9*, *Spodoptera frugiperda* 9 cell line, SFM, serum-free media, TAI, Taiman, Tc, *Tribolium castaneum*, TCEP, Tris (2-carboxyethyl) phosphine, TcTAI, *Tribolium castaneum* taiman, TSAP, thermosensitive alkaline phosphatase, USP, ultraspiracle

## Abstract

Juvenile hormone (JH) plays vital roles in insect reproduction, development, and in many aspects of physiology. JH primarily acts at the gene-regulatory level through interaction with an intracellular receptor (JH receptor [JHR]), a ligand-activated complex of transcription factors consisting of the JH-binding protein methoprene-tolerant (MET) and its partner taiman (TAI). Initial studies indicated significance of post-transcriptional phosphorylation, subunit assembly, and nucleocytoplasmic transport of JHR in JH signaling. However, our knowledge of JHR regulation at the protein level remains rudimentary, partly because of the difficulty of obtaining purified and functional JHR proteins. Here, we present a method for high-yield expression and purification of JHR complexes from two insect species, the beetle *T*. *castaneum* and the mosquito *Aedes aegypti*. Recombinant JHR subunits from each species were coexpressed in an insect cell line using a baculovirus system. MET–TAI complexes were purified through affinity chromatography and anion exchange columns to yield proteins capable of binding both the hormonal ligand (JH III) and DNA bearing cognate JH-response elements. We further examined the beetle JHR complex in greater detail. Biochemical analyses and MS confirmed that *T*. *castaneum* JHR was a 1:1 heterodimer consisting of MET and Taiman proteins, stabilized by the JHR agonist ligand methoprene. Phosphoproteomics uncovered multiple phosphorylation sites in the MET protein, some of which were induced by methoprene treatment. Finally, we report a functional bipartite nuclear localization signal, straddled by phosphorylated residues, within the disordered C-terminal region of MET. Our present characterization of the recombinant JHR is an initial step toward understanding JHR structure and function.

Juvenile hormone (JH) and 20-hydroxyecdysone are the two major hormones controlling insect molting and metamorphosis (1, 2). These hormones also regulate a range of developmental and physiological processes, including reproduction, behavior, morphological polyphenism, metabolism, and immunity (3, 4, 5, 6, 7). The nuclear ecdysone receptor (EcR), initially cloned from *Drosophila melanogaster* (8), has been shown to dimerize with another nuclear receptor ultraspiracle (USP) to form an active 20-hydroxyecdysone receptor (9, 10). Purification of functional recombinant EcR–USP protein complexes from pest insects has revealed the structural mode of the receptor–ligand interaction, explained the order selectivity of EcR-targeting insecticides (11), and aided development of insecticide discovery tools (12).

The nature of the intracellular receptor for JH has proven more problematic with a number of candidates being considered (13). *D. melanogaster* USP was observed to bind, initially JH III, and subsequently other sesquiterpenoid methyl farnesoids (14), suggesting a mechanism whereby the JH and ecdysone signaling pathways might converge through the EcR–USP complex. However, a JH receptor (JHR) role for USP has proven controversial on several grounds, including the absence of a JH binding pocket in the USP subunits of established ecdysteroid–EcR–USP X-ray structures (15, 16). The observation of a patent ligand-binding pocket in the structure of a hitherto unobserved apo form of an EcR–USP heterodimer (17) may reopen this discussion.

Wilson and Fabian (18) discovered in *D. melanogaster* a gene, termed *Methoprene-tolerant* (*Met*), which confers sensitivity to the insecticide methoprene, a JH mimic. *Met* and its *D. melanogaster* paralog *germ cell–expressed* (*gce*) encode transcription factors of the basic helix–loop–helix/Per–aryl hydrocarbon receptor nuclear translocator (Arnt)–Sim (bHLH–PAS) family (19, 20). The MET and GCE proteins from *D. melanogaster* and their orthologs from diverse insect species display properties expected for a JHR (21), including binding of JH III with nanomolar affinities (22, 23, 24, 25). This hormone binding, mediated by a pocket within the PAS-B domain of MET/GCE (23, 24, 25), is highly stereoselective (26). Genetic rescue of nonconditional lethality in *D. melanogaster* mutants lacking both MET and GCE (27) has revealed that the JH-binding capacity of either protein is required for the flies to complete development (25). As initially demonstrated in larvae of the beetle *T*. *castaneum* (28) and later in diverse other insects (29, 30, 31), deficiency of *Met* function leads to precocious metamorphosis, a phenotype consistent with a deficiency of JH itself. Importantly, MET mediates the effects of JH by directly inducing transcription of genes such as *Krüppel-homolog 1* (*Kr-h1*) whose product(s) repress metamorphosis (29, 32, 33, 34, 35, 36, 37).

To activate transcription, bHLH–PAS factors typically form heterodimers with other members of this protein family (38). Li *et al.* (39) employed a yeast two-hybrid system to detect in the mosquito, *A. aegypti*, a bHLH–PAS protein that binds MET in the presence of methoprene. A partner protein has also been identified for MET of *T. castaneum* (40) and shown to interact with it in a manner depending on JH or methoprene binding to MET (23). Both the mosquito and beetle MET partnering proteins are counterparts of the mammalian steroid receptor coactivator 1, whose first insect homolog was identified in *D. melanogaster* as the product of a gene *taiman* (TAI) (41). We will refer to this protein as TAI. MET–TAI complexes, presumed heterodimers, have been shown to associate with JH response elements (JHREs) in the enhancers of *Kr-h1*, *early trypsin* (*ET*), and other JH-responsive genes of *A. aegypti* (24, 33, 39, 42, 43). RNAi-mediated knockdown in the cockroach *Blattella germanica* has revealed a role for TAI in suppressing metamorphosis (44). As expected for subunits of a functional JHR, coexpression of MET and TAI has been shown to confer JH responsiveness to otherwise JH nonresponsive mammalian cells (33, 45).

In addition to its direct gene-regulatory action, JH exerts effects initiated at the cell membrane. While investigating protein synthesis in the *D. melanogaster* male accessory glands, Yamamoto *et al.* (46) described a pathway involving Ca^2+^ signaling and a protein kinase C. Synthesis of accessory gland proteins was reduced in *Met* mutant males (47), suggesting that MET was also involved. Detailed investigations by Liu *et al.* (48) in *A. aegypti* have linked JH action through an as yet unknown cell-membrane receptor and Ca^2+^/second messenger signaling to phosphorylation of MET and TAI, which in turn led to an increased transcriptional activity of the intracellular JHR. Several phosphorylation sites in *Aedes aegypti* methoprene-tolerant (AaMET) have recently been reported (49). Clearly, understanding of post-translational regulation of the intracellular JHR is of considerable interest. However, purification of a native and functional JHR complex from insect cells has not previously been accomplished.

In this communication, we present high-yield insect cell–based expression and purification of recombinant JHR MET–TAI complexes from *T. castaneum* and *A. aegypti* that are active in both hormone and target JHRE DNA binding. We establish the presumed, but hitherto unproven, 1:1 ratio between the receptor protein subunits and identify a conserved signal of nuclear import of MET. Our primary identification of amino acid residues phosphorylated in the active MET–TAI complex paves the way to understanding the role of post-translational regulation in JH signaling.

## Results

### Expression and purification of JHR proteins from *T. castaneum* and *A. aegypti*

**Figure 1:**
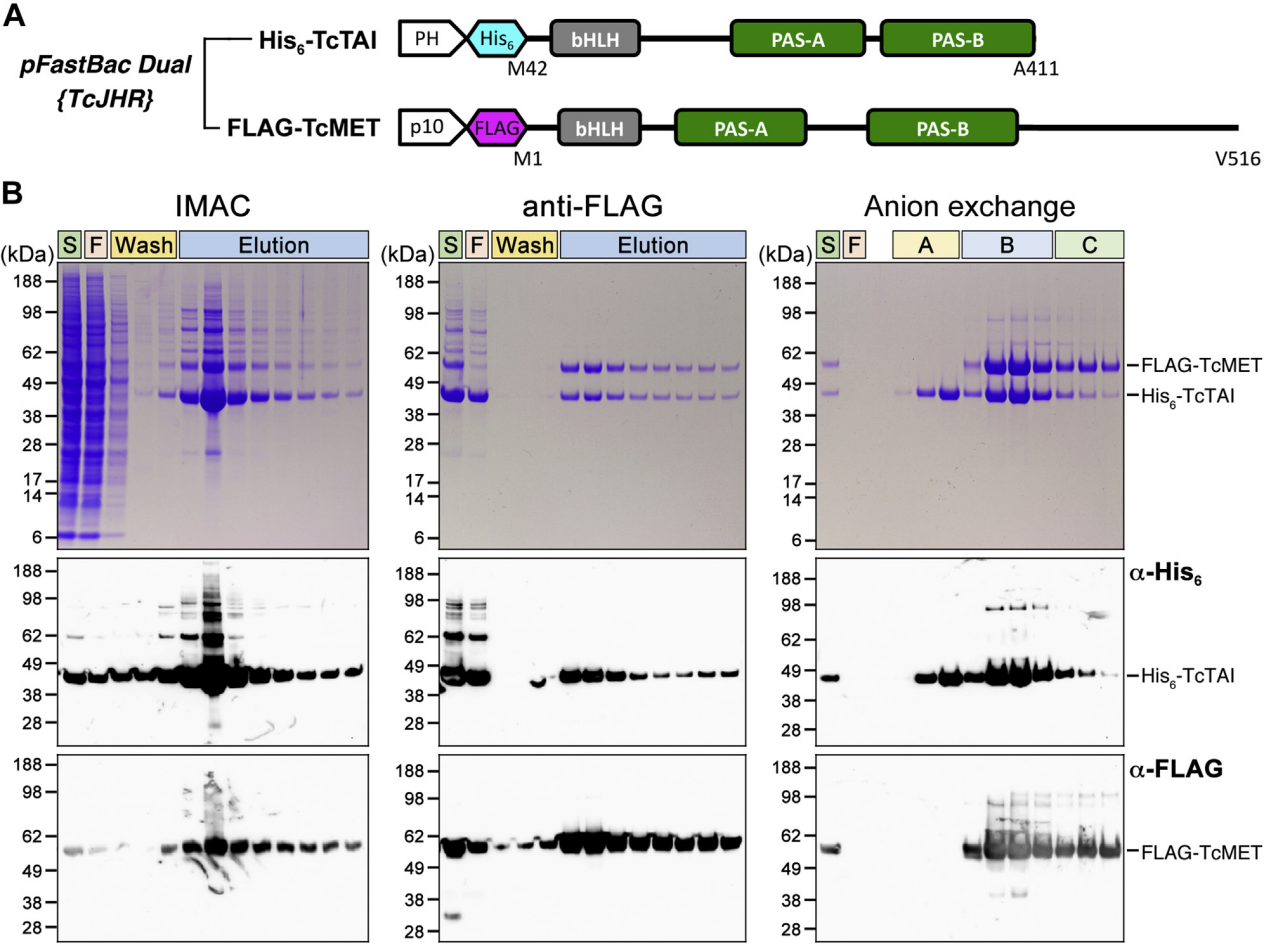
**Expression and purification of the recombinant JHR proteins of *T*.*castaneum*.***A*, the interacting components of the *T. castaneum* JH receptor complex, the truncated TcTAI and the full-length *T. castaneum* methoprene-tolerant proteins, were coexpressed in *Sf9* cells from the baculoviral *pFastBac Dual* construct under the polyhedrin (PH) and p10 promoters, respectively. N-terminal affinity tags and the major functional domains are depicted. *B*, the *T. castaneum* JH receptor protein complex was purified from lysates of the baculovirus-infected *Sf9* cells in two affinity chromatography steps using immobilized Ni^2+^ (IMAC) and anti-FLAG antibody, respectively, followed by anion exchange chromatography, all in the presence of 10 μM methoprene. Fractions under “B” containing both *T. castaneum* methoprene-tolerant and TcTAI were pooled for further work. *Top panels* show Coomassie-stained SDS-PAGE; below are immunoblots of the same samples developed with the indicated antitag antibodies. F, flow-through; IMAC, immobilized metal affinity chromatography; JHR, juvenile hormone receptor; S, starting lysate; TcTAI, *T*. *castaneum* taiman.

The affinity-tagged *T. castaneum* JH receptor proteins, FLAG-*T. castaneum* methoprene-tolerant and His_6_-*Tribolium castaneum* taiman (TcTAI), were coexpressed in *Sf9* insect cells infected with a baculovirus expression construct depicted in Figure 1*A*. While *T. castaneum* methoprene-tolerant was expressed in its entirety, TcTAI sequence was limited to include the bHLH and the two tandem PAS domains, that is, regions that are required and sufficient for TcTAI to engage in the JH-stimulated interaction with *T. castaneum* methoprene-tolerant (23). The tagged *T. castaneum* JH receptor proteins were purified through successive affinity chromatography steps using immobilized Ni^2+^ (immobilized metal affinity chromatography [IMAC]) and anti-FLAG antibody, respectively (Fig. 1*B*). A final anion exchange chromatography step provided further purification and concentration. All the purification steps were performed in the presence of 10 μM methoprene, a JH analog and an agonist ligand of *T. castaneum* methoprene-tolerant (23). The purified *T. castaneum* JH receptor is a complex of the FLAG-*T. castaneum* methoprene-tolerant and His_6_-TcTAI subunits detected by anti-His_6_ and anti-FLAG antibodies and exhibiting apparent molecular weights consistent with those predicted from the FLAG-*T. castaneum* methoprene-tolerant and His_6_-TcTAI sequences (59.8 and 42.7 kDa, respectively). The yield of the *T. castaneum* JH receptor protein complex ranged from 1 to 1.5 mg per 10 g of packed cells, that is, 2 to 3 mg per liter of culture.

A baculovirus construct was also designed for coexpression of tagged *A. aegypti* JHR proteins His_6_-AaMET and FLAG-*Aedes aegypti* taiman (AaTAI) (Fig. S1*A*) in *Sf9* cells. AaMET was N-terminally truncated to remove the first 113 amino acids, whereas AaTAI only contained the bHLH, and both of the PAS domains involved in the JH-dependent interaction with AaMET (39). The AaJHR protein purified as a complex of His_6_-AaMET and FLAG-AaTAI subunits of apparent molecular weights consistent with those calculated from their sequences in the expression construct (95.1 and 46.8 kDa, respectively) (Fig. S1*B*). Yields of the purified mosquito protein (0.25–0.5 mg per 10 g of packed cells, 0.5–1 mg per liter of culture) were lower relative to the yields of *T. castaneum* JH receptor, largely as the result of greater susceptibility to proteolysis (even in the presence of inhibitors) and adsorption to surfaces/reduced solubility.

### The purified *T. castaneum* JH receptor complex is a MET–TAI heterodimer stabilized by methoprene

**Figure 2:**
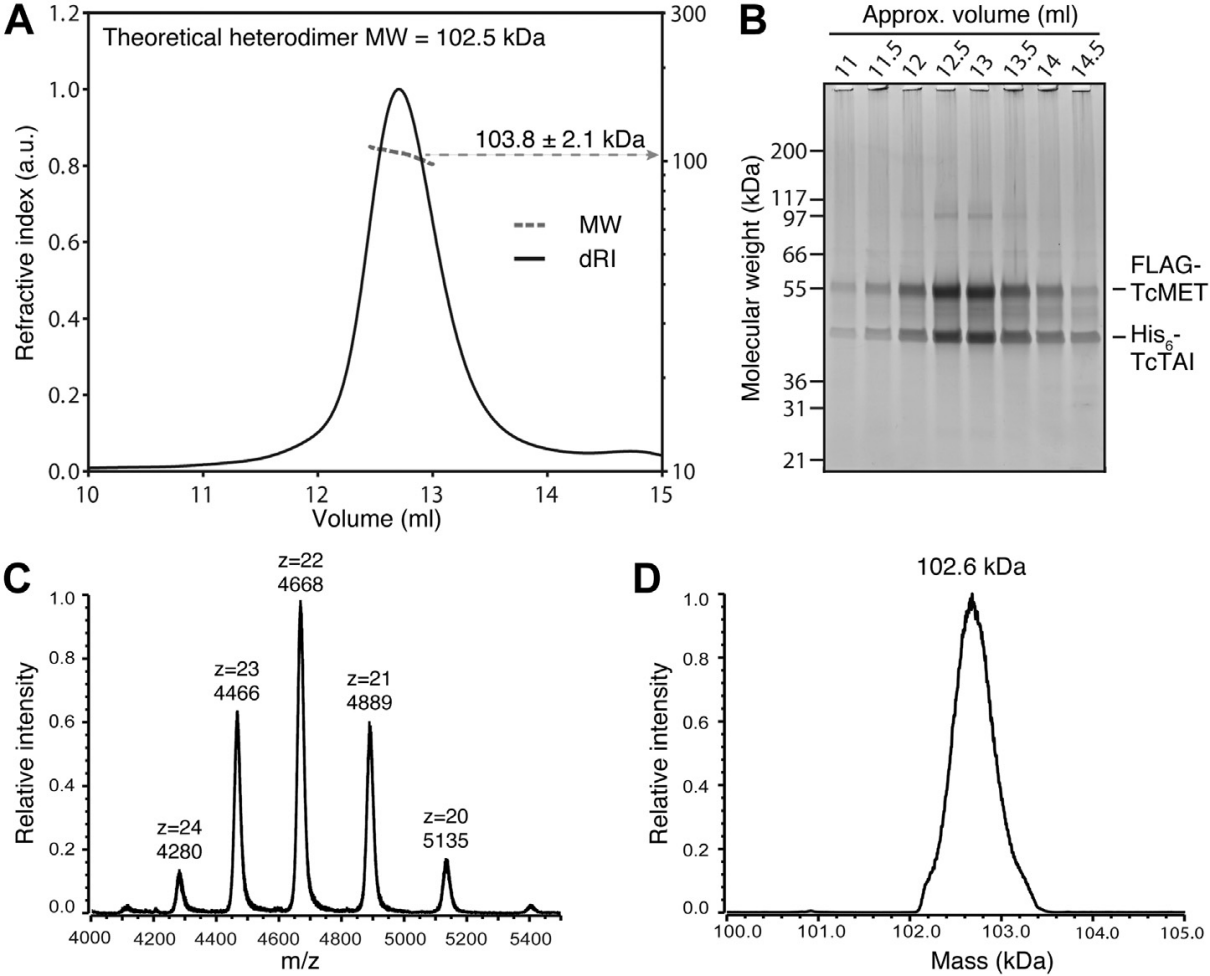
***T. castaneum*****JH receptoris predominantly a heterodimer of*****T. castaneum*****methoprene-tolerantand TcTAI in solution.***A*, SEC–MALLS analysis of the *T. castaneum* JH receptor complex (loading concentration of ∼5 mg/ml) showing protein concentration in refractive index units (dRI, *black line*) and the calculated weight-average molecular weight (MW, *broken gray line*). The observed molecular weight was ∼103.8 ± 2.1 kDa. *B*, electrophoretic analysis of fractions of the main peak from the SEC–MALLS analysis. *T. castaneum* methoprene-tolerant has a theoretical mass of 59.7 kDa, whereas TcTAI has a theoretical mass of 42.7 kDa. *C*, the native MS spectrum of purified recombinant *T. castaneum* JH receptor complexes shows 20^+^ to 24^+^ charge states (4000–5500 *m/z* range). *D*, a zoom of the deconvoluted native MS spectrum of the *T. castaneum* JH receptor complex provides a neutral mass estimate of 102.6 ± 0.5 kDa, closely matching the theoretical mass of a *T. castaneum* methoprene-tolerant–TcTAI heterodimer. SEC–MALLS, size-exclusion chromatography in combination with multiangle laser-light scattering; TcTAI, *T*. *castaneum* taiman.

Size-exclusion chromatography in combination with multiangle laser-light scattering (SEC–MALLS) indicated that FLAG-*T. castaneum* methoprene-tolerant–His_6_-TcTAI was predominantly a heterodimer in solution with an observed experimental molecular weight of 103.8 ± 2.1 kDa (Fig. 2*A*; theoretical molecular weights of the FLAG-*T. castaneum* methoprene-tolerant–His_6_-TcTAI heterodimer, FLAG-*T. castaneum* methoprene-tolerant homodimer, and His_6_-TcTAI homodimer are 102.5, 119.5, and 85.5 kDa, respectively). Post SEC–MALLS, proteins in the fractions including and surrounding the main peak were separated *via* SDS-PAGE (Fig. 2*B*), and it was observed that both *T. castaneum* methoprene-tolerant and TcTAI are present in approximately equal stoichiometric quantities across the peak, consistent with the two proteins interacting as a heterodimeric complex.

The theoretical heterodimer mass was also validated by nano electrospray-time of flight MS under native conditions (native MS), with 20^+^ to 24^+^ charge states of the native complex detected across the 4000 to 5500 Da mass/charge range (Fig. 2*C*). A zoom of the neutral mass spectrum confirmed an average mass of 102.6 ± 0.5 kDa of the methoprene-bound *T. castaneum* JH receptor complex (Fig. 2*D*).

We observed that dialysis against buffer lacking methoprene led to instability of the complex with the formation of higher molecular weight material within 24 h as monitored by SEC–MALLS (Fig. S2*A*). Moreover, it was also observed that solutions of FLAG-*T. castaneum* methoprene-tolerant–His_6_-TcTAI complex, even in the presence of methoprene, underwent aggregation over a period of days as assessed by dynamic light scattering (Fig. S2*B*). This was not prevented by the presence of thiol reagents, 10 mM DTT, 10 mM 2-mercaptoethanol, or 20 mM Tris (2-carboxyethyl) phosphine (TCEP). Together, the present results indicate that purified and recombinant *T. castaneum* JH receptor preparations comprise an initially relatively stable *T. castaneum* methoprene-tolerant–TcTAI heterodimer species with a tendency to aggregate in solution on standing for periods of more than a day.

### JHRE-binding capacity of the purified JHR complexes

By dimerizing, bHLH-PAS partners form bipartite, basic DNA-binding domains that are necessary for these transcription factors to recognize and bind DNA in order to initiate transcription of specific genes (38). To test whether the purified *T. castaneum* JH receptor and AaJHR complexes retained DNA-binding activity, the proteins were allowed to interact with radiolabeled DNA probes representing three types of JHREs. Two of them derive from JH-response genes *Kr-h1* of *T. castaneum* (50) and *ET* of *A. aegypti* (39) and are designated *k*-JHRE and *ET*-JHRE, respectively. The third probe carries a consensus binding site for the AaMET–AaTAI complex that was derived from a random oligonucleotide library through repeated cycles of JHR binding, selection, and amplification, and named MET–FISC binding site 1 (MFBS1) (24).

**Figure 3:**
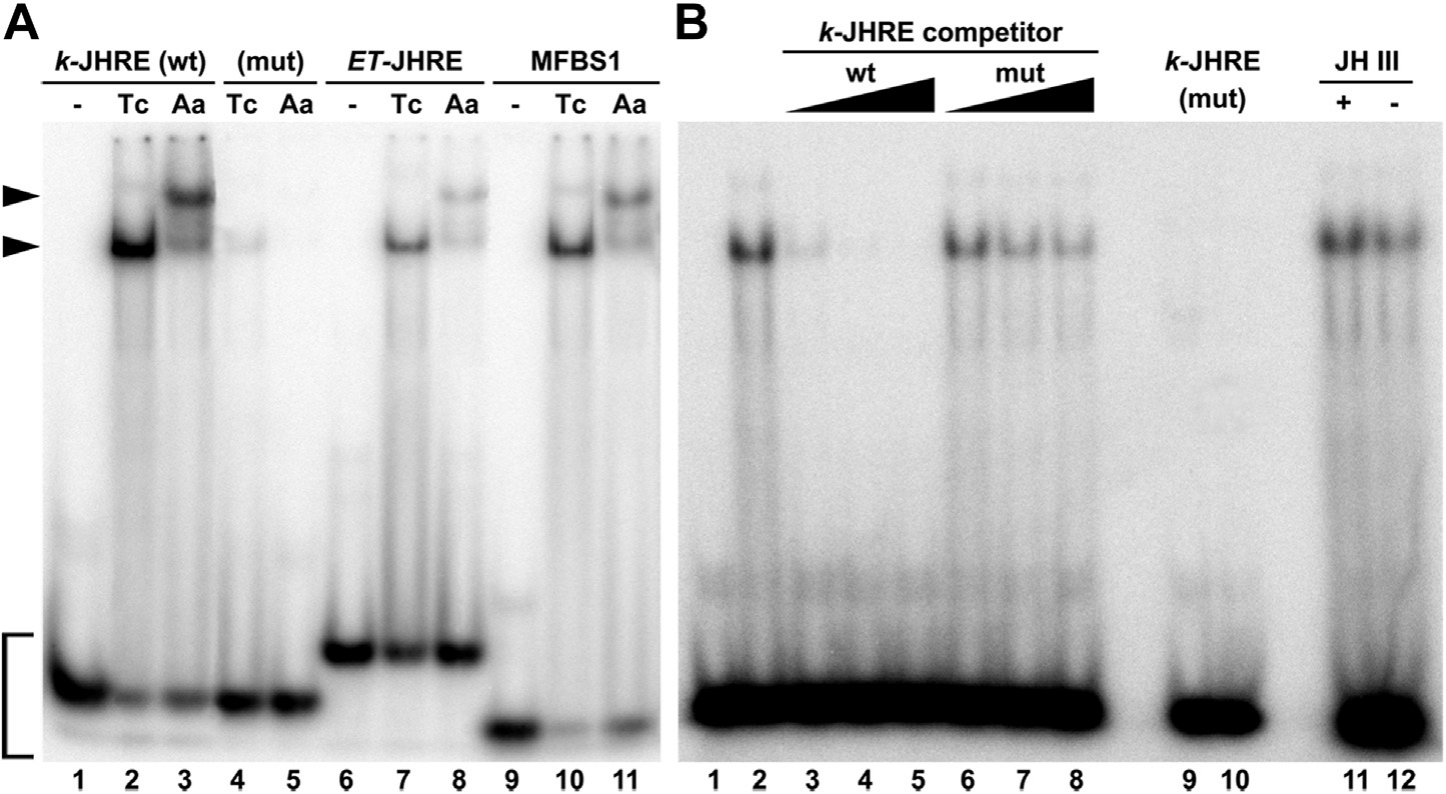
**Recombinant*****T. castaneum*****JH receptorand AaJHR protein complexes bind cognate DNA elements.***A*, the purified *T. castaneum* JH receptor (Tc) and AaJHR (Aa) proteins were incubated with the radiolabeled DNA probes in the presence of 10 μM JH III; lanes 1, 6, and 9 contained no protein input. The receptor complexes from both species bound to the WT (wt) sequence of *k*-JHRE (lanes 2 and 3), but they bound poorly to the mutated (mut) version of *k*-JHRE (lanes 4 and 5). The retarded protein–DNA complexes (*arrowheads*) were detected upon electrophoresis by autoradiography. *B*, the *T. castaneum* JH receptor protein complex was incubated with the *k*-JHRE DNA probe in the presence of 100-, 500-, and 1000-fold excess of either WT (wt) or mutated (mut) versions of the unlabeled *k*-JHRE DNA competitor. Lane 1 contained no protein input; lanes 9 and 10 contained radiolabeled mutant version of *k*-JHRE. JH III (10 μM) was added to all reactions except that in lane 12. The *bracket* indicates the unbound radiolabeled DNA probes. AaJHR, *Aedes aegypti* juvenile hormone receptor; JHRE, juvenile hormone response element.

The EMSA data in Figure 3*A* show that both the *T. castaneum* methoprene-tolerant–TcTAI and AaMET–AaTAI protein complexes bound the WT *T. castaneum* *k*-JHRE sequence but not its mutated version (lanes 1–5). The JHR protein complexes from both species also bound the *ET*-JHRE and the consensus binding site MFBS1 (Fig. 3*A*; lanes 6–11). To confirm the specificity of the interaction, we show that excess of the unlabeled *k*-JHRE DNA but not its mutated version competed with the [^32^P]-labeled *k*-JHRE probe for the *T. castaneum* methoprene-tolerant–TcTAI complex (Fig. 3*B*; lanes 1–8), whereas the radiolabeled mutant *k*-JHRE itself could not bind (lanes 9 and 10). Excess of the unlabeled WT *k*-JHRE but not of the mutant *k*-JHRE also effectively competed with the binding of the AaMET–AaTAI protein complex to the [^32^P]-labeled *k*-JHRE probe (Fig. S3). To test whether JH III was required for *T. castaneum* JH receptor binding to *k*-JHRE, we omitted JH III, which was otherwise added at 10 μM concentration to all EMSA reactions. The persisting protein–DNA complex (Fig. 3*B*; lane 12) suggests that once formed in the presence of methoprene during the expression and purification steps, the *T. castaneum* methoprene-tolerant–TcTAI dimer is sufficiently stable to display *k*-JHRE binding in the EMSA without addition of hormone. This result is consistent with previous findings that JH was not necessary for binding of a bacterially expressed *A. aegypti* MET–TAI complex to DNA (24). The apparent slight decrease in the band intensity in lane 12 relative to lanes 2 and 11 (Fig. 3*B*) may however reflect some instability of the *T. castaneum* methoprene-tolerant–TcTAI dimer in the absence of JH III during the assay.

### The purified JHR complexes display JH-binding activity

**Figure 4:**
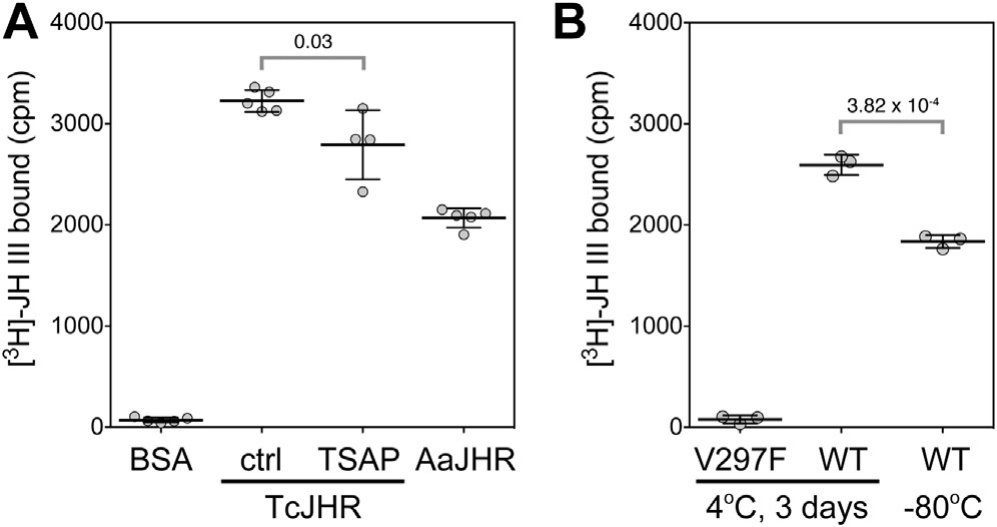
**Recombinant JHR complexes bind JH III.***A*, the purified ***T. castaneum*****JH receptor**, either untreated (ctrl) or dephosphorylated (TSAP), AaJHR, and BSA as an inactive control were assessed for the ability to bind [^3^H]-JH III (assay described in the Experimental procedures section). *B*, the same assay was applied to the *T. castaneum* JH receptor complex, partially purified through the Ni-IMAC chromatography, after storage of the protein in elution buffer (20 mM Tris–HCl [pH 7.8], 150 mM NaCl, 250 mM imidazole), supplemented with 1 μM methoprene and 10% glycerol. The samples were either stored refrigerated at 4 °C for 3 days or thawed 1 month after freezing in liquid nitrogen and storage at −80 °C. A *T. castaneum* JH receptor complex containing *T. castaneum* methoprene-tolerant^V297F^, a mutated variant incapable of binding [^3^H]-JH III (23), was tested in parallel to the *T. castaneum* JH receptor complex containing functional *T. castaneum* methoprene-tolerant (WT). Note that while all protein samples were purified in the presence of methoprene, [^3^H]-JH III binding occurred, likely because the binding affinity of [^3^H]-JH III to *T. castaneum* methoprene-tolerant far exceeds that of methoprene (23). The data are mean values; error bars represent SD; the *p* values are calculated by *t* test. AaJHR, *Aedes aegypti JHR*; BSA, bovine serum albumin; IMAC, immobilized metal affinity chromatography; JHR, juvenile hormone receptor; TSAP, thermosensitive alkaline phosphatase.

**Figure 5:**
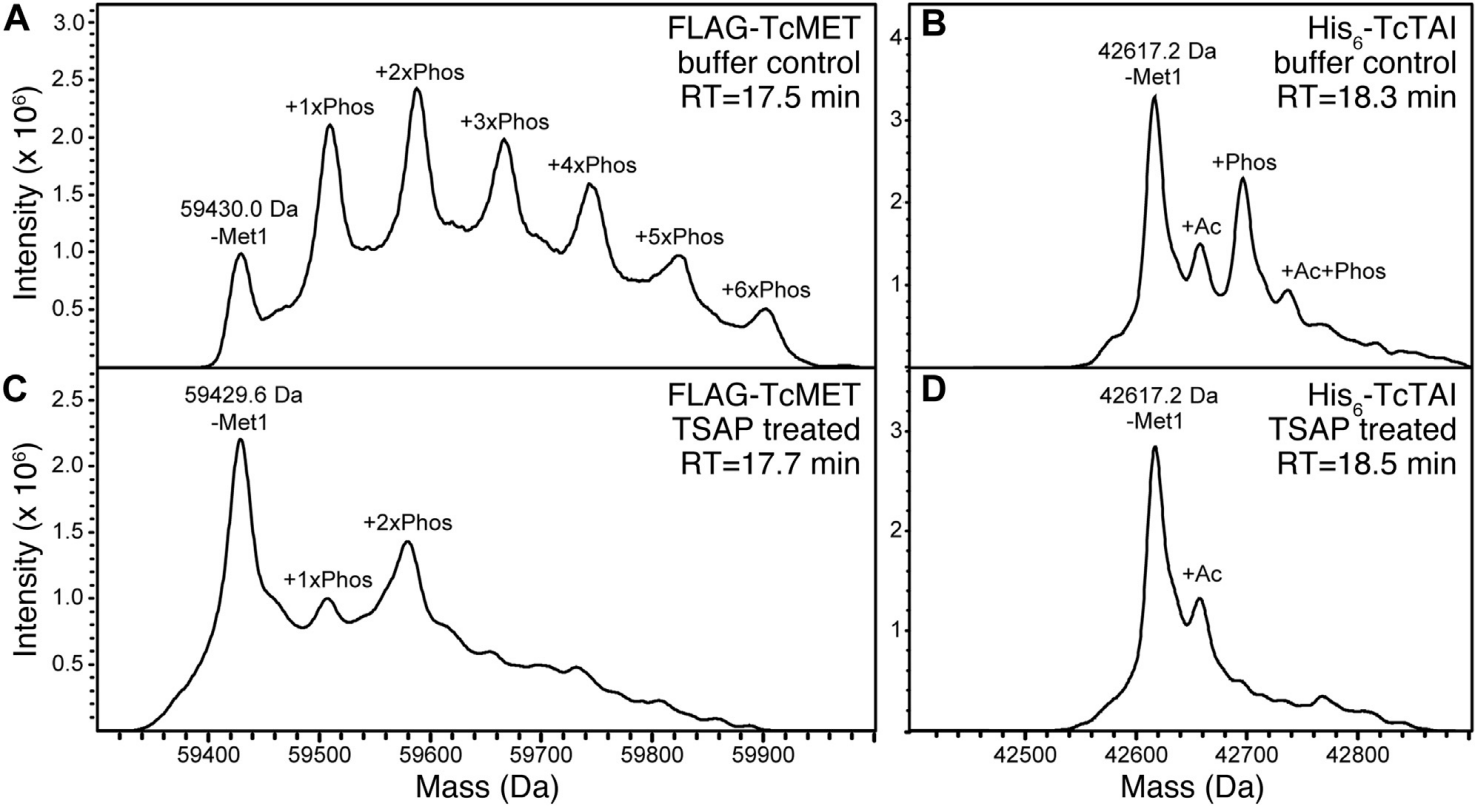
**LC–ESI–MS of recombinant purified*****T. castaneum*****JH receptor complexes under denaturing conditions.***A*, a zoom at the deconvoluted mass spectrum of FLAG-*T. castaneum* methoprene-tolerant shows a series of +80 Da mass additions consistent with up to six phosphorylation events (+1-6xPhos) per molecule. *B*, the major mass of His_6_-TcTAI (42617.2 Da) matches the theoretical mass with loss of the N-terminal methionine (–Met1 = −131 Da) and additional masses indicating acetylation (+Ac = +42 Da), phosphorylation (+80 Da), and a combination thereof (+Ac+Phos = +122 Da). *C* and *D*, treatment of purified *T. castaneum* JH receptor with thermosensitive alkaline phosphatase (TSAP) largely removed phosphoryl groups from FLAG-*T. castaneum* methoprene-tolerant and effectively removed them from His_6_-TcTAI with associated shift in the retention time of the dephosphorylated proteins. Acetylation of His_6_-TcTAI is not affected, and up to two phosphorylation sites on FLAG-*T. castaneum* methoprene-tolerant appear to be partially resistant to TSAP treatment under the conditions (see the Experimental procedures section). ESI, electrospray ionization; TcTAI, *T. castaneum* taiman; aTSAP, thermosensitive alkaline phosphatase.

To determine whether the purified JHR protein complexes retained the expected JH-binding function, we adapted a method based on capturing of hormone–receptor protein complexes on GF/C glass fiber filter discs, previously employed in the assay of [^3^H]-ponasterone A binding to EcR complexes (51). The results of this procedure were comparable to those obtained by dextran-coated charcoal adsorption, a method originally used to assess binding of [^3^H]-JH III to proteins that specifically bind JH (22, 23, 52). Data in Figure 4*A* show that the purified JHR proteins produced in *Sf9* cells from the baculovirus constructs bound the [^3^H]-labeled native hormonal ligand, JH III. The JH III-binding capacity of *T. castaneum* JH receptor was slightly reduced (by 13%; *p* < 0.05) after treatment with thermosensitive alkaline phosphatase (TSAP) (Fig. 4*A*), which resulted in dephosphorylation of the protein (Fig. 5). In a separate experiment, we tested the *T. castaneum* JH receptor protein complex, which was partially purified from *Sf9* cell lysates through a single affinity chromatography step, for effects of prolonged storage and freezing/thawing on ligand binding. As shown in Figure 4*B*, *T. castaneum* JH receptor retained its hormone-binding activity whether kept for 3 days at 4 °C or thawed after 1 month at −80 °C, albeit the frozen sample lost about 30% of its activity. Background binding in the GF/C-based assay was demonstrated to be low by use of a *T. castaneum* methoprene-tolerant protein bearing a mutation V297F in its ligand-binding pocket that prevents JH III from binding to the PAS-B domain of *T. castaneum* methoprene-tolerant (23) (Fig. 4*B*).

### Distribution and identity of phosphorylation sites of the *T. castaneum* JH receptor complex

To resolve post-translational modifications and study the composition of *T. castaneum* JH receptor complexes in more detail, we analyzed the intact mass of *T. castaneum* methoprene-tolerant and TcTAI subunits under denaturing conditions using LC–electrospray ionization (ESI)–MS. The deconvoluted mass spectrum of the FLAG-*T. castaneum* methoprene-tolerant subunit showed a series of +80 Da mass additions consistent with up to six phosphorylations per molecule (Fig. 5*A*). The major mass of the His_6_-TcTAI subunit indicated the loss of N-terminal methionine with prominent acetylation and phosphorylation modifications (Fig. 5*B*). Treatment of purified *T. castaneum* JH receptor with TSAP reduced FLAG-*T. castaneum* methoprene-tolerant phosphorylation by at least 50%, with an associated shift in the retention time of dephosphorylated proteoforms (Fig. 5, *A* and *C*). At least two phosphorylation sites of FLAG-*T. castaneum* methoprene-tolerant appeared to be resistant to the TSAP treatment (Fig. 5*C*), which effectively removed phosphorylation of the His_6_-TcTAI protein (Fig. 5, *B* and *D*).

To identify potential phosphorylation sites, we also performed LC–MS/MS analyses of the purified recombinant *T. castaneum* JH receptor complex. Mascot database searches yielded 53% coverage for FLAG-*T. castaneum* methoprene-tolerant and 64% sequence coverage for His_6_-TcTAI, respectively (Table 1). For confident Mascot peptide identifications, see Table S1, annotated phosphopeptide MS/MS evidence spectra are shown in Fig. S4, and corresponding LC–MS/MS data are available from ProteomeXchange under the identifier PXD028394. The analysis uncovered at least ten potentially phosphorylated residues, predominantly clustered in the C-terminal region of FLAG-*T. castaneum* methoprene-tolerant (Tables 1 and S1; Fig. S4).

**Table 1:** Phosphosites identified by shotgun proteomics analysis in the recombinant *T. castaneum* JH receptor complex

Protein	Accession^a^	Sequence	Molecular weight (Da)	Amino acids	Coverage	Spectra^b^	Peptides^b^
FLAG-*T. castaneum* methoprene-tolerant	A6MUT7	M1-V516	59542.3	279/526	53%	51	24

Phosphopeptide sequence^c^	Phosphosites	*m/z*	Charge	Mass^d^	ΔMass [ppm]^e^	Score^f^	ΔScore^f^
VYGNVNENQDCSTPT(ph)ENSPTKPYYK	T438	995.76	3	2984.254	0.16	23.6	1.9
VYGNVNENQDCSTPTENS(ph)PTKPYYK	S441	747.07	4	2984.247	−2.19	50.9	8.6
RPPS(ph)TELGTNIYTSSK	S459	610.96	3	1829.847	−5.15	74.2	7.9
RPPST(ph)ELGTNIYTSSK	T460	610.96	3	1829.845	−5.89	71.3	4.7
QRTS(ph)PQLSPMSSLPPYPNR	S476	746.02	3	2235.050	−0.21	32.6	1.1
TSPQLS(ph)PMSSLPPYPNR	S480	976.45	2	1950.891	−0.10	79.2	34.7
TSPQLS(ph)PM(ox)SSLPPYPNR	S480	656.63	3	1966.880	−3.34	30.2	3.0
QRTSPQLSPM(ox)S(ph)S(ph)LPPYPNR	S503, S504	778.02	3	2331.024	5.27	42.1	4.1

Protein	Accession^a^	Sequence	Molecular weight (Da)	Amino acids	Coverage	Spectra^b^	Peptides^b^
His_6_-TcTAI	S6B9A5	M42-A411	42757.9	224/380	64%	36	19

Phosphopeptide sequence^c^	Phosphosites	*m/z*	Charge	Mass^d^	ΔMass (ppm)^e^	Score^f^	ΔScore^f^
KKS(ph)ETKPQAQINK	S56	395.7105	4	2331.024	−0.16	37.7	1.3

a UniProt identifier.

b Numbers of unique spectra and unique peptides, respectively.

c S(ph) and T(ph), phosphorylation sites; M(ox), oxidized methionine residues.

d Observed precursor mass.

e Mass error in parts per million.

f Mascot score and Mascot delta score, respectively.

### Methoprene-dependent phosphorylation of *T. castaneum* methoprene-tolerant and its protein interactions

**Figure 6:**
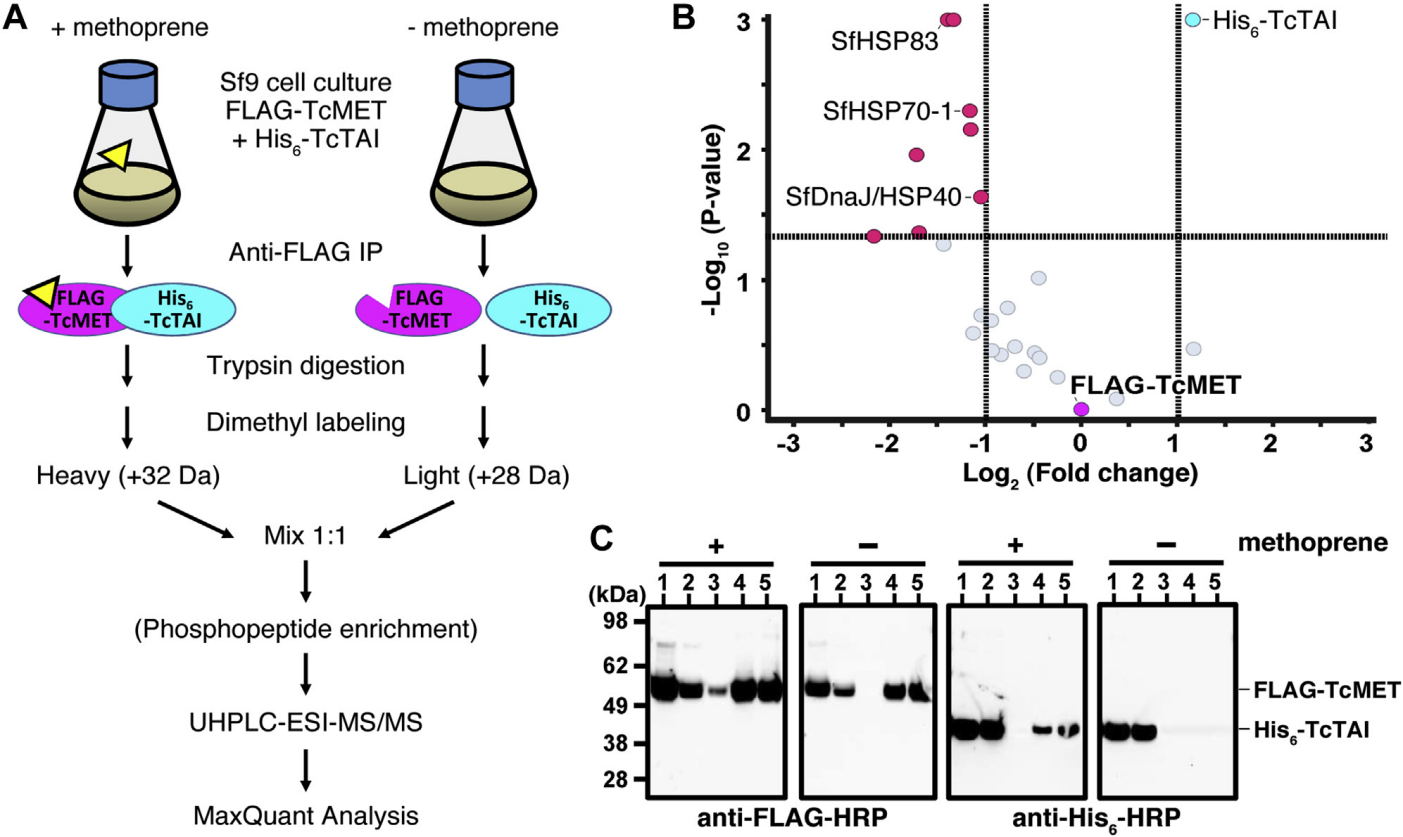
**Affinity-MS and quantitative phosphoproteomics analysis of*****T. castaneum*****JH receptorcomplexes.***A*, quantitative proteomics workflow to identify methoprene-dependent protein interactions and phosphorylation sites of *T. castaneum* JH receptor using a stable isotope dimethyl labeling approach. Anti-FLAG pull downs of *T. castaneum* JH receptor complexes expressed in baculovirus-infected *Sf9* cells in the presence (+) or the absence (−) of methoprene were prepared as detailed in the Experimental procedures section. A 1:1 mixture of tryptic peptides of *T. castaneum* JH receptor pull downs prepared from “heavy” (H; +32 Da)-labeled methoprene-supplemented or “light” (L; +28 Da)-labeled methoprene-free cultures was generated, and a TiO_2_-enriched phosphopeptide sample was prepared and analyzed by UHPLC–ESI–MS/MS on an Orbitrap Fusion Lumos Tribrid mass spectrometer. MaxQuant database searches facilitated confident identification, phosphosite localization, and quantification of proteins or peptides as detailed in the Experimental procedures section. *B*, a volcano plot shows a greater than twofold enrichment of His_6_-TcTAI associated with FLAG-*T. castaneum* methoprene-tolerant in the presence of methoprene. By contrast, methoprene seems to promote the dissociation of some *Sf9* proteins including HSP83, HSP70-1, and a DnaJ/HSP40-like protein (see Table S2 for details). *C*, immunoblots show roughly equivalent levels of FLAG-*T. castaneum* methoprene-tolerant present in input (lane 1), unbound (lane 2), void (lane 3), and elution fractions (lanes 4 and 5) in the presence (+) or the absence (−) of methoprene. The same immunoblots reprobed with HRP-conjugated anti-His_6_ antibody show a significant reduction in the amount of copurified His_6_-TcTAI in the absence of methoprene (lanes 4 and 5, anti-His_6_-HRP). ESI, electrospray ionization; HRP, horseradish peroxidase; TcTAI, *T*. *castaneum* taiman.

In order to determine if methoprene directly or indirectly mediates *T. castaneum* methoprene-tolerant phosphorylation and/or regulates stable protein interactions of the *T. castaneum* JH receptor, we designed the quantitative proteomics experiment illustrated in Figure 6*A*. Briefly, anti-FLAG pull downs of *T. castaneum* JH receptor complexes expressed in baculovirus-infected *Sf9* cells in the presence or the absence of 5 μM methoprene were prepared. A 1:1 mixture of tryptic digests generated from pull downs of “heavy” (H; +32 Da)-labeled, methoprene-supplemented, or “light” (L; +28 Da)-labeled methoprene-free cell cultures was then analyzed by nano-flow UHPLC–ESI–MS/MS on an Orbitrap Lumos mass spectrometer, as detailed in the Experimental procedures section. MaxQuant database searches confidently identified (false discovery rate [FDR] <1%) several proteins that were significantly enriched (H/L ratio < 0.5; *p* < 0.05) in pull downs prepared in the absence of methoprene (Fig. 6*B, red* and Table S2). With FLAG-*T. castaneum* methoprene-tolerant serving as an internal control with a normalized H/L ratio of 1:1, these included *Sf9* homologs of the multimeric heat shock protein HSP90 chaperone complex, namely HSP83 (UniProt: Q9GQG6), HSP70A1 (UniProt: A0A0K2CTM7), and a DnaJ/HSP40-like protein (UniProt: V5ND41). By contrast, the only protein significantly enriched (H/L ratio > 2; *p* < 0.05) in pull downs with methoprene was His_6_-TcTAI (Fig. 6*B, cyan* and Table S2). The over twofold enrichment of His_6_-TcTAI was confirmed using immunoblots of anti-FLAG pull downs (Fig. 6*C*). *Sf9* host cell proteins that could be detected at similar levels in both anti-FLAG pull downs (Fig. 6*B*, *gray*) or could not be reliably quantified are listed in Table S2. Together, these results demonstrate that the addition of the JHR agonist methoprene to growth media reduces interaction(s) of *T. castaneum* methoprene-tolerant with the host cell HSP83 chaperone complex and promotes the interaction between *T. castaneum* methoprene-tolerant and TcTAI. This results in an overall increase in the yield and stability of purified *T. castaneum* JH receptor receptor complexes in the presence of methoprene.

**Figure 7:**
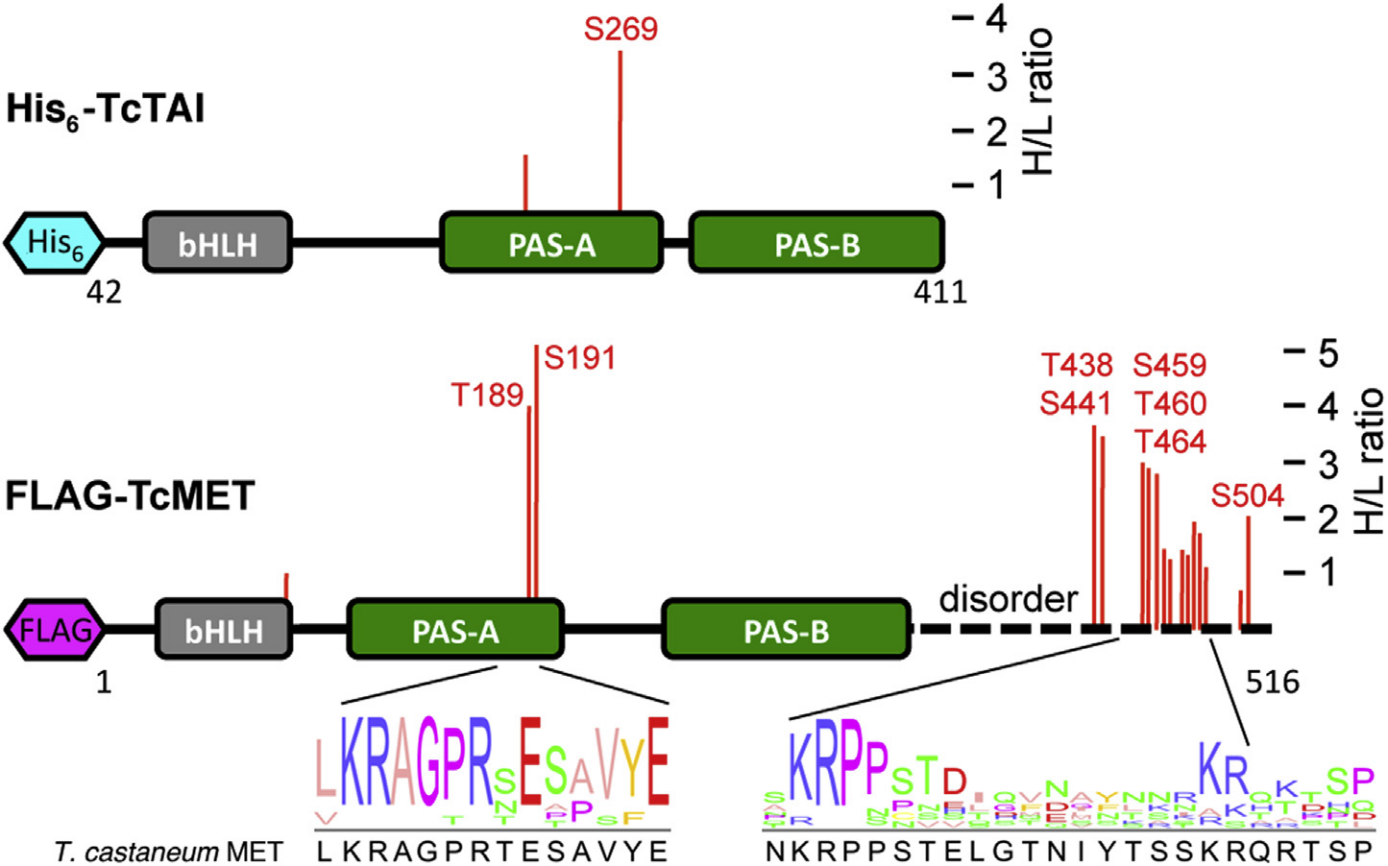
**Mapping of methoprene-dependent phosphorylation sites on the secondary structure of*****T. castaneum*****JH receptorsubunits.** Distribution and H/L ratio of methoprene-dependent phosphorylation sites within the domains of the His_6_-TcTAI and FLAG-*T. castaneum* methoprene-tolerant proteins based on quantitative phosphoproteomics data in Table 2. The positions of the phosphorylated residues are indicated by *red bars* with the height of the bar proportional to the H/L ratio; methoprene-dependent phosphorylation sites with H/L ratio >2 are numbered. A putative bipartite nuclear localization signal is predicted within the intrinsically disordered C-terminal region (*broken line*) of *T. castaneum* methoprene-tolerant. The diagrams at the *bottom* show conservation of the sequence contexts around the C-terminal region and PAS-A phosphorylated residues among nine beetle species (see Fig. S6*A* for details). TcTAI, *T*. *castaneum* taiman.

To determine the effect of methoprene on the phosphorylation state of affinity-purified *T. castaneum* JH receptor complexes, we also performed quantitative proteomics analyses of TiO_2_-enriched phosphopeptide fractions (Fig. 6*A*), as detailed in the Experimental procedures section. This approach identified up to 17 possible phosphorylation sites (FDR <1%, location probability >50%) of FLAG-*T. castaneum* methoprene-tolerant and two of the truncated His_6_-TcTAI protein (Table 2). Phosphorylation sites of *T. castaneum* JH receptor complexes mapped to the PAS-A domains of TcTAI (S269) and *T. castaneum* methoprene-tolerant (T189 and S191) and to a cluster of serine and threonine residues in the C-terminal disordered region of *T. castaneum* methoprene-tolerant (Table 2 and Fig. 7). Supporting MaxQuant phosphopeptide data are listed in Table S3, and corresponding annotated MS/MS evidence spectra are shown in Fig. S5. The quantitative phosphoproteomics data are available from ProteomeXchange (identifier: PXD028599).

**Table 2:** Identification of methoprene-dependent phosphorylation sites in the affinity-purified *T. castaneum* JH receptor complex

Protein	Accession	Sequence	Molecular weight (Da)	Amino acids	Coverage	Spectra	Peptides	H/L ratio^a^
FLAG-*T. castaneum* methoprene-tolerant	A6MUT7	M1-V516	59542.3	295/526	56.0%	696	26	0.9 ± 0.4

Phosphopeptide sequence	Phosphosite^b^	*m/z*	Charge	ΔMass (ppm)	Score^c^	ΔScore^c^	Probability^d^	H/L ratio
IYQTLLS(ph)GK	S86	579.81	2	−0.06	60.5	57.8	99.6%	1.0
T(ph)ESAVYEPVR	T189	629.79	2	0.03	150.0	150.0	61.6%	4.0
TES(ph)AVYEPVR	S191	632.81	2	0.41	220.5	208.7	99.5%	5.1
VYGNVNENQDCSTPT(ph)ENSPTK	T438	1030.16	3	−0.22	90.1	73.2	96.1%	3.6
VYGNVNENQDCSTPTENS(ph)PTK	S441	1024.12	3	0.72	106.4	79.3	90.1%	3.4
RPPS(ph)TELGTNIYTSSK	S459	943.97	2	−0.01	248.5	248.5	98.4%	3.0
RPPST(ph)ELGTNIYTSSK	T460	944.47	2	−0.25	113.5	85.3	84.6%	2.9
RPPSTELGT(ph)NIYTSSK	T464	633.67	3	−0.44	69.0	53.9	79.8%	2.8
RPPSTELGTNIYTS(ph)SK	S469	629.65	3	−0.10	124.2	124.2	59.4%	1.5
RPPSTELGTNIYTSS(ph)K	S470	629.98	3	0.02	99.1	99.1	81.6%	1.3
T(ph)SPQLSPMSSLPPYPNR	T475	990.47	2	0.32	298.3	270.0	100.0%	1.5
T(ph)SPQLS(ph)PMSSLPPYPNR	T475, S480	1033.47	2	0.24	252.0	243.2	99.5%	1.9
QRTS(ph)PQLSPMSSLPPYPNR	S476	1136.07	2	−1.11	199.9	149.9	99.5%	1.4
QRTS(ph)PQLSPMS(ph)SLPPYPNR	S476,S483	1176.05	2	0.04	127.9	127.9	90.1%	1.7
TSPQLSPM(ox)SS(ph)LPPYPNR	S484	998.97	2	−0.08	133.5	125.2	84.9%	1.1
TQITEVTQQT(ph)EPSIYQHDQLLR	T501	913.12	3	0.04	73.3	72.6	87.5%	0.7
TQITEVTQQTEPS(ph)IYQHDQLLR	S504	913.12	3	0.10	127.4	123.7	99.3%	2.0

Protein	Accession	Sequence	Molecular weight (Da)	Amino acids	Coverage	Spectra	Peptides	H/L ratio^a^
His_6_-TcTAI	S6B9A5	M42-A411	42757.9	330/380	86.8%	484	31	2.1 ± 0.7

Phosphopeptide sequence	Phosphosite^b^	*m/z*	Charge	ΔMass (ppm)	Score^c^	ΔScore^c^	Probability^d^	H/L ratio
SRS(ph)FSVR	S227	473.73	2	0.04	66.27	45.75	93.1%	1.5
DQVS(ph)VSDDDGADSGPYLLCVASR	S269	1271.06	2	−0.14	126.88	126.88	95.2%	3.4

Other categories as in Table 1.

a Normalized ratio of the heavy to light phosphopeptide label partner.

b Phosphorylation sites that are more than twofold enriched in the presence of methoprene (H/L ratio > 2) are underlined.

c MaxQuant score and MaxQuant delta score, respectively.

d MaxQuant phospho (S/T) site probability (cutoff of >50%).

Some peptide sequences isolated from this disordered region contained up to five potential phosphorylation sites and were also found to be doubly phosphorylated, making the confident localization and relative quantitation of individual phosphorylation sites more difficult. Based on the calculated H/L ratios in Table 2, the relative intensity of most phosphorylation sites appeared to change little in the presence or the absence of methoprene. However, we were able to identify up to nine distinct phosphorylation sites in the affinity-purified *T. castaneum* JH receptor preparations that appeared to be more than twofold enriched by the presence of methoprene. These included TcTAI residue S269 and *T. castaneum* methoprene-tolerant residues T189/S191 in the PAS-A domains, as well as *T. castaneum* methoprene-tolerant residues S438, S441, S459, T460, T464, and S504, in the disordered C-terminal region (Table 2 and Fig. 7). Some of the phosphorylation sites within the PAS-A domain and the disordered C-terminal region of *T. castaneum* methoprene-tolerant are conserved in sequence contexts of MET orthologs from other beetle species (Figs. 7 and S6*A*).

### Nuclear localization of *T. castaneum* methoprene-tolerant

**Figure 8:**
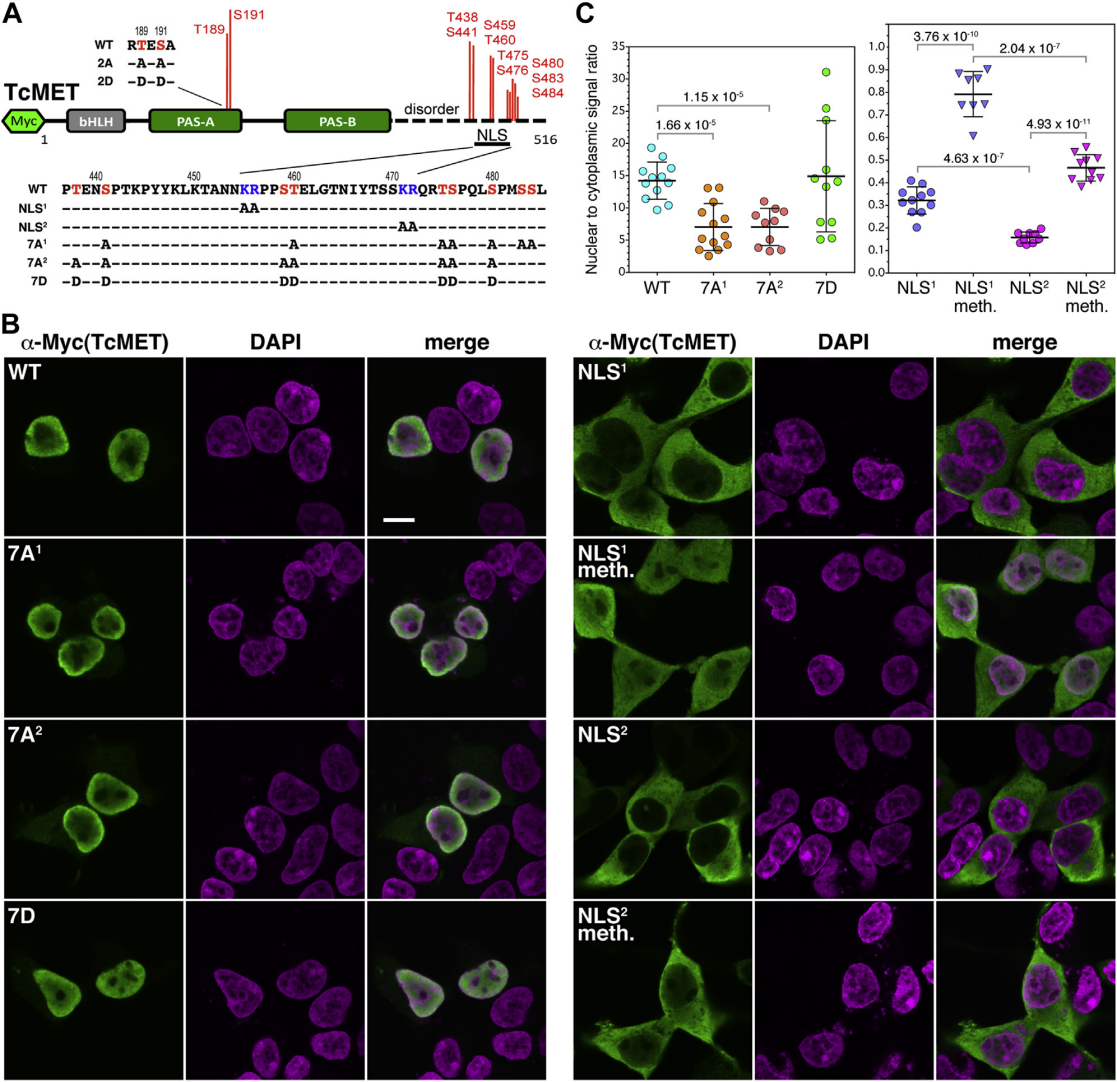
**Effects of mutations and methoprene on the cellular localization of*****T. castaneum*****methoprene-tolerant.***A*, mutated variants of *T. castaneum* methoprene-tolerant. Basic residues (KR, in *blue*) constituting the basic clusters of a bipartite NLS were mutated to A (variants NLS^1^ and NLS^2^). Subsets of residues that are subject to methoprene-dependent or independent phosphorylation (in *red*) were substituted either with alanine (variants 2A, 7A^1^, and 7A^2^) or with aspartic acid (2D and 7D). *B*, the WT *T. castaneum* methoprene-tolerant or its mutated variants, all carrying an N-terminal Myc epitope, were expressed in transfected HEK293 cells and detected using an anti-Myc antibody (*green*); DNA was stained with DAPI (*magenta*). Representative examples of Myc-*T. castaneum* methoprene-tolerant localization are single confocal slices. The scale bar represents 10 μm and applies to all images. *C*, ratios of nuclear-to-cytoplasmic Myc-*T. castaneum* methoprene-tolerant signal; each *circle* represents average of integrated pixel intensities from six measurements in individual transfected cells. Mutating the phosphorylable residues to alanine had a small but significant effect on reducing the predominance of nuclear localization. Removing either of the basic clusters of the NLS rendered *T. castaneum* methoprene-tolerant largely cytoplasmic; 1 μM methoprene induced a partial nuclear retention (visible in *B*) particularly of the NLS^1^ variant. The data are mean values with error bars representing SD; the *p* values (*t* test) signify differences of the indicated pairwise comparisons. DAPI, 4′,6-diamidino-2-phenylindole; HEK293, human embryonic kidney 293 cell line; meth., 1 μM methoprene; NLS, nuclear localization signal.

A majority of the identified phosphorylation sites in the C-terminal sequence of *T. castaneum* methoprene-tolerant straddle a potential bipartite nuclear localization signal (NLS) (Fig. 7), predicted by the cNLS Mapper program (53). We first examined the function of the *T. castaneum* methoprene-tolerant putative bipartite NLS itself by mutating to alanine the basic lysine and arginine residues that form the predicted basic clusters of the motif; the resulting *T. castaneum* methoprene-tolerant variants were designated NLS^1^ and NLS^2^, respectively (Fig. 8*A*). The WT and mutant *T. castaneum* methoprene-tolerant proteins carrying an N-terminal Myc epitope were expressed in human embryonic kidney 293 (HEK293) cells and stained with an anti-Myc antibody. While the WT protein localized to the nucleus, both NLS^1^ and NLS^2^ showed a strong predominantly cytoplasmic signal (Fig. 8, *B* and *C*), suggesting that both the basic clusters are essential for the NLS function. Quantification of nuclear-to-cytoplasmic ratios of signal intensities in confocal micrographs of multiple individual cells revealed a greater residual nuclear signal in the case of the NLS^1^ variant relative to NLS^2^ variant (Fig. 8*C*). Mutating the distal basic cluster thus appeared to have a stronger impact. Moreover, while treatment with methoprene stimulated a significant shift of both mutated variants to the cell nuclei, the translocation of the NLS^1^ *T. castaneum* methoprene-tolerant protein was more pronounced (Fig. 8, *B* and *C*). To examine TcMET localization in a homologous system, we utilized a TcA cell line derived from *T. castaneum*. Although morphology of the beetle cells did not permit signal quantification from confocal images, we were able to corroborate the function of the bipartite NLS and the agonist effect, in this case of JH III, causing partial nuclear import of the NLS^1^ mutated *T. castaneum* methoprene-tolerant protein (Fig. S7).

To see if the loss of phosphorylation or if mimicking its constitutive gain in the C-terminal region of *T. castaneum* methoprene-tolerant might affect its intracellular localization, we prepared *T. castaneum* methoprene-tolerant variants 7A^1^, 7A^2^, and 7D, in which subsets of seven of the identified serine and threonine phosphorylated residues were replaced either with alanine to mimic phosphorylation loss or with aspartic acid to emulate constitutive phosphorylation (Fig. 8*A*). All three mutated *T. castaneum* methoprene-tolerant variants predominantly localized in the nuclei of HEK293 as well as the TcA cells (Figs. 8*B* and S7), suggesting that unlike loss of the NLS itself, removal of phosphorylation in its proximity did not prevent nuclear import. However, quantification of signal intensities in the HEK293 cell line showed that loss of the phosphorylation sites caused a minor but significant retention of the 7A^1^ and 7A^2^ mutant proteins in the cytoplasm (Fig. 8*C*). By contrast, the localization of the phosphomimetic 7D variant of *T. castaneum* methoprene-tolerant did not differ from that of the WT protein.

### Phosphorylation status of JHR proteins and their JHRE-binding and JH-binding capabilities

**Figure 9:**
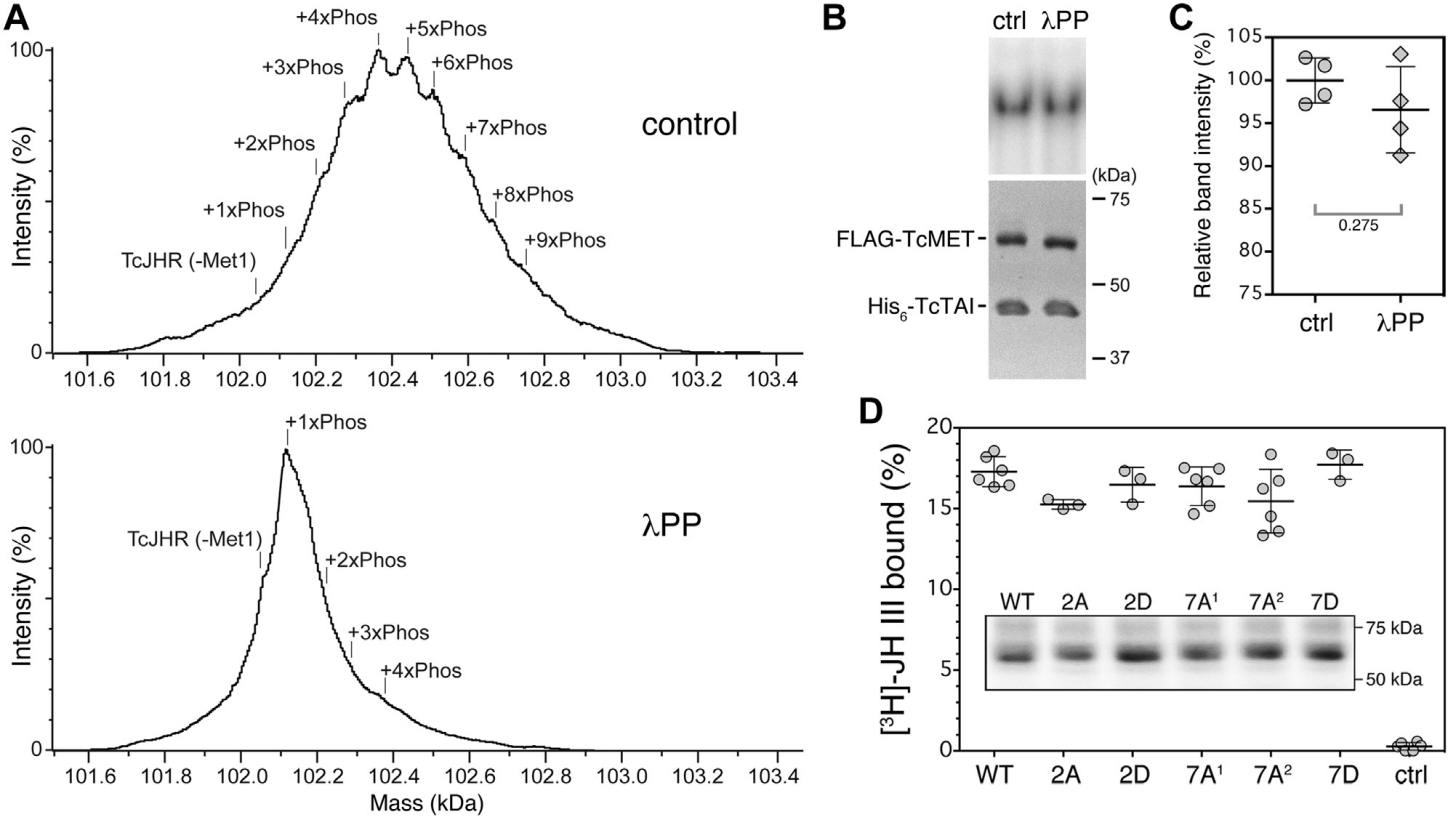
**Dephosphorylated*****T. castaneum*****JH receptorretains the capacity to bind JHRE DNA and juvenile hormone (JH).***A*, dephosphorylation of the recombinant *T. castaneum* JH receptor protein complex purified from the *Sf9* cells as confirmed through native MS analysis after incubation with buffer alone (control) or with 40 U/μl of lambda protein phosphatase (λPP) for 1 h at 4 °C. Deconvoluted native MS spectra show multiple and partially resolved +80 Da mass additions (+1-8xPhos) to the *T. castaneum* JH receptor complex (*top*). λPP treatment caused a shift in mass distribution that is consistent with the effective removal of phospho modifications without affecting the structural integrity of *T. castaneum* JH receptor complexes (*bottom*). *B*, EMSA was performed with the *T. castaneum* JH receptor complex. Prior to incubation with the *k*-JHRE DNA, the protein samples were treated with 40 U/μl of λPP or buffer alone (ctrl) for 1 h at 4 °C. Integrity of the proteins following the treatment was verified on a Coomassie-stained SDS-PAGE (*bottom*). *C*, quantification of band-integrated densities determined from four parallel assays revealed a nonsignificant reduction of the signal upon λPP treatment. The data are mean values with error bars representing SD; the *p* value was calculated by *t* test. *D*, WT Myc-*T. castaneum* methoprene-tolerant and its variants with mutated phosphorylable sites (shown in Fig. 8*A*) were *in vitro* translated using the T7-coupled rabbit reticulocyte lysate system; control (ctrl) contained the reticulocyte lysate without DNA input. The translation products were verified through immunoblotting with an anti-Myc antibody (inset) and subjected to [^3^H]-JH III binding assays. The mutations had no significant effect on JH binding. JHRE, juvenile hormone response element.

To test if dephosphorylation of the purified baculoviral *T. castaneum* JH receptor protein complex might affect its binding to the JHRE target DNA, we performed the EMSA as described previously except that the protein was incubated with or without lambda serine/threonine protein phosphatase (λPP) prior to the assay. The relatively mild treatment with λPP caused a ∼320 Da reduction in the average molecular weight of *T. castaneum* JH receptor with little or no effect on the stability of the partially dephosphorylated *T. castaneum* methoprene-tolerant–TcTAI complex as determined by native MS (Fig. 9*A*). Although λPP was effective in removing most of the detectable phosphoryl groups from the *T. castaneum* JH receptor complex, the treatment did not prevent it from interacting with the JHRE (Fig. 9*B*). Signal intensities of the retarded bands, as quantified from parallel EMSA experiments, did not show a significant decrease upon λPP treatment relative to incubation in the phosphatase buffer alone (Fig. 9*C*). The purified AaMET–AaTAI complex was likewise capable of binding the JHRE following the treatment with λPP (Fig. S8). These data suggest that dephosphorylation had no major effect on the capacity of the purified recombinant JHR complexes to bind the JHRE DNA.

To examine whether phosphorylation might influence the JH-binding activity of *T. castaneum* methoprene-tolerant, we employed the aforedescribed mutations 7A^1^, 7A^2^, and 7D along with *T. castaneum* methoprene-tolerant variants 2A and 2D, where the T189 and S191 residues in the PAS-A domain, both phosphorylated in response to methoprene in *Sf9* cells, were again replaced with either alanine or aspartic acid (Fig. 8*A*). All five mutated *T. castaneum* methoprene-tolerant proteins and the WT control were produced using an *in vitro* translation system and tested for binding of [^3^H]-JH III. All the *T. castaneum* methoprene-tolerant variants appeared to be expressed at comparable levels and were stable for the assay duration based on immunoblotting using their N-terminal Myc epitope (Fig. 9*D*). Their capacity to bind the radiolabeled hormone was similar to that of the WT protein (Fig. 9*D*), indicating that the phosphorylation status of the tested residues had no effect on the JH-binding function, which is located in the PAS-B domain of *T. castaneum* methoprene-tolerant. This result is consistent with our earlier observation that the ability of the *T. castaneum* JH receptor complex, purified from the baculovirus/*Sf9* cell system, to bind [^3^H]-JH III was little affected by treatment with the alkaline phosphatase TSAP (Fig. 4*A*).

## Discussion

Evidence converging from multiple systems indicates that a complex between the bHLH–PAS proteins MET and TAI forms a functional JHR in insects (21, 23, 25, 33, 39, 40, 42, 44). Here, we report successful high-yield expression and purification of recombinant MET–TAI JHR complexes from cultured insect cells and evidence of their protein subunit stoichiometry and specific binding to JH III and JHREs. We also characterize post-translational modifications of *T. castaneum* methoprene-tolerant, its protein–protein interactions, and nuclear localization as influenced by the JHR agonist methoprene. Partial purification of recombinant AaMET and AaTAI proteins expressed in *Escherichia coli* was reported by Li *et al.* (24), but the more homologous baculovirus-mediated expression in insect cells described here clearly has significant advantages particularly for the study of post-translational modifications and of functional properties influenced by such modifications.

### Stoichiometry of the MET–TAI complex

Although MET and TAI are assumed to form a functional JHR heterodimer (39), the stoichiometry has so far not been experimentally validated. Based on SEC–MALLS, the molecular weight of our purified *T. castaneum* JH receptor complex is 103.8 ± 2.1 kDa (Fig. 2*A*). This was confirmed by native MS, which detected a stable complex of 102.6 ± 0.5 kDa (Fig. 2, *C* and *D*). Comparison with theoretical molecular weights of the FLAG-*T. castaneum* methoprene-tolerant–His_6_-TcTAI heterodimer (102.5 kDa), FLAG-*T. castaneum* methoprene-tolerant homodimer (119.5 kDa), and His_6_-TcTAI homodimer (85.5 kDa) clearly indicates that our purified recombinant *T. castaneum* JH receptor is the heterodimer. Under optimized conditions, deconvoluted native MS spectra showed multiple and partially resolved proteoforms exhibiting +80 Da [HPO_3_] mass additions (+1-8xPhos) to the *T. castaneum* JH receptor complex (Fig. 9*A*). This observation is consistent with high-resolution LC–ESI–MS results demonstrating up to six +80 Da mass additions of *T. castaneum* methoprene-tolerant plus a single phosphorylation of TcTAI (Fig. 5, *A* and *B*).

### Interaction of MET with TAI, HSP83, and other proteins

Our data showed that the *T. castaneum* JH receptor complex is stabilized by the presence of the JHR agonist methoprene. First, removal of methoprene by dialysis led to aggregation of the *T. castaneum* methoprene-tolerant–TcTAI dimer as assessed using SEC–MALLS (Fig. S2*A*). Second, MS of proteins that had been pulled down with FLAG-*T. castaneum* methoprene-tolerant from the *Sf9* cells identified His_6_-TcTAI as strongly enriched in the presence of methoprene. This ligand-dependent association between *T. castaneum* methoprene-tolerant and TcTAI is in agreement with previous demonstrations that native or synthetic JHR agonists including methoprene induced interactions between the JHR subunits, either in coimmunoprecipitation (23) or two-hybrid assays (26, 33, 39, 54, 55).

In addition to TcTAI, *T. castaneum* methoprene-tolerant interacted with proteins naturally occurring in the *Sf9* host cells, most notably the HSP83, orthologous to vertebrate HSP90. The chaperone HSP90/83 is well known to associate with and support function of nuclear hormone receptors such as the glucocorticoid receptor (56) or the *D. melanogaster* EcR (57). Interestingly, HSP90 is important for the function of the mammalian homolog of MET, the ligand-activated transcription factor of the bHLH–PAS family, aryl hydrocarbon receptor (AHR) (58, 59). Without an agonist ligand, HSP90 binds the bHLH and PAS-B domains of AHR and stabilizes AHR within a transcriptionally inactive cytoplasmic complex (60). In the presence of an activating ligand, HSP90 facilitates nuclear import of the AHR-containing complex (61). Once in the nucleus, liganded AHR dissociates from HSP90 and forms a DNA-binding transcriptionally active heterodimer with its bHLH–PAS partner ARNT (60, 62, 63, 64).

In *D. melanogaster*, HSP83 has been shown to mediate methoprene-stimulated nuclear import of MET and the transcriptional induction of the *Kr-h1* gene (65), suggesting that the JHR–HSP83 interaction is functionally conserved with the homologous AHR–HSP90 mechanism. Surprisingly, He *et al.* (65) detected HSP83 among proteins bound to a JHRE sequence upstream of *Kr-h1* in *D. melanogaster* cells cultured with methoprene, leading to a model in which HSP83 becomes part of a transcriptionally active DNA-bound complex containing MET. This model is inconsistent with the mechanism of AHR action, in which agonist binding to the PAS-B domain of AHR displaces HSP90, which, therefore, does not participate in the active AHR–ARNT complex (60, 62, 64). In contrast, our data show that when expressed in the *Sf9* cells, *T. castaneum* methoprene-tolerant preferentially associated with HSP83 in the absence of methoprene. When methoprene was added to the cell culture, *T. castaneum* methoprene-tolerant dissociated from HSP83 and instead dimerized with TcTAI (Fig. 6). Therefore, our results concur with the established mode of AHR action. The proteomics analysis of complexes pulled down with FLAG-*T. castaneum* methoprene-tolerant from *Sf9* cells revealed several additional proteins of interest, albeit most were insufficiently abundant to be quantified with statistical significance (Table S2).

### Nuclear import of MET

While the *D. melanogaster* MET and GCE proteins predominantly reside in cell nuclei when expressed in heterologous cell lines or in insect tissues (22, 25, 65, 66, 67, 68, 69), JHR agonists further enhance their nuclear localization. Both of the fly paralogs contain multiple NLS motifs, which confer nuclear localization of MET and GCE either constitutively or in response to JH (68, 69, 70). However, signals for nuclear import of JHR proteins have not been identified in insects other than *D. melanogaster*.

We have discovered in the C-terminal portion of *T. castaneum* methoprene-tolerant, a functional bipartite NLS, in which each of the two clusters of basic residues is required for the predominant nuclear localization of *T. castaneum* methoprene-tolerant. The nature and position of this motif correspond to a previously described NLS in *D. melanogaster* GCE (69). In both cases, the sequences match a consensus of the classical bipartite NLS, defined as (K/R)(K/R)X10-12(K/R)3/5, where X indicates any amino acid and (K/R)3/5 represents at least three of either lysine or arginine out of five consecutive residues (71); in this case, 14 residues separate the basic clusters in *T. castaneum* methoprene-tolerant. Alignment of the *T. castaneum* NLS with MET proteins from several other beetle species shows that the linker sequence may be shorter (10–12 residues) in some cases (Fig. S6*A*). While the C-terminal regions of insect MET/GCE proteins show little sequence homology, the cNLS Mapper tool (53) could predict in this region bipartite NLS motifs matching the aforementioned consensus in species representing evolutionarily distant insect orders, including the honeybee (Hymenoptera), the migratory locust (Orthoptera), the German cockroach (Blattodea), or the linden bug (Hemiptera) (Fig. S6*B*). In contrast, a sequence separating the basic clusters in a potential bipartite NLS of AaMET is double the consensus length. No NLS has been found in the carboxyl terminal region of the *D. melanogaster* MET protein (68), which is derived from the ancestral GCE paralog.

Mutating either one of the basic clusters in *T. castaneum* methoprene-tolerant rendered the protein predominantly cytoplasmic. Addition of methoprene induced partial relocation to the nucleus, which was more appreciable for the NLS^1^ mutation affecting the proximal KR cluster, whereas the nuclear-to-cytoplasmic ratio was significantly lower for the NLS^2^ variant (Fig. 8*C*). The weaker effect of the NLS^1^ mutation relative to NLS^2^ could be explained by residual activity of the mutated NLS whose distal basic cluster KRQRT matches a consensus monopartite NLS sequence K(K/R)X(K/R) (71) as predicted using cNLS Mapper (53).

The C-terminal NLSs reside in proline/serine/threonine-rich regions that are intrinsically disordered in both *D. melanogaster* GCE (69, 72) and *T. castaneum* methoprene-tolerant proteins (Fig. 7). Our phosphoproteomics data revealed extensive serine/threonine phosphorylation within the disordered region of *T. castaneum* methoprene-tolerant. Some of the phosphorylated residues occur in close proximity to the basic clusters of the NLS, suggesting that phosphorylation might modulate the subcellular localization of *T. castaneum* methoprene-tolerant. While replacement of the identified Ser and Thr residues with the phosphomimetic Asp did not alter the nuclear localization of *T. castaneum* methoprene-tolerant, the variants lacking subsets of the phosphorylable sites were partially retained in the cytoplasm, although the effect was minor. This situation differs from the case of AHR, where phosphorylation of Ser residues straddling a bipartite NLS, located near the N terminus of AHR, inhibited nuclear import (73). Mammalian cell lines have clearly provided valuable experimental systems for demonstration of NLS activity of insect proteins in the present and other studies (68, 69, 70). However, future investigation of phosphorylation-mediated modulation of NLS activity in more homologous systems may be warranted.

### Constitutive and methoprene-induced JHR phosphorylation

JH exerts some of its effects *via* second-messenger signaling initiated at the cell surface (46, 74, 75). Intriguingly, JH III was shown in *A. aegypti* to trigger a pathway involving Ca^2+^/calmodulin-dependent kinase II and a protein kinase C downstream of phospholipase C (48, 76). The components of this pathway were required for JH to induce transcription of the mosquito *Kr-h1* and *ET* genes through the JHR subunits, AaMET and AaTAI, phosphorylated in the presence of JH III at multiple unidentified sites (48). A recent LC–MS/MS-based study has detected seven phosphoserine/phosphothreonine residues in recombinant AaMET, expressed in *A. aegypti* *Aag-2* cells (49). The phosphosites mostly occurred within a disordered region in the C-terminal third of the protein. Four of the identified AaMET residues became phosphorylated upon exposure of the *Aag-2* cells to JH III, whereas two phosphoserines were dephosphorylated, and one showed no response to the hormone (49).

Our analyses of the purified recombinant *T. castaneum* JH receptor complex revealed up to six phosphorylations per molecule of *T. castaneum* methoprene-tolerant and one in the truncated TcTAI protein (Fig. 5). This multiple phosphorylation was corroborated by native MS data detecting up to eight phosphosites in the *T. castaneum* JH receptor complex (Fig. 9*A*). Using shotgun LC–MS/MS, we identified at least ten potential phosphoserine and phosphothreonine residues within the C-terminal disordered region of *T. castaneum* methoprene-tolerant (Table 1). Quantitative phosphoproteomics of *T. castaneum* JH receptor pulled down from *Sf9* cells indicated that phosphorylation of at least two of the C-terminal serine residues in *T. castaneum* methoprene-tolerant increased upon exposure of the cells to methoprene. The same experiment revealed two additional, previously undetected, methoprene-induced phosphorylations in the PAS-A domains of both *T. castaneum* methoprene-tolerant and TcTAI proteins (Fig. 7). While the biological significance of these phosphorylation events remains to be investigated, it is clear that multiple phosphorylation, partly regulated by JHR agonists, is a common feature of MET proteins in *T. castaneum* and *A. aegypti*.

A threonine residue (T393) phosphorylated in response to JH III has recently been identified in the PAS-B domain of a MET1 protein from the cotton bollworm, *Helicoverpa armigera* (HaMET1) (77). Mutation to alanine (T393A) prevented HaMET1 from occupying an upstream region of the *Kr-h1* gene and from activating its expression, possibly because of a failure of HaMET^T393A^ to interact with HaTAI (77). Whether this might be a conserved mechanism of JHR regulation remains to be determined. The finding of the single T393 phosphorylation in HaMET1 contrasts with multiple phosphosites, detected through LC–MS/MS analyses in nonhomologous regions of either AaMET (49) or *T. castaneum* methoprene-tolerant (this study).

Previous work in *A. aegypti* (48) showed that chemical inhibition or RNAi knockdown of Ca^2+^/calmodulin-dependent kinase II reduced both the expression of the JH-inducible *Kr-h1* and *ET* genes and occupancy of their regulatory DNA by AaMET and AaTAI. When treated with λPP, nuclear protein extracts from female mosquito abdomens lost their capacity to form a retarded complex with the *ET* JHRE as assessed by EMSA, apparently suggesting that dephosphorylated AaJHR is incapable of binding its cognate DNA (48). It was therefore surprising to see that in similar EMSA experiments, our purified preparations of recombinant *T. castaneum* JH receptor and AaJHR complexes bound JHRE DNA even following λPP treatment (Figs. 9*B* and S8). Our treatment conditions were comparable to those employed by Liu *et al.* (48) except that we used 40 U/μl rather than 1 U/μl of λPP produced by the same company. Moreover, we have been able to verify dephosphorylation of the *T. castaneum* JH receptor complex by means of native MS (Fig. 9*A*). While we cannot exclude a critical role of residual phosphosites in *T. castaneum* JH receptor that might be inaccessible to λPP, our data strongly suggest that phosphorylation is not intrinsically required for binding of the *T. castaneum* methoprene-tolerant–TcTAI protein complex to DNA. The apparent discrepancy could be attributed to the fact that Liu *et al.* (48) worked with nuclear protein extracts from cultured mosquito abdomens, whereas we performed EMSA on defined reaction mixtures containing purified recombinant MET–TAI complexes. Two types of explanation reconciling the results from our laboratory and those of Liu *et al.* (48) involve potential interactions of JHR with proteins present in nuclear extracts. First, an interaction with a protein preventing the intrinsic ability of JHR to bind JHREs might be disrupted by phosphorylation. Second, an interaction favoring JHRE binding might be enhanced by phosphorylation. Our quantitative proteomics analyses have shown that *T. castaneum* JH receptor forms complexes with HSP83, HSP70-1, and DnaJ/HSP40-like proteins, all of which dissociate when cells are exposed to methoprene, concomitant with changes in JHR phosphorylation. It may be relevant that Soshilov *et al.* (64) observed transitional states in ligand-dependent transformation of the AHR into its DNA-binding form, accompanied by release of HSP90 from a transitional AHR-containing complex.

While much remains to be learned about post-translational regulation of JHR proteins, it is becoming clear that phosphorylation, subunit assembly, and cellular localization of JHR complexes play major roles in JH signaling. Our purification of active recombinant JHR protein complexes and their initial characterization pave the way for future functional and mechanistic studies.

## Experimental procedures

### Construction of baculoviruses for coexpression of recombinant MET and TAI proteins

To achieve coexpression of the two JHR subunits in *Spodoptera frugiperda Sf9* cells from a single construct, sequences encoding MET and TAI proteins either of *T. castaneum* or *A. aegypti* were cloned into the pFastBac Dual vector (Invitrogen). For the *T. castaneum* JH receptor, the sequence encoding a region from Met-42 to Ala-411 of TcTAI (National Center for Biotechnology Information [NCBI] accession number: XP_008193629.1) was custom synthesized (GenScript) to match the *S. frugiperda* codon usage. The synthetic TcTAI sequence was N-terminally fused with a hexahistidine tag and cloned into pFastBac Dual under the polyhedrin promoter. The complementary DNA sequence encompassing the entire *T. castaneum* methoprene-tolerant open reading frame from Met-1 to Val-516 (NCBI accession number: NP_001092812.1) was isolated from *T. castaneum* total RNA using reverse-transcription PCR (28), N-terminally fused to a FLAG tag and cloned under the p10 promoter in pFastBac Dual containing the TcTAI sequence. To prepare an AaJHR construct, appropriate DNA sequences were obtained using PCR from AaTAI and AaMET complementary DNA clones, kindly provided by Dr A. Raikhel (University of California, Riverside). A sequence spanning the region from Asn-100 to Glu-495 in AaTAI (NCBI accession number: ABE99837.2) was fused with an N-terminal FLAG tag and cloned under the p10 promoter. A sequence encoding the region from Lys-114 to Ser-977 in AaMET (NCBI accession number: AAX55681.1) was expressed with an N-terminal hexahistidine tag using the polyhedrin promoter.

The pFastBac Dual plasmid constructs containing the *T. castaneum* JH receptor and AaJHR encoding DNA sequences were transformed into the DH10Bac *E. coli* strain (Invitrogen) to produce recombinant bacmids. Once positive DH10Bac clones were obtained, bacmid DNA was prepared with the PureLink HiPure plasmid DNA kit (Invitrogen). The recombinant bacmid DNA was transfected into the *Sf9* cells using the Cellfectin II reagent (Invitrogen), and P1 viral stocks were generated according to the protocol provided by the system manufacturer.

### Virus amplification and insect cell culture

The recombinant baculovirus stocks were amplified by adding 1 ml of the initial P1 virus stock to 20 to 25 ml of a *Sf9*-II cell culture at 2 × 10^6^ viable cells/ml. The culture was harvested 4 to 5 days postinfection. Cells and cellular debris were removed by centrifugation (1500*g*, 15 min at 4 °C), and the supernatant sterile filtered using 0.2 μm Nalgene bottle top filters with 2% fetal bovine serum (FBS) added (Sigma). Virus stocks were then stored at 4 °C for up to 3 months. *Sf9*-II, *Sf9*-III, and Hi-5 cells (Life Technologies) were used for the production of receptor proteins from various constructs. Cells were incubated in E-flasks using serum-free media (SFM; Life Technologies), SF900-II SFM for *Sf9*-II and Hi-5 cells or Sf900-III SFM for *Sf9*-III cells, supplemented with 0.1% Pluronic (F68; Life Technologies) to minimize shear damage to cells. Growth temperature was set to 26.5 °C and shaking speed to 110 to 130 rpm.

### Scale-up and protein production

Optimal production conditions (best yield and biological activity) were determined in small-scale (50–100 ml E-flask cultures) experiments. Different cell lines (*Sf9*-II, *Sf9*-III, and Hi-5), multiplicity of infections (0.1–3), cell densities (in the range of 2–6 × 10^6^ viable cells/ml) and time of harvest (2–5 days postinfection) were tested, and the best conditions were selected for scale-up. Most often, the optimal scale-up parameters for protein expression were *Sf9*-III cells at 4 × 10^6^ viable cells/ml, with a multiplicity of infection of 1 and harvest taking place 72 h postinfection. Production scale, 6 to 12 l batches, were generated using Thomson or Corning shaker flasks (catalog no.: 931-116; catalog no.: 431-685) at 26.5 °C with 120 to 130 rpm shaking speed. Culture was supplemented with 1 μM methoprene (isopropyl (2*E*,4*E*,7*S*)-11-methoxy-3,7,11-trimethyldodeca-2,4-dienoate [Apollo Scientific]) at time of infection and a further 4 μM methoprene 2 h prior to harvest. Cultures were harvested by centrifugation (1000*g*, 10 min at 4 °C), and the pellets were washed twice in cold PBS containing 5 μM methoprene (cells were resuspended and pelleted again with centrifugation as aforementioned). Pellets were processed immediately for purification or snap frozen in liquid nitrogen and then stored at −70 °C.

### Purification of the *T. castaneum* JH receptor complex

Protein purification was achieved by resuspending frozen insect cell pellets in chilled lysis buffer at a ratio of 1 g cell pellet per 5 ml lysis buffer (50 mM Hepes, pH 7.5, 300 mM NaCl, 5 mM imidazole, 1 mM phenylmethylsulfonyl fluoride, complete EDTA-free protease inhibitors [Roche]; one tablet/50 ml lysis buffer), phosphatase inhibitor cocktail (1 mM NaF, 1 mM sodium pyrophosphate, 1 mM sodium tartrate, and 1 mM sodium molybdate), and 10 μM methoprene. Typically, 50 to 80 g of cell pellet were processed at a time. The cell suspension was lysed by two passes through an Emulsiflex-C5 homogenizer (Avestin) operating at 15,000 PSI at 4 °C. The lysate was clarified by centrifugation (25,000*g* for 30 min at 4 °C).

The supernatant containing the *T. castaneum* JH receptor complex was purified using a combination of immobilized nickel affinity chromatography (Ni-IMAC) and anti-FLAG affinity chromatography in the presence of 10 μM methoprene; the whole purification was completed within 1 day at 4 °C to minimize proteolysis and oxidation of both receptor subunits. Briefly, the clarified insect cell lysate was incubated with Ni–NTA superflow agarose (Qiagen) previously equilibrated with a binding buffer (50 mM Hepes, pH 7.5, 300 mM NaCl, and 10 μM methoprene) containing 5 mM imidazole in batch binding mode. The column was washed extensively with binding buffer, followed by binding buffer containing an additional 50 mM l-arginine and 50 mM l-glutamine and binding buffer with 30 mM imidazole. *T. castaneum* JH receptor complex was eluted from the IMAC column using binding buffer containing 400 mM imidazole. Fractions eluting from the Ni-IMAC column and containing *T. castaneum* JH receptor complex were pooled and incubated with anti-FLAG antibody affinity resin previously equilibrated with PBS containing 10 μM methoprene. This resin was prepared by coupling M2 anti-FLAG monoclonal antibody, a product of the American Type Culture Collection 4E11 cell line listed as 4.00E+11 or HB-9259, to Mini Leak Low agarose resin 2.9 mmol groups per liter (Kementec; catalog no.: 1011L) according to the manufacturer's instructions. The column with bound protein was extensively washed with equilibration buffer before eluting with 0.4 mg/ml FLAG peptide in equilibration buffer.

High losses of purified *T. castaneum* JH receptor complex were incurred using centrifugal membrane-based concentrators. To overcome this issue, purified *T. castaneum* JH receptor complex was concentrated using anion exchange chromatography. Briefly, *T. castaneum* JH receptor complex eluting from the anti-FLAG affinity column was loaded onto a 1-ml HiTRAP Q HP column (GE Lifesciences) equilibrated in 50 mM Tris, pH 8.5, containing 2 mM MgCl_2_, 10 mM DTT, and 10 μM methoprene, with elution *via* a sharp 0 to 1.0 M NaCl linear gradient.

SDS-PAGE using 4 to 12% gradient Bis–Tris NuPAGE gels and MES electrophoresis buffer systems (Thermo Fisher Scientific) under reducing conditions was followed by immunoblotting. Antihistidine horseradish peroxidase conjugate (Sigma; A7058) and anti-FLAG M2 horseradish peroxidase conjugate (Sigma; A8592) were used to monitor purification of the *T. castaneum* JH receptor complex. Immunoblots were developed using the Supersignal Western Pico Plus chemiluminescent substrate (Thermo Fisher Scientific), and images were captured using a Versadoc imaging system (Bio-Rad). The molecular weight was estimated using SeeBlue2 molecular weight standards (Thermo Fisher Scientific).

### Anti-FLAG pull downs

For quantitative proteomics identification of methoprene-dependent JHR interaction partners and phosphorylation sites, recombinant FLAG-tagged *T. castaneum* JH receptor complexes were expressed in insect cells, and anti-FLAG pull downs were prepared. Briefly, baculovirus-infected *Sf9*-III cells expressing *T. castaneum* JH receptor were incubated in 50 ml Sf900-III SFM with or without 1 μM methoprene for 70 h plus 4 μM methoprene for another 2 h. The cells were harvested 72 h postinfection, washed twice in ice-cold PBS containing 5 μM methoprene by centrifugation (1000*g*, 10 min, 4 °C), frozen in liquid nitrogen, and then stored at −70 °C.

To prepare FLAG pull downs, cell pellets of 1 ml were resuspended in 4 ml ice-cold lysis buffer with or without methoprene (25 mM Tris, pH 7.4, 150 mM NaCl, 2 mM KCl, 1 mM PMSF, 1 mM sodium tartrate dibasic, 1 mM sodium molybdate, 1× complete protease inhibitors (Roche), 1× phosphatase inhibitor cocktail, and ±10 μM methoprene). Lysates were prepared by sonication on ice (microtip; 40 Hz, 6× 15 s cycles with 15 s rest between each cycle) and centrifuged to remove insoluble material and cell debris (Beckman JA 25.50 rotor; 18,000 rpm, 30 min, 4 °C). Soluble protein was mixed with 400 μl of a 50% slurry of washed anti-FLAG M2 affinity resin (prepared as described previously) and incubated at 4 °C on a rotating wheel for 1 h. Anti-FLAG beads were centrifuged (2000 rpm, 2 min, 4 °C) to collect unbound supernatant (flow-through) and extensively washed in 4× 10 ml ice-cold lysis buffer ± 10 μM methoprene. FLAG-tagged *T. castaneum* JH receptor complexes were eluted by stepwise addition of 0.2 ml (E1), 0.4 ml (E2), and 0.4 ml (E3) of lysis buffer containing 0.4 mg/ml FLAG peptide ± 10 μM methoprene. The lysate, starting material, flow-through, and elution fractions were analyzed by Coomassie-stained SDS-PAGE and Western blot (anti-His and anti-FLAG antibodies) for quality control prior to MS.

### Native MS

Native mass spectra were acquired with a high-resolution Synapt G2Si mass spectrometer (Waters) equipped with a nano-ESI source in positive ion mode. Protein samples were exchanged into 200 mM ammonium acetate (pH 7.4) buffer using Zeba Spin desalting columns (Thermo Fisher Scientific), and native MS was performed in static infusion mode using coated fused-silica PicoTips (New Objective). To guarantee the ionization effect of the sample solution, the capillary voltage was maintained at 1800 V. The temperature and flow rate of the desolvation gas were maintained at 250 °C and 0.6 l/min, respectively. The nebulizer gas pressure was 6.5 bar. To achieve a favorable desolvation effect and to increase the sensitivity, the trap collision energy was set at 90 eV. Quadrupole frequencies, radiofrequency amplitudes, and transfer times were adjusted to achieve best ion transmission efficiency. Native MS data were acquired at 1 Hz across a mass/charge range of 500 to 10,000 Da, and MS raw data were processed using the Protein Metrics Intact Mass parsimonious charge deconvolution algorithm (78).

### Intact mass analysis by LC–MS

The accurate mass of purified recombinant proteins was determined by denaturing LC–MS. Protein samples were spiked with formic acid (FA) to a final concentration of 0.1% (v/v) and separated by reverse-phase LC on an UltiMate 3000 RSLCnano system (Thermo Fisher Scientific) fitted with a 50 × 4.6 mm, 5 μM particle size, 300 Å pore size PLRP-S column (Agilent). Proteins were eluted at a flow rate of 250 μl/min by applying a linear 30 min gradient from 0 to 80% solvent B (mobile phase A: 0.1% [v/v] FA; mobile phase B: 90% [v/v] acetonitrile/0.1% [v/v] FA) using an Apollo II electron spray ion source coupled to a micrOTOF-QII mass spectrometer (Bruker). The instrument was calibrated in positive ion mode using ESI-L Low Concentration Tuning Mix (Agilent), and LC–MS raw data were processed and deconvoluted using the MaxEnt algorithm as part of Bruker Compass Data Analysis, version 4.3.

### Sample preparation for MS and UPLC–MS/MS phosphoproteomics analysis

For peptide sequencing, affinity MS, and quantitative phosphoproteomics analyses, purified recombinant JHR complexes or anti-FLAG pull downs were digested using a single-pot solid phase–enhanced sample preparation method (79). Protein samples (50–100 μg) were reduced with 5 mM TCEP (Sigma) for 15 min, alkylated for 30 min with 50 mM iodoacetamide (Sigma), and digested with 1 μg trypsin gold (Promega) for 16 h at 37 °C. For quantitative proteomics the peptide solutions were desalted using C18 MacroSpin columns (Nest Group) prior to on-column stable isotope dimethyl labeling of differentially treated samples using light (CH_2_O) or heavy (CD_2_O) formaldehyde (Sigma), as previously described (80). Light (+28 Da) and heavy (+32 Da) labeled peptide samples were mixed 1:1 in 1% FA and subjected to phosphopeptide enrichment using TopTip TiO_2_ microspin columns according to the protocol provided by the manufacturer (Glygen). TiO_2_-bound or TiO_2_-unbound fractions were resuspended in 10 μl 0.1% FA, and 3-μl aliquots were separated in duplicate using a two-column chromatography setup comprising a PepMap100 C18 20 mm × 75 μm trap and a PepMap C18 500 mm × 75 μm analytical column (Thermo Fisher Scientific). Samples were concentrated on the trap column at 5 μl/min for 5 min and infused into an Orbitrap Fusion Lumos Tribrid mass spectrometer (Thermo Fisher Scientific) at 300 nl/min running 120 min gradients from 99% buffer A (0.1% FA) to 40% buffer B (99% acetonitrile and 0.1% FA) on a Dionex Ultimate 3000 UPLC system (Thermo Fisher Scientific). The Lumos mass spectrometer was operated in a data-dependent mode, automatically switching between the acquisition of a single Orbitrap MS scan (resolution of 120,000) every 3 s and the top-20 multiply charged precursors selected for EThcD fragmentation (maximum fill time of 100 ms; automatic gain control of 5 × 10^4^ with a resolution of 30,000 for Orbitrap MS/MS scans).

### Mass spectra database searching

MS/MS database searches for the characterization of purified JHR complexes and identification of peptide sequences were carried out using Mascot, version 2.3 (Matrix Science). Scaffold, version 4.4.3 (Proteome Software) was used to probabilistically validate Mascot (phospho)peptide identifications using the PeptideProphet algorithm (Table S1 and Fig. S4) (81). The shotgun proteomics data have been deposited to the ProteomeXchange Consortium (http://proteomecentral.proteomexchange.org) *via* the PRIDE partner repository with the dataset identifier PXD028394 (82). Relative quantification of associated proteins and confidently localized phosphorylation sites was accomplished using MaxQuant (version 1.5.3.1) (83). Searches were performed against a custom protein database containing *S. frugiperda* Swiss-Prot and TrEMBL sequences, recombinant FLAG-*T. castaneum* methoprene-tolerant, His_6_-TcTAI, FLAG-AaTAI, and His_6_-AaMET proteins, as well as common proteomics contaminants (28,148 entries). High-resolution MS/MS data were searched with trypsin cleavage specificity allowing two miscleavage events, carbamidomethylation of cysteine (+57 Da) set as a fixed modification and oxidation of methionine (+16 Da), acetylation (+42 Da) of protein N termini, and phosphorylation (+80 Da) of serine, threonine, or tyrosine as variable modifications. Stable isotope-labeled samples were searched allowing for additional variable light (L; +28 Da) or heavy (H; +32 Da) dimethyl labels on the peptide N termini and lysine residues. The precursor mass tolerance was set to 20 ppm for the first search and 10 ppm for the main search with a maximum FDR of 1.0% set for protein and peptide identifications. To enhance the identification of peptides between samples, the match between runs option was enabled with the precursor match window set to 2 min and an alignment window of 10 min in addition to the requantification module. Quantitative LC–MS/MS experiments were conducted using two biological replicates with five technical replicates for affinity-purified fractions and three technical replicates for phosphopeptide-enriched fractions. A robust permutation test was used to analyze MaxQuant data and evaluate statistically significant differences in the relative abundance of *T. castaneum* JH receptor-associated proteins or phosphopeptides (84), and only phosphorylation sites with MaxQuant ΔScore >50 and localization probability >50% were considered. The quantitative (phospho)proteomics data have been deposited to the ProteomeXchange Consortium (http://proteomecentral.proteomexchange.org) *via* the PRIDE partner repository with the dataset identifier PXD028599.

### SEC–MALLS

FLAG-*T. castaneum* methoprene-tolerant and His_6_-TcTAI were coexpressed and purified as described previously. Purified proteins at ∼5 mg/ml were subjected to size-exclusion chromatography using a Superdex 200 10/300 column (GE Healthcare) at a flow rate of 0.5 ml/min in 50 mM Tris, 350 mM NaCl, 10 μM methoprene, 2 mM MgCl_2_, 100 μM TCEP, pH 8.0, with UV monitoring at 280, 254, and 215 nm. Fractions were collected in aliquots of 0.5 ml. Light scattering and concentration data were collected by in-line miniDawn TREOS MALLS detector and Optilab T-rEX refractometer (Wyatt Technology), respectively. The weight-average molecular weights were calculated using the intensity of the scattered light in combination with the change in refractive index, whereas the protein concentration at the detector was determined by the change in refractive index. UV, MALLS, and differential refractive index data were collected using the ASTRA software (Wyatt Technology). Molecular weight determinations were also done with the ASTRA software according to the Zimmerman model using the standard dn/dc value of 0.1852 ml/g for proteins (85).

### EMSA

Three types of JHRE DNA sequences previously shown to bind MET–TAI complexes from *T. castaneum* and *A. aegypti* and/or mediate MET–TAI dependent transcriptional activation were employed as double-stranded DNA probes to demonstrate binding of our recombinant JHR complexes. The *k*-JHRE sequence TCGCCTC**CACGTG**CCGTTTCA originates from the immediate JH-response gene *Kr-h1* of *T. castaneum* (50). The E-box–like palindrome (in bold type) is recognized by certain bHLH–PAS proteins. A mutated sequence TCGCCTCACATGTCCGTTTCA lacking this core motif served as a negative control. A second element CCATCCCA**CACGCG**AAGACGATAAAACCA whose core contains an imperfect palindrome (bold) was identified as an enhancer of the JH-inducible *ET* gene of *A. aegypti* (39). We refer to this sequence as *ET*-JHRE. Finally, we used a probe GCCG**CACGTG**TCGTTGG that again contained the CACGTG palindrome and which was defined as a consensus binding site (called MFBS1) for the *A. aegypti* MET–TAI complex in an unbiased oligonucleotide screen (24). We added a “T” to the 5′ end of each of the single-stranded oligonucleotides, so that radioactive labeling could be achieved after annealing of the complementary strands using a fill-in reaction with the large Klenow fragment of DNA polymerase and α-[^32^P]dATP.

The assay was performed with the purified *T. castaneum* JH receptor and AaJHR proteins as previously described for a MET–TAI complex from *A. aegypti* (24) with minor modifications. When the JHR complexes were to be dephosphorylated, the protein was treated with 40 U/μl of λPP (New England Biolabs) for 1 h at 4 °C prior to the assay. The EMSA was set up with 250 ng of a JHR protein complex in reactions of 20 μl containing 20 mM Tris–HCl (pH 7.8), 50 mM NaCl, 5 mM MgCl_2_, 5 mM DTT, 10 μM JH III (Sigma), 10% glycerol, 500 ng/μl bovine serum albumin (BSA), 50 ng/μl of poly(dA-dT), and any unlabeled DNA competitor. After 10 min at room temperature, a [^32^P]-labeled double-stranded oligonucleotide probe (20 fmol) was added, and the reactions were incubated for a further 20 min. The protein–DNA complexes were then resolved by electrophoresis on a 6% polyacrylamide gel in Tris-borate-EDTA (pH 8.3), after which the gel was fixed, dried, and processed using the Typhoon Phosphoimager 9410. Band intensities were quantified from the raw phosphoimager data as integrated density using the ImageJ software (the National Institutes of Health).

### Site-directed mutagenesis of *T. castaneum* methoprene-tolerant

The overlap extension PCR method (86) was employed to generate *T. castaneum* methoprene-tolerant variants lacking the putative C-terminal region bipartite NLS by substituting with alanine the pairs of basic residues on either end of the motif: K455A;R456A (NLS^1^) and K471A;R472A (NLS^2^), respectively (Fig. 8*A*). The same method was used to replace the potentially phosphorylated T189 and S191 residues identified in the PAS-A domain with alanine or aspartic acid (variants 2A and 2D, respectively). In all cases, PCR with primers introducing the mutations was performed on a template encoding the entire WT *T. castaneum* methoprene-tolerant in a *pK-Myc-C2* plasmid, and each mutated product was verified by sequencing. For *T. castaneum* methoprene-tolerant variants 7A^1^, 7A^2^, and 7D with overlapping sets of seven serine/threonine residues near the C terminus simultaneously mutated to alanine or aspartic (Fig. 8*A*), the coding DNA was custom synthesized (GenScript).

### [^3^H]-JH III binding assay

Purified recombinant JHR proteins were assayed for the ability to bind [^3^H]-JH III (PerkinElmer) essentially as described (23). However, instead of dextran-coated charcoal being used to distinguish between free and protein-bound [^3^H]-JH III, the hormone–protein complex was captured on GF/C glass fiber discs (Whatman) as routinely employed in our laboratory to monitor [^3^H]-ponasterone A binding to EcRs (51). Assays were performed in PEG 20,000 coated 6 × 50 mm glass tubes. Binding buffer contained 20 mM Tris (pH 7.8), 5 mM Mg acetate, 1 mM EDTA disodium salt, and 1 mM DTT. [^3^H]-JH III was freshly prepared in aqueous solution by evaporating [^3^H]-JH III stored in hexane and replacing the solvent with cold binding buffer to give approximately 20,000 dpm in 15 μl per assay. JHR proteins were diluted in cold binding buffer containing 0.1% BSA to give a final concentration of 0.3 μM. Assays were incubated at room temperature for 30 min. Each assay was applied to a Whatman GF/C glass fiber filter disc and allowed to adsorb for 30 s before transferring to a glass sinter assembly under vacuum and washing with 15 ml ice-cold binding buffer (without BSA). Each filter was transferred to a scintillation vial, and 7 ml InstaGel Plus (Packard TriCarb) scintillation fluid was added. One protein-free control sample did not undergo the washing procedure to record the [^3^H]-JH III input. Vials were counted for 3 min using a PerkinElmer TRI-CARB 4910 TR liquid scintillation counter. To assess the JH III binding capacity of *T. castaneum* methoprene-tolerant variants mutated in the potential phosphorylation sites, the coding DNA sequences were cloned behind a T7 RNA polymerase promoter and a Myc epitope in the *pK-Myc-C2* vector. The Myc-tagged *T. castaneum* methoprene-tolerant proteins were expressed by *in vitro* transcription and translation using the TnT Quick T7-coupled rabbit reticulocyte lysate system (Promega) (23). The resulting proteins were then tested for [^3^H]-JH III binding as described previously.

### Cell transfection, immunocytochemistry, and microscopy image analysis

To assess the subcellular localization of *T. castaneum* methoprene-tolerant lacking its predicted NLS or phosphorylation sites, the control and mutated Myc-*T. castaneum* methoprene-tolerant constructs in *pK-Myc-C2* were transiently transfected to the HEK293 cell line, where the proteins were expressed under the cytomegalovirus promoter promoter. The cells were grown in Dulbecco's modified Eagle's medium (Invitrogen) supplemented with 10% FBS and were transfected with 1 μg DNA per well in a 6-well plate using the FuGENE HD reagent (Promega). Transfected cells were grown for 36 h on microscope cover slips submersed in the culture wells, after which they were fixed with 4% formaldehyde in PBS, permeabilized with 0.2% Triton X-100, and stained with the 9E10 mouse monoclonal antibody (1:500; Invitrogen) against the Myc epitope, followed by incubation with a secondary antibody coupled with Cy3 (Jackson ImmunoResearch). The samples were mounted in the Vectashield medium (Vector Laboratories), containing 4′,6-diamidino-2-phenylindole for DNA counterstaining. When the effect of a *T. castaneum* methoprene-tolerant ligand was to be tested, 0.5 μM methoprene in dimethyl sulfoxide was added to the medium 1 h prior to cell fixation. Images were captured using the FluoView FV1000 laser scanning microscope (Olympus) as individual single slices. The relative distribution of Myc-*T. castaneum* methoprene-tolerant was estimated from the confocal slices of constant thickness by measuring signal intensities within the nuclear and cytoplasmic areas of the transfected HEK293 cells. The data were recorded using the ImageJ software (the National Institutes of Health) as mean gray values, and ratios of nuclear-to-cytoplasmic signal intensity were calculated for a number of individual cells from single confocal slices of constant thickness. To examine *T. castaneum* methoprene-tolerant localization in cells derived from Tc, Myc-*T. castaneum* methoprene-tolerant was expressed from the pIEx-4 vector (Novagen) in the TcA cell line (87). TcA cells were grown at 27 °C in the Ex-CELL 420 SFM (Sigma) supplemented with 10% FBS, transiently transfected, and subjected to immunostaining as described previously for the HEK293 cell line.

## Data availability

The MS data can be obtained from the PRIDE repository. The Mascot dataset and the associated LC–MS/MS raw data and database search results are accessible under project identifier PXD028394 (LC–MS/MS analyses of purified and recombinant *T. castaneum* JH receptor complexes). The MaxQuant dataset and the associated isotopically labeled phosphopeptide-enriched LC–MS/MS raw data are accessible under project identifier PXD028599 (quantitative proteomics analysis of affinity-purified *T. castaneum* JH receptor complexes).

## Dedication

This communication is dedicated to Thomas Gordon Wilson 1946 to 2017.

## Supporting information

This article contains supporting information.

## Conflict of interest

The authors declare that they have no conflicts of interest with the contents of this article.
