# Supplementary material for: Purification of an insect juvenile hormone receptor complex enables insights into its post-translational phosphorylation
Source: J Biol Chem. 2021 Nov 7;297(6):101387. doi: 10.1016/j.jbc.2021.101387 (PMC8683598; doi:10.1016/j.jbc.2021.101387)
Supplement: Supplemental Figures S1–S8 [file mmc1.pdf]

SUPPORTING INFORMATION

Purification of an insect juvenile hormone receptor complex enables insights into its post-translational phosphorylation

Marek Jindra[1,#,*], William J. McKinstry[2,#], Thomas Nebl[2,#], Lenka Bittova[1], Bin Ren[2], Jan Shaw[3], Tram Phan[2], Louis Lu[2], Jason K. K. Low[4], Joel P. Mackay[4], Lindsay G. Sparrow[2], George O. Lovrecz[2], and Ronald J. Hill[3,4,*]

From the [1]Biology Center, Czech Academy of Sciences, Institute of Entomology, Ceske Budejovice 37005, Czech Republic, [2]CSIRO Manufacturing, Parkville, Victoria 3052, Australia, [3]CSIRO Health and Biosecurity, North Ryde, NSW 2113, Australia, [4]School of Life and Environmental Sciences, University of Sydney, NSW 2006, Australia

Running title: *Recombinant JH receptor proteins*

[#]These authors contributed equally to this work.

***To whom correspondence should be addressed:**
Biology Center, Czech Academy of Sciences, Branisovska 31, Ceske Budejovice 37005, Czech Republic; jindra@entu.cas.cz; Tel. +420-387775232
and
School of Life and Environmental Sciences, Building G08, Butlin Ave, The University of Sydney, NSW 2006, Australia; ronald.hill@sydney.edu.au; Tel. +61-414640424

MATERIAL INCLUDED

Figure S1

Figure S2

Figure S3

Figure S4

Figure S5

Figure S6

Figure S7

Figure S8

Table S1

Table S2

Table S3

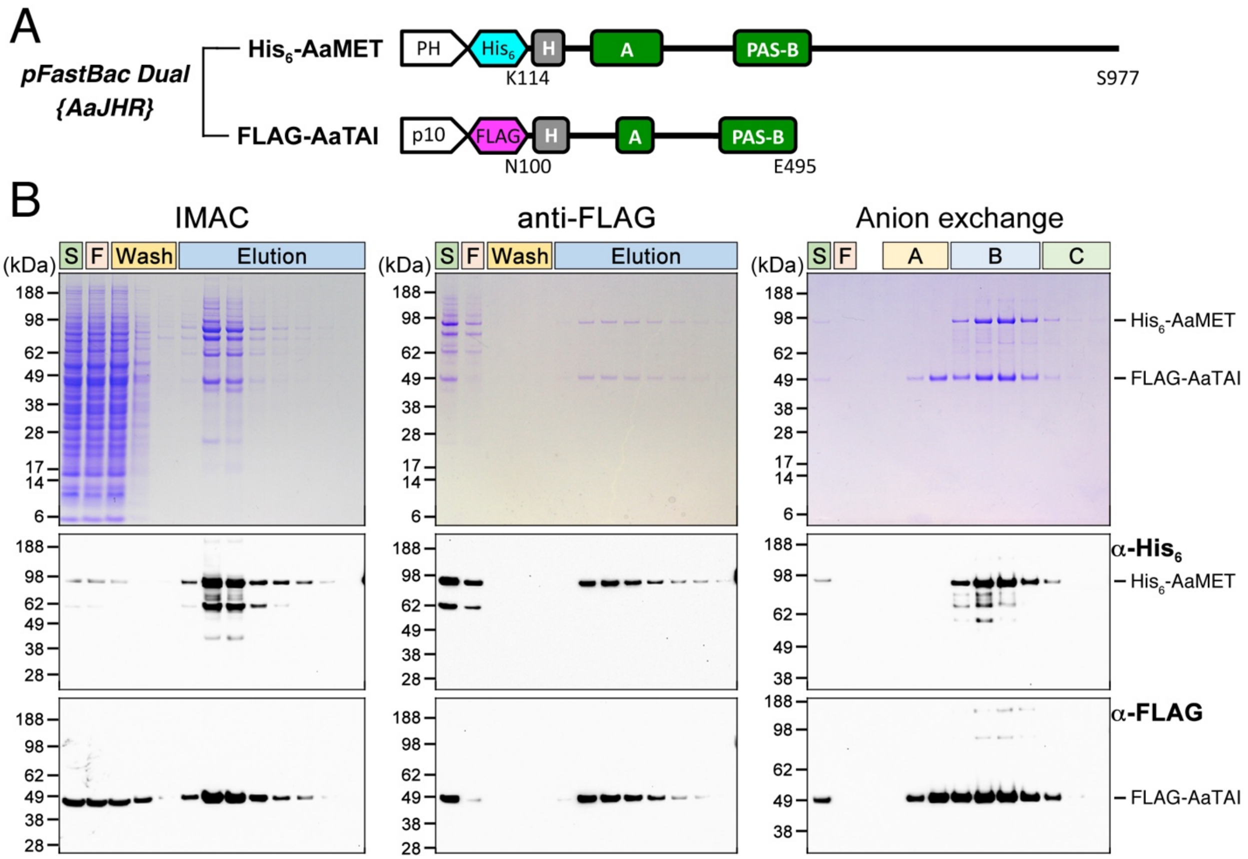

Figure S1. Expression and purification of the recombinant JHR proteins of *A. aegypti.* *A*, the interacting components of the AaJHR complex, the truncated AaMET and AaTAI proteins were co-expressed in *Sf9* cells from the baculoviral *pFastBac Dual* construct under the polyhedrin (PH) and p10 promoters, respectively. N-terminal affinity tags and the major functional domains are depicted. *B*, the AaJHR protein complex was purified from lysates of baculovirus infected *Sf9* cells in two affinity chromatography steps using immobilized Ni^{2+} (IMAC) and anti-FLAG antibody, respectively, followed by anion exchange chromatography, all in the presence of 10 μM methoprene. Top panels show Coomassie stained SDS-PAGE, below are immunoblots of the same samples developed with the indicated anti-tag antibodies. S, starting lysate; F, flow-through.

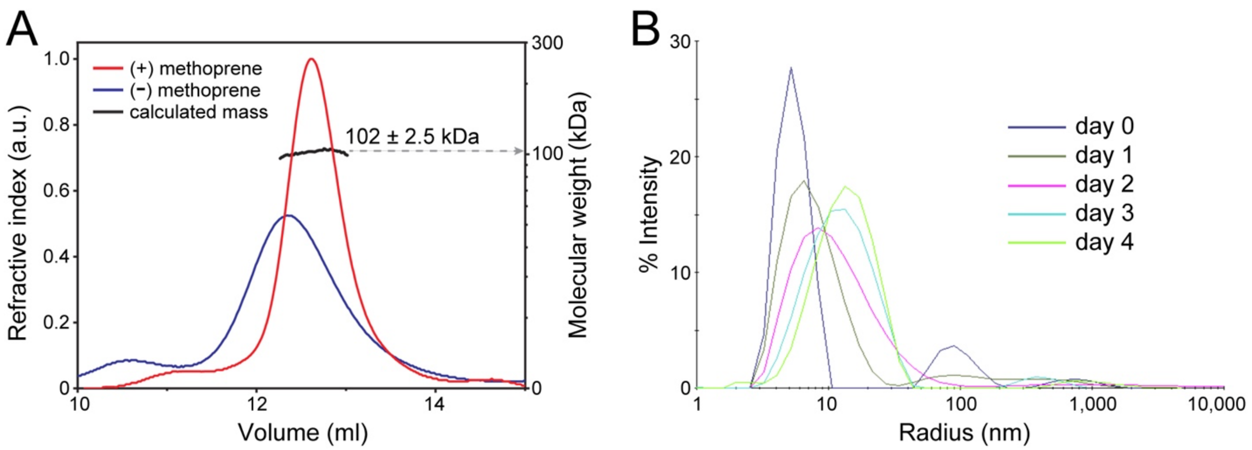

Figure S2. Aggregation of the TcJHR protein as a function of time and presence of methoprene. *A*, SEC-MALLS analysis of purified TcJHR that had been dialyzed against three changes of 4,000 volumes of 50 mM Tris, 350 mM NaCl, 2 mM $MgCl_2$, 100 µM TCEP, pH 8.0 buffer for 18 h at 3°C with (+, red trace) or without 10 µM methoprene (-, blue trace). *B*, dynamic light scattering was performed with a DynaPro Plate Reader (Wyatt). The aggregation status of the TcJHR protein was monitored at 0°C over a period of five days with 20 µl of protein (3.47 mg/ml in 50 mM Tris pH 8.0, 350 mM NaCl, 10 mM DTT, 2 mM $MgCl_2$, 10 µM methoprene). The protein kept on aggregating over time during this period, with the main peak radius increased from about 5.5 nm to 11.8 nm.

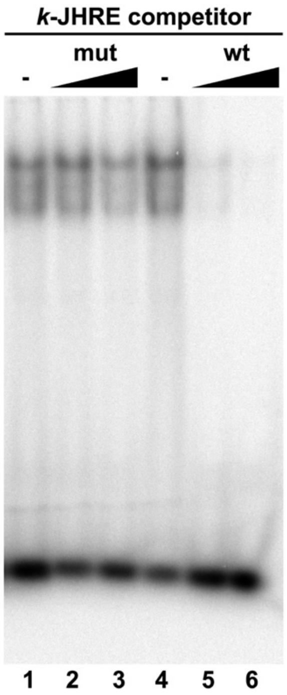

Figure S3. **Binding of the purified AaJHR protein complex to the *k*-JHRE DNA was inhibited by excess of the unlabeled specific JHRE probe**. The AaJHR protein complex was incubated with the radiolabeled *k*-JHRE DNA probe in the absence of unlabeled competitor (lanes 1 and 4) or the presence of 500- and 1000-fold excess of either mutated (mut, lanes 2 and 3) or wild-type (wt, lanes 5 and 6) versions of the unlabeled DNA competitor. JH III (10 μM) was added to all reactions.

Protein	Residue	Sequence	Score	ΔScore	Modifications	M_{obs}	Charge	M_{calc}	ΔPPM
FLAG-TcMET	T448	(R)VYGNVNENQDC(+57)STPT(+80)ENSPTKPYYK(L)	23.6	1.9	Carbamidomethyl (+57), Phospho (+80)	995.7587	3	2,984.25	0.16

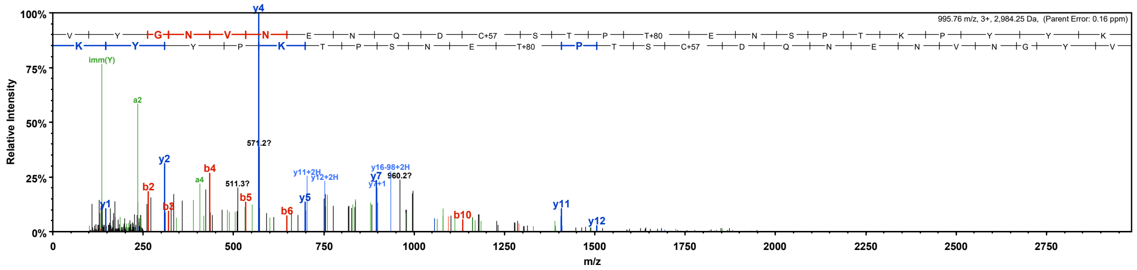

Protein	Residue	Sequence	Score	ΔScore	Modifications	M_{obs}	Charge	M_{calc}	ΔPPM
FLAG-TcMET	T448	(R)VYGNVNENQDC(+57)STPT(+80)ENSPTKPYYK(L)	20.1	1.8	Carbamidomethyl (+57), Phospho (+80)	995.7587	3	2,984.25	0.16

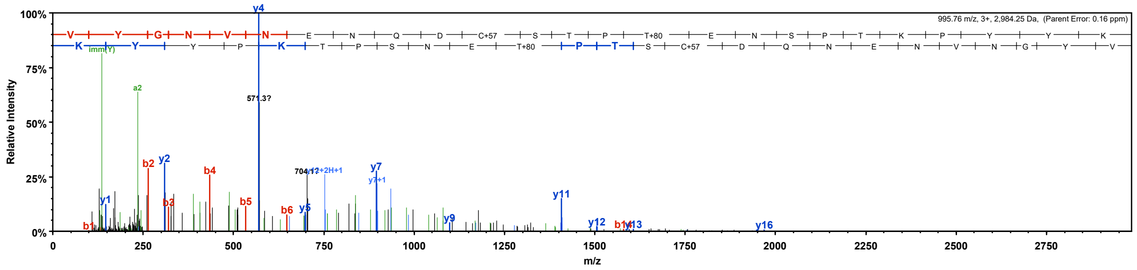

Protein	Residue	Sequence	Score	ΔScore	Modifications	M_{obs}	Charge	M_{calc}	ΔPPM
FLAG-TcMET	S451	(R)VYGNVNENQDC(+57)STPTENS(+80)PTKPYYK(L)	50.9	8.6	Carbamidomethyl (+57), Phospho (+80)	747.0691	4	2,984.25	-2.19

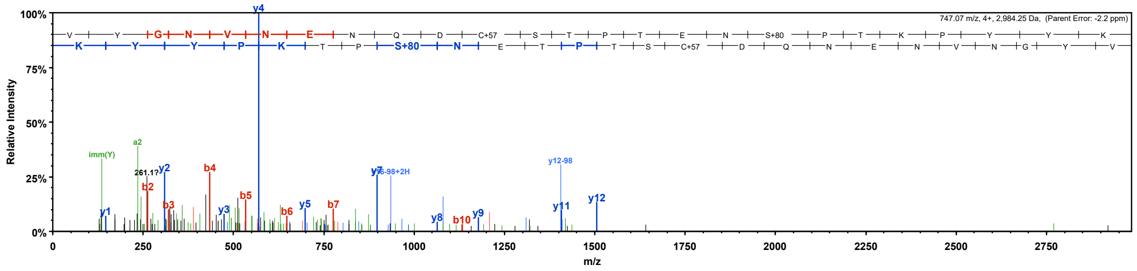

Protein	Residue	Sequence	Score	ΔScore	Modifications	M_{obs}	Charge	M_{calc}	ΔPPM
FLAG-TcMET	S451	(R)VYGNVNENQDC(+57)STPTENS(+80)PTKPYYK(L)	42.6	1.8	Carbamidomethyl (+57), Phospho (+80)	995.7609	3	2,984.26	2.40

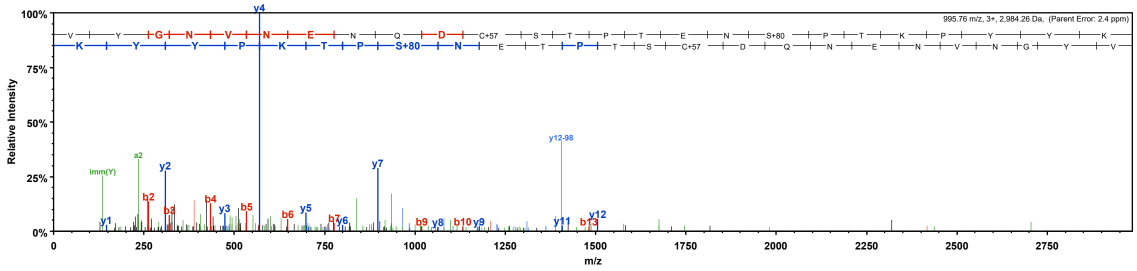

Protein	Residue	Sequence	Score	ΔScore	Modifications	M_{obs}	Charge	M_{calc}	ΔPPM
FLAG-TcMET	S451	(R)VYGNVNENQDC(+57)STPTENS(+80)PTKPYYK(L)	35.4	4.4	Carbamidomethyl (+57), Phospho (+80)	995.7587	3	2,984.25	0.16

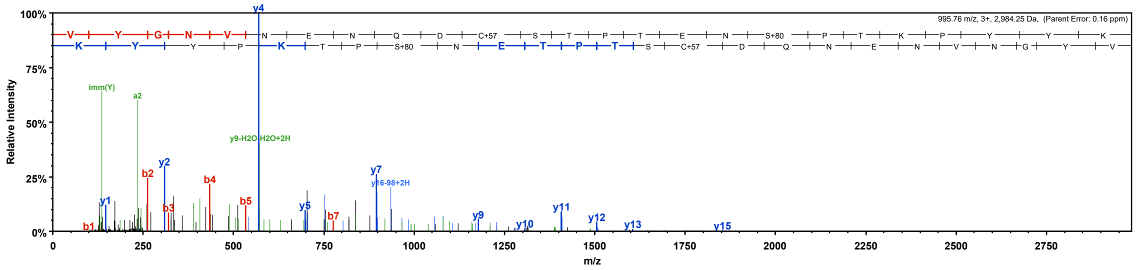

Protein	Residue	Sequence	Score	ΔScore	Modifications	M_{obs}	Charge	M_{calc}	ΔPPM
FLAG-TcMET	S451	(R)VYGNVNENQDC(+57)STPTENS(+80)PTKPYYK(L)	22.5	6.5	Carbamidomethyl (+57), Phospho (+80)	995.7582	3	2,984.25	-0.32

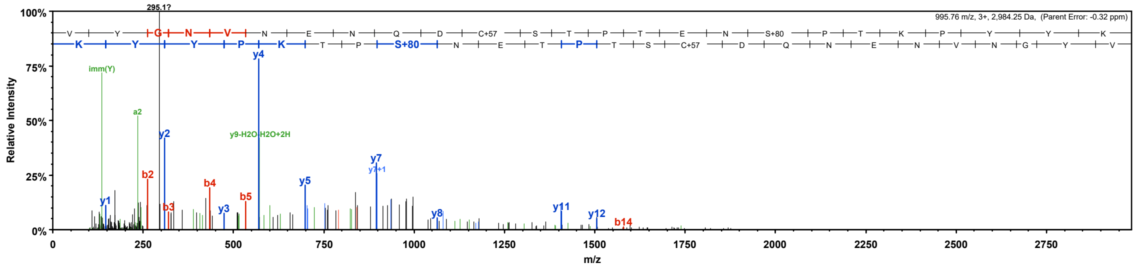

Protein	Residue	Sequence	Score	ΔScore	Modifications	M_{obs}	Charge	M_{calc}	ΔPPM
FLAG-TcMET	S469	(K)RPPS(+80)TELGTNIYTSSK(R)	74.2	7.9	Phospho (+80)	610.9562	3	1,829.85	-5.15

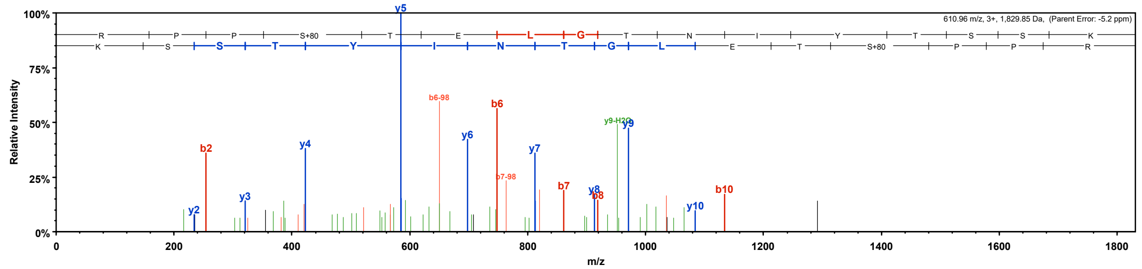

Protein	Residue	Sequence	Score	ΔScore	Modifications	M_{obs}	Charge	M_{calc}	ΔPPM
FLAG-TcMET	S469	(K)RPPS(+80)TELGTNIYTSSK(R)	51.5	7.7	Phospho (+80)	915.9351	2	1,829.86	-0.26

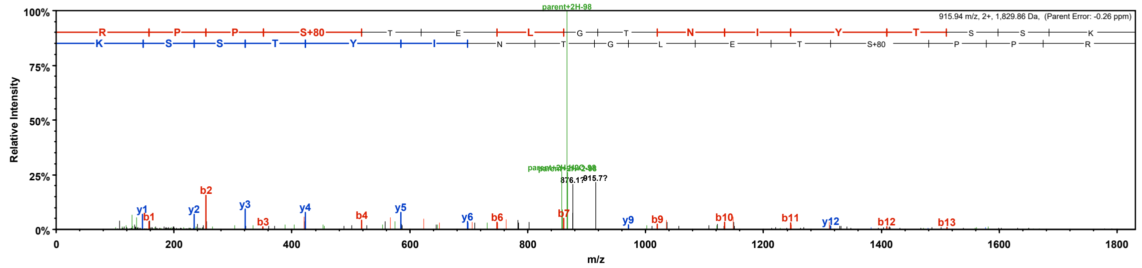

Protein	Residue	Sequence	Score	ΔScore	Modifications	M_{obs}	Charge	M_{calc}	ΔPPM
FLAG-TcMET	S469	(K)RPPS(+80)TELGTNIYTSSK(R)	25.9	0.9	Phospho (+80)	915.9351	2	1,829.86	-0.27

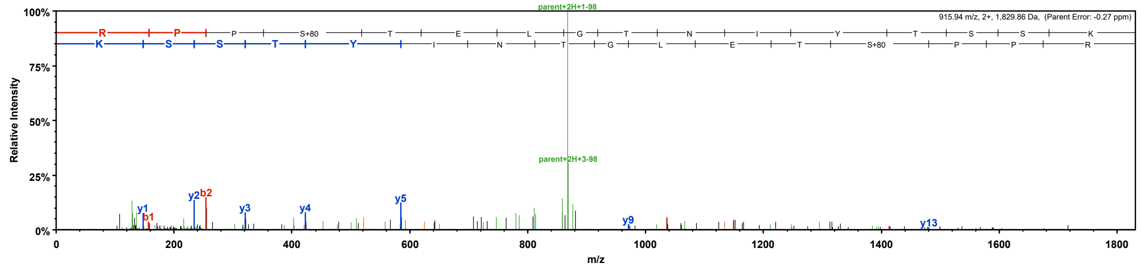

Protein	Residue	Sequence	Score	ΔScore	Modifications	M_{obs}	Charge	M_{calc}	ΔPPM
FLAG-TcMET	S469	(K)RPPS(+80)TELGTNIYTSSK(R)	20.9	0.8	Phospho (+80)	915.9352	2	1,829.86	-0.19

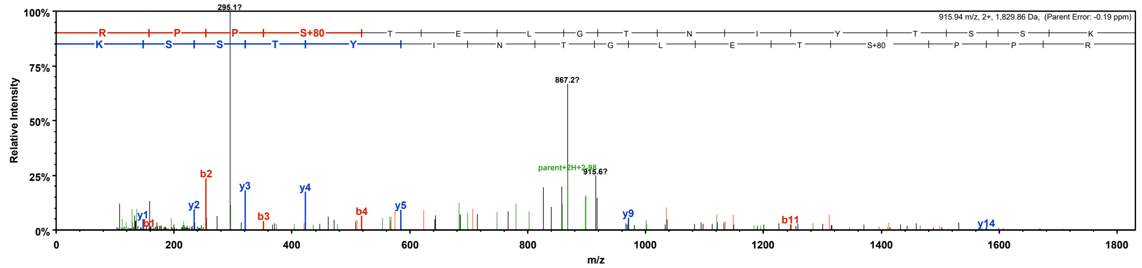

Protein	Residue	Sequence	Score	ΔScore	Modifications	M_{obs}	Charge	M_{calc}	ΔPPM
FLAG-TcMET	S470	(K)RPPST(+80)ELGTNIYTSSK(R)	71.3	1.5	Phospho (+80)	610.9558	3	1,829.85	-5.89

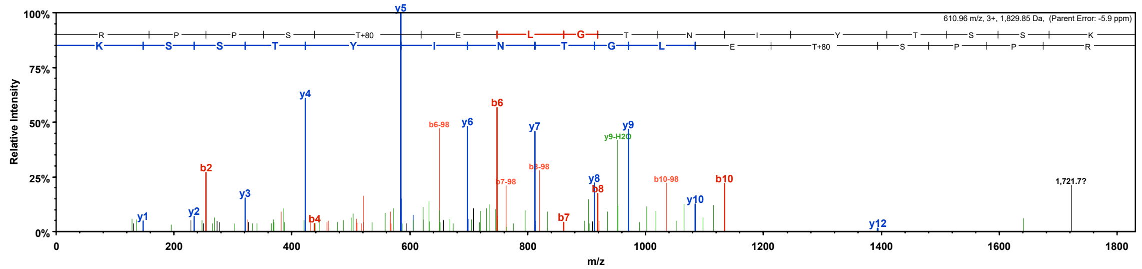

Protein	Residue	Sequence	Score	ΔScore	Modifications	M_{obs}	Charge	M_{calc}	ΔPPM
FLAG-TcMET	S470	(K)RPPST(+80)ELGTNIYTSSK(R)	30.8	1.2	Phospho (+80)	915.9351	2	1,829.86	-0.27

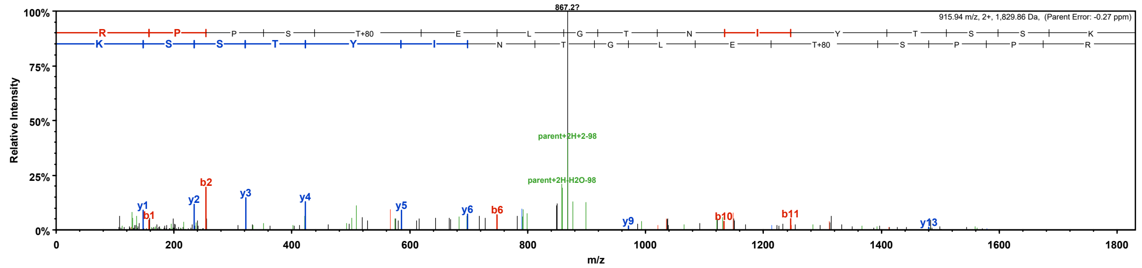

Protein	Residue	Sequence	Score	ΔScore	Modifications	M_{obs}	Charge	M_{calc}	ΔPPM
FLAG-TcMET	S470	(K)RPPST(+80)ELGTNIYTSSK(R)	29.8	4.7	Phospho (+80)	915.9352	2	1,829.86	-0.19

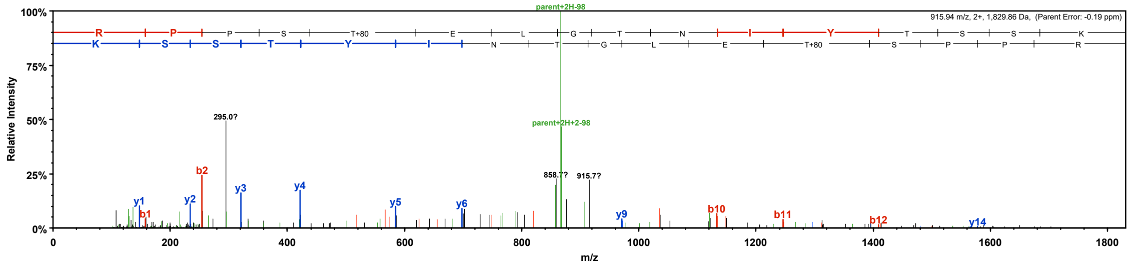

Protein	Residue	Sequence	Score	ΔScore	Modifications	M_{obs}	Charge	M_{calc}	ΔPPM
FLAG-TcMET	S486	(R)QRTS(+80)PQLSPMSSLPPYPNR(T)	32.6	1.1	Phospho (+80)	746.0241	3	2,235.05	-0.21

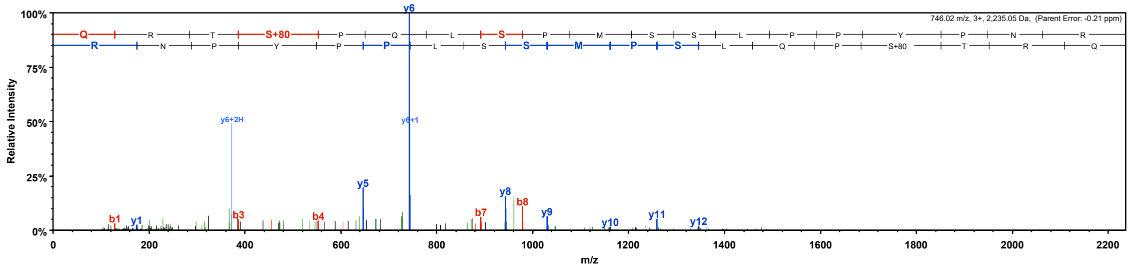

Protein	Residue	Sequence	Score	ΔScore	Modifications	M_{obs}	Charge	M_{calc}	ΔPPM
FLAG-TcMET	S490	(R)TSPQLS(+80)PMSSLPPYPNR(T)	79.2	34.7	Phospho (+80)	976.4528	2	1,950.89	-0.10

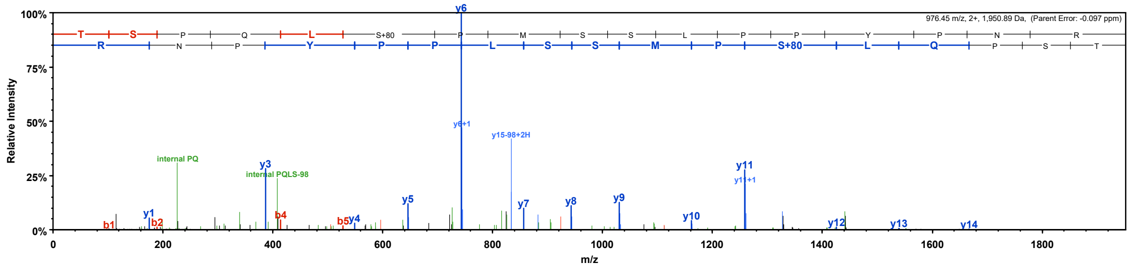

Protein	Residue	Sequence	Score	ΔScore	Modifications	M_{obs}	Charge	M_{calc}	ΔPPM
FLAG-TcMET	S490	(R)TSPQLS(+80)PMSSLPPYPNR(T)	64.4	16.6	Phospho (+80)	976.4524	2	1,950.89	-0.46

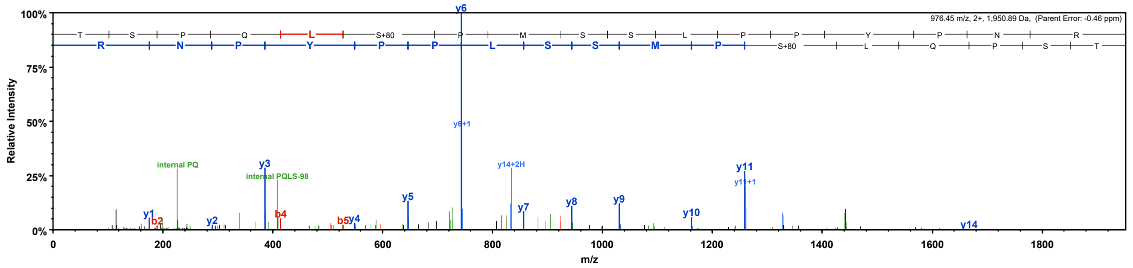

Protein	Residue	Sequence	Score	ΔScore	Modifications	M_{obs}	Charge	M_{calc}	ΔPPM
FLAG-TcMET	S490	(R)TSPQLS(+80)PMSSLPPYPNR(T)	43.1	3.4	Phospho (+80)	976.4526	2	1,950.89	-0.32

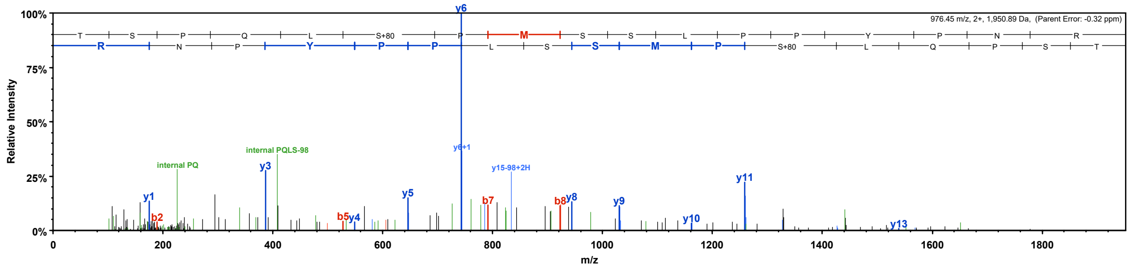

Protein	Residue	Sequence	Score	ΔScore	Modifications	M_{obs}	Charge	M_{calc}	ΔPPM
FLAG-TcMET	S490	(R)TSPQLS(+80)PMSSLPPYPNR(T)	38.6	11.9	Phospho (+80)	976.4526	2	1,950.89	-0.32

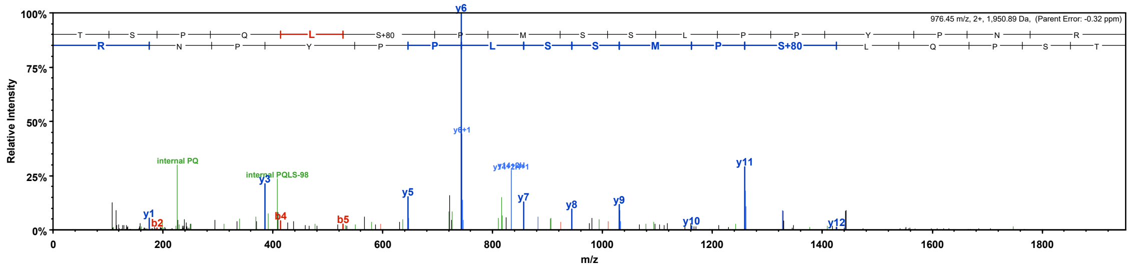

Protein	Residue	Sequence	Score	ΔScore	Modifications	M_{obs}	Charge	M_{calc}	ΔPPM
FLAG-TcMET	S490	(R)TSPQLS(+80)PM(+16)SSLPPYPNR(T)	30.2	3.0	Phospho (+80), Oxidation (+16)	656.6338	3	1,966.88	-3.34

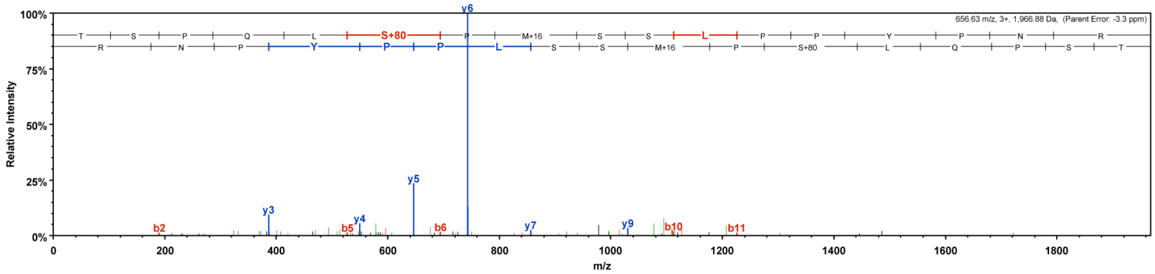

Protein	Residue	Sequence	Score	ΔScore	Modifications	M_{obs}	Charge	M_{calc}	ΔPPM
FLAG-TcMET	S490	(R)TSPQLS(+80)PMSSLPPYPNR(T)	29.2	13.2	Phospho (+80)	976.4528	2	1,950.89	-0.10

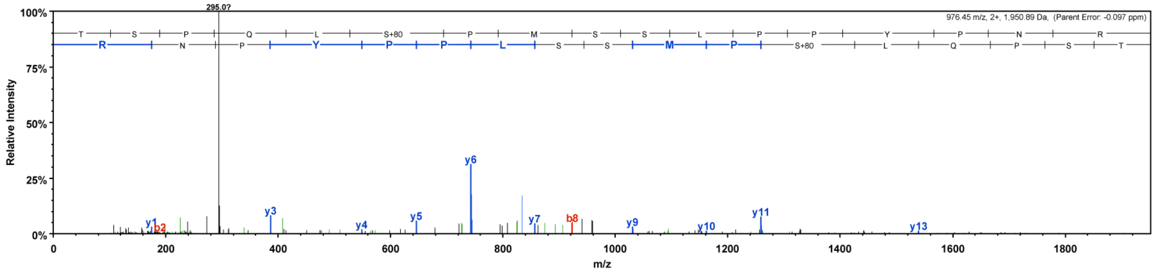

Protein	Residue	Sequence	Score	ΔScore	Modifications	M_{obs}	Charge	M_{calc}	ΔPPM
FLAG-TcMET	S493+S494	(R)QRTSPQLSPM(+16)S(+80)S(+80)LPPYPNR(T)	42.1	4.1	Oxidation (+16), Phospho (+80), Phospho (+80)	778.0154	3	2,331.02	5.27

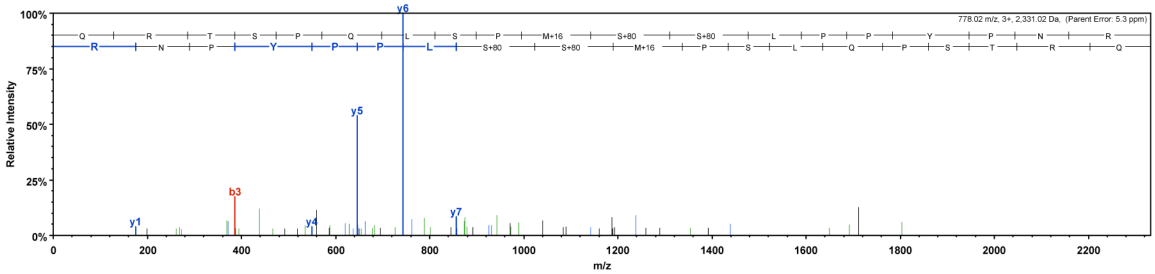

Protein	Residue	Sequence	Score	ΔScore	Modifications	M_{obs}	Charge	M_{calc}	ΔPPM
6xHis-TcTAI	S25	(R)KKS(+80)ETKPQAQINK(C)	37.7	1.3	Phospho (+80)	395.7105	4	2,331.02	-0.16

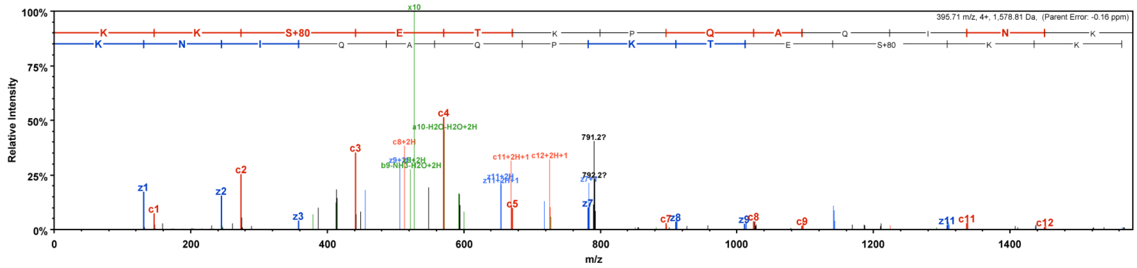

Figure S4. Annotated MS/MS evidence spectra of the purified, recombinant TcJHR complex based on shotgun proteomics. Confident phosphopeptide identifications are based on Mascot database searches, as shown in Table 1. Major singly or doubly charged (+2H) b-ions and y-ions are highlighted in blue and red, respectively. Matching precursor ions (parent), immonium ions (imm), neutral loss of phosphate (-98), neutral loss of water (-H_2O), neutral loss of ammonium (-NH_3), and internal fragment ions (internal) are highlighted in green. Column headers: Protein, name of protein construct; Residue, S/T phosphorylation site; Sequence, phosphopeptide sequence with S(+80) or T(+80) highlighting phosphorylation sites; M(+16), oxidized methionine; C(+57), carbamidomethylated cysteine; Score, Mascot score; ΔScore, Mascot delta score; M_{obs}, observed precursor ion mass; M_{calc}, calculated monoisotopic mass of protonated peptide ion; ΔPPM, mass error in parts per million.

Raw File	Scan	Method	Score	m/z
171207_TomN_A2	18118	FTMS; CID	58.37	579.81

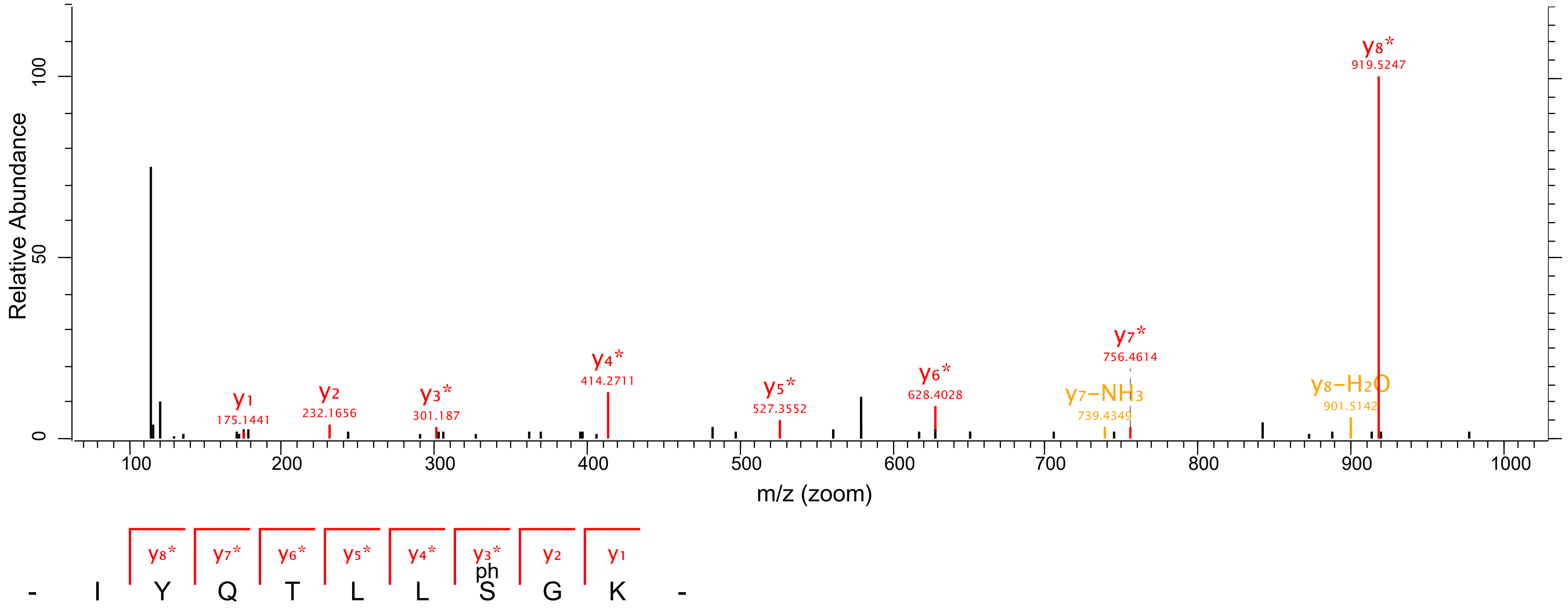

Protein	Accession	Phosphosite
FLAG-TcMET	A6MUT7	S86

Raw File	Scan	Method	Score	m/z
171207_TomN_A3	18136	FTMS; CID	60.52	579.81

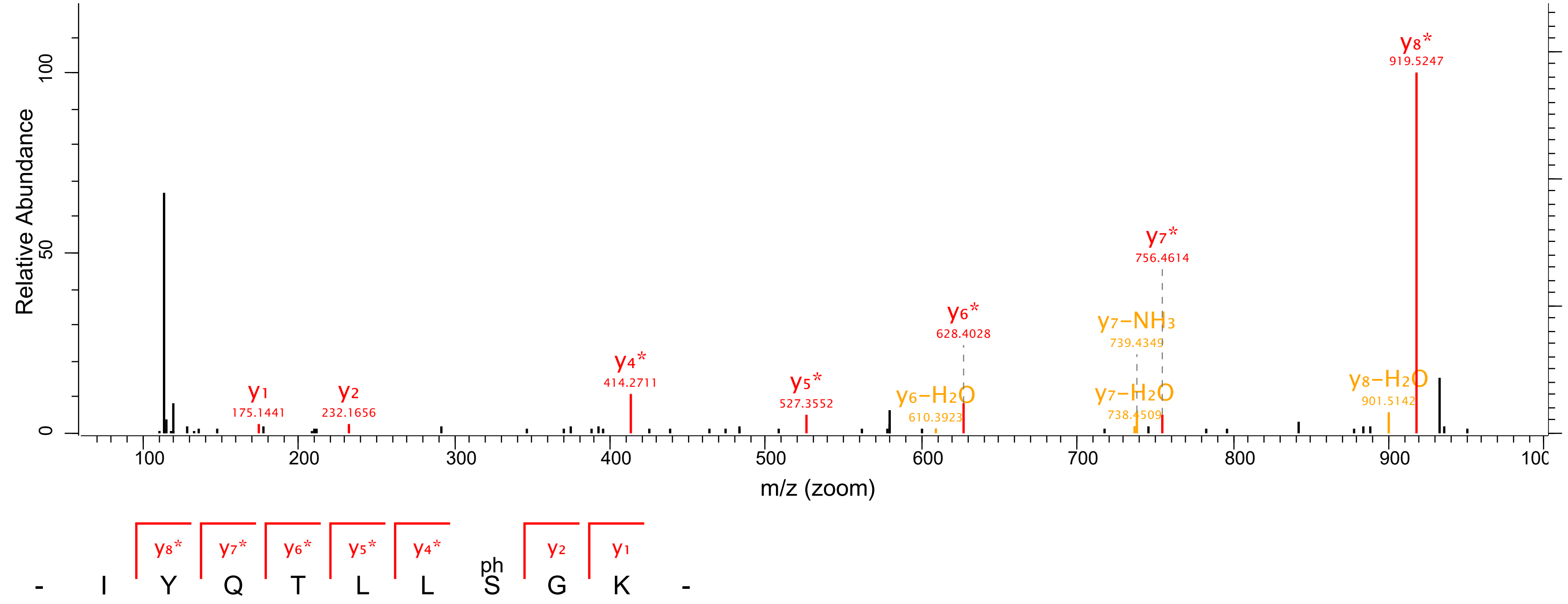

Protein	Accession	Phosphosite
FLAG-TcMET	A6MUT7	S86

Raw File	Scan	Method	Score	m/z
171207_TomN_A2	13375	FTMS; CID	149.96	629.79

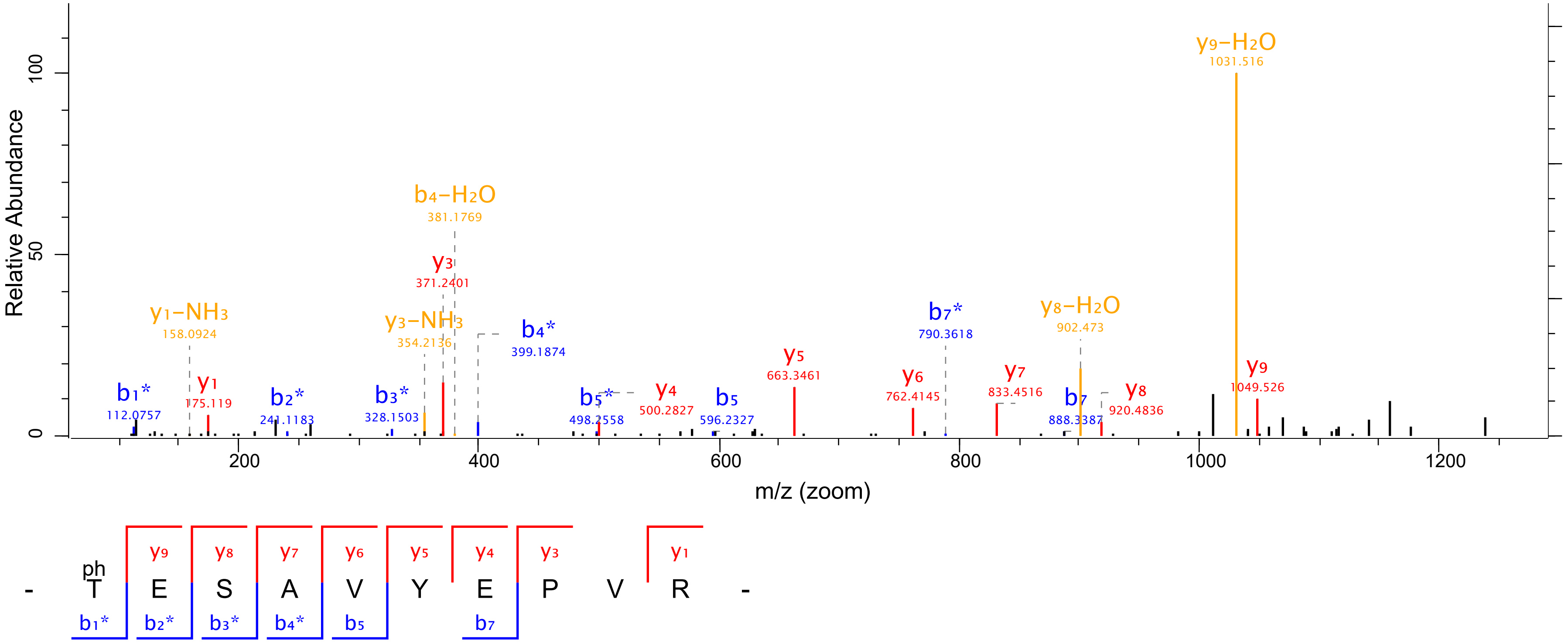

Protein	Accession	Phosphosite
FLAG-TcMET	A6MUT7	T189

Raw File	Scan	Method	Score	m/z
171207_TomN_A1	13309	FTMS; CID	220.5	632.81

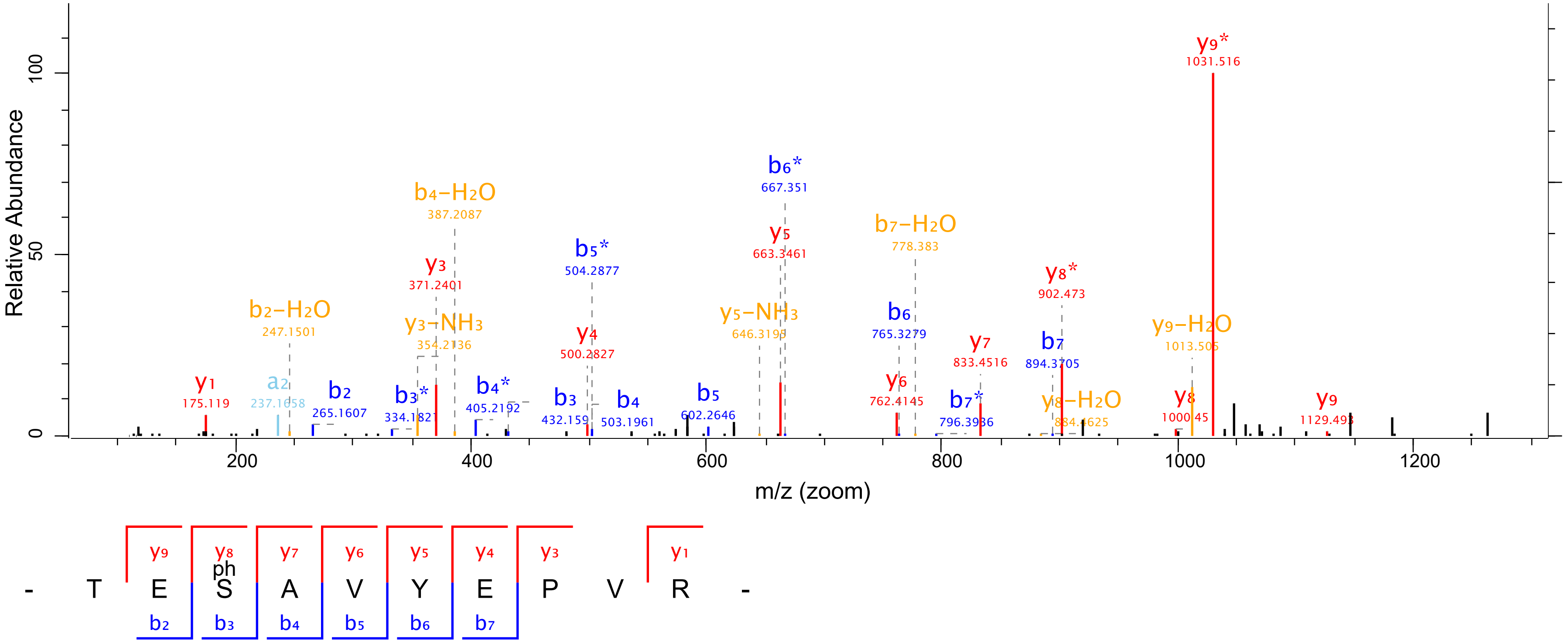

Protein	Accession	Phosphosite
FLAG-TcMET	A6MUT7	T191

Raw File	Scan	Method	Score	m/z
171207_TomN_A1	9407	FTMS; CID	53.17	632.81

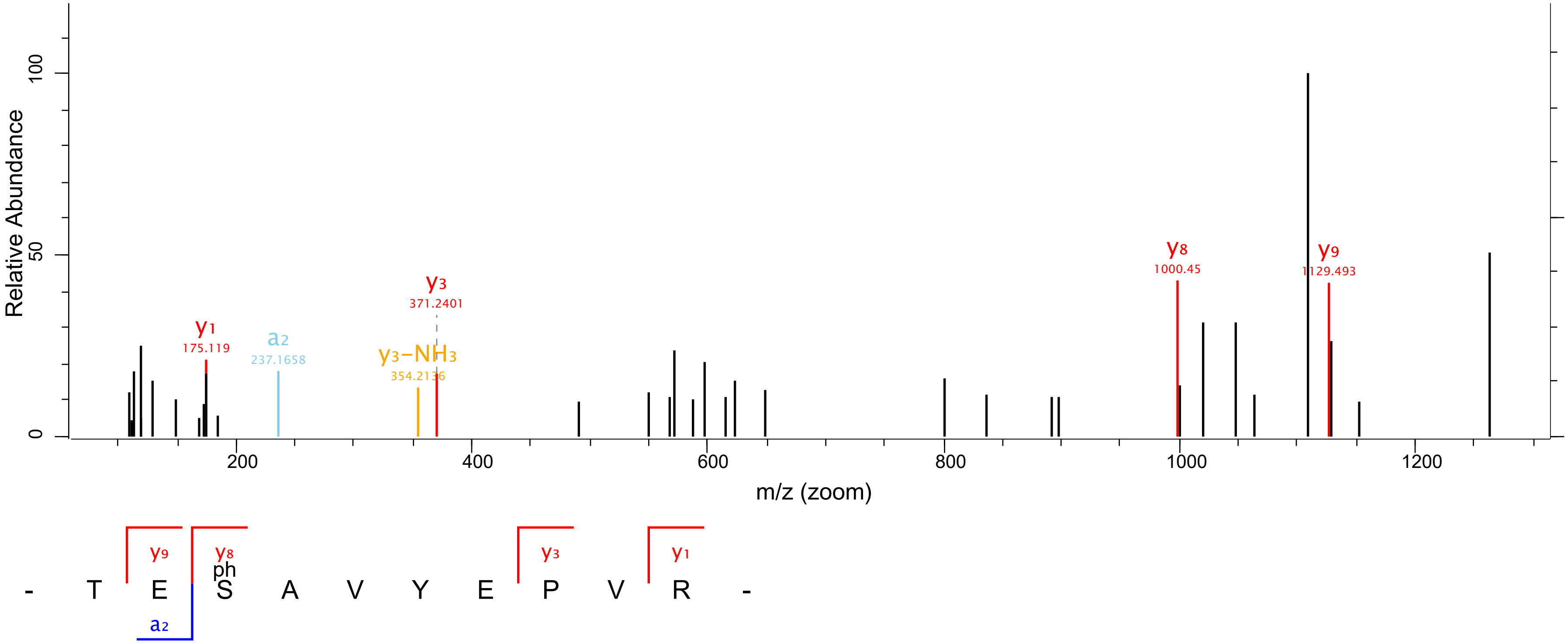

Protein	Accession	Phosphosite
FLAG-TcMET	A6MUT7	T191

Raw File	Scan	Method	Score	m/z
171017_TomN_A2_6uL	24527	ITMS; CID	90.13	1030.16

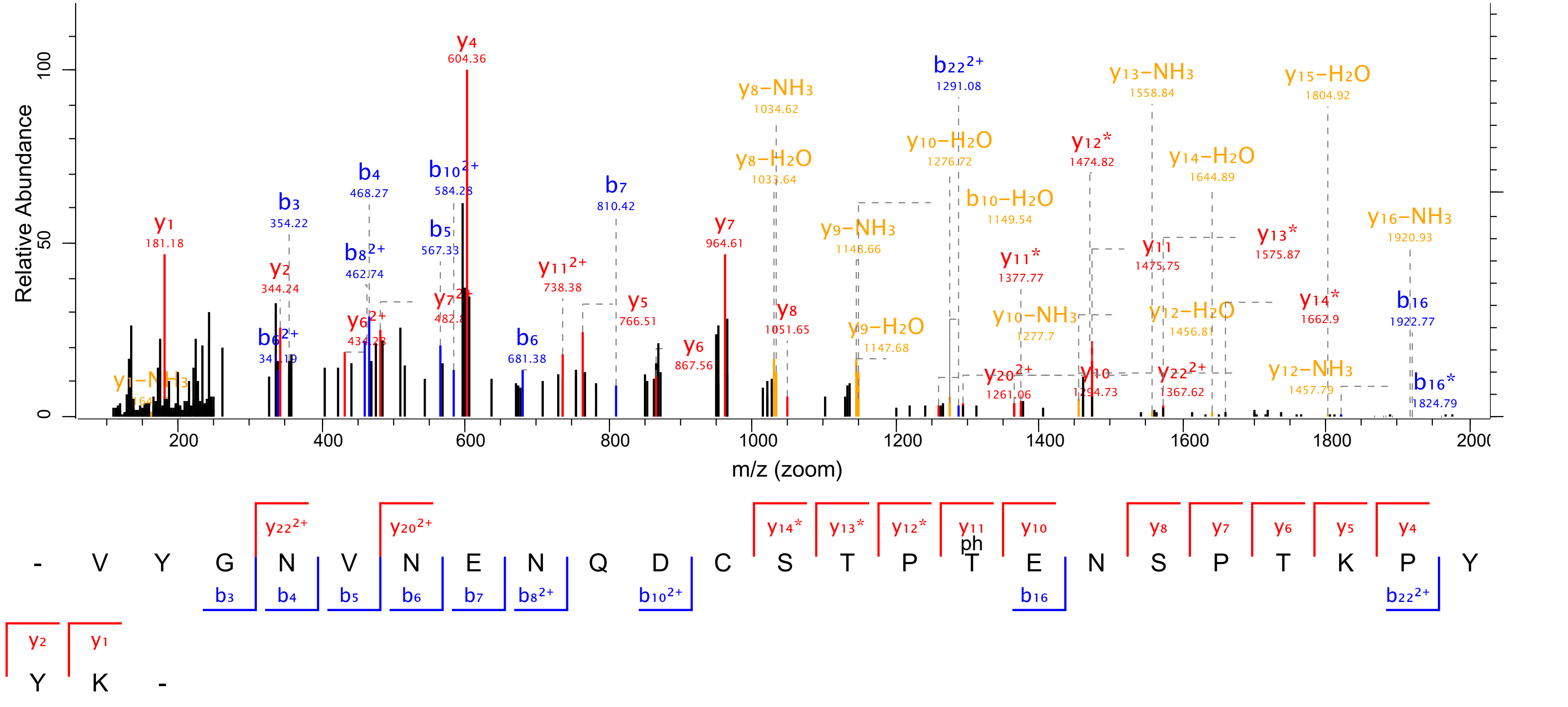

Protein	Accession	Phosphosite
FLAG-TcMET	A6MUT7	T438

Raw File	Scan	Method	Score	m/z
171017_TomN_B3_6uL	22283	ITMS; CID	106.44	1024.12

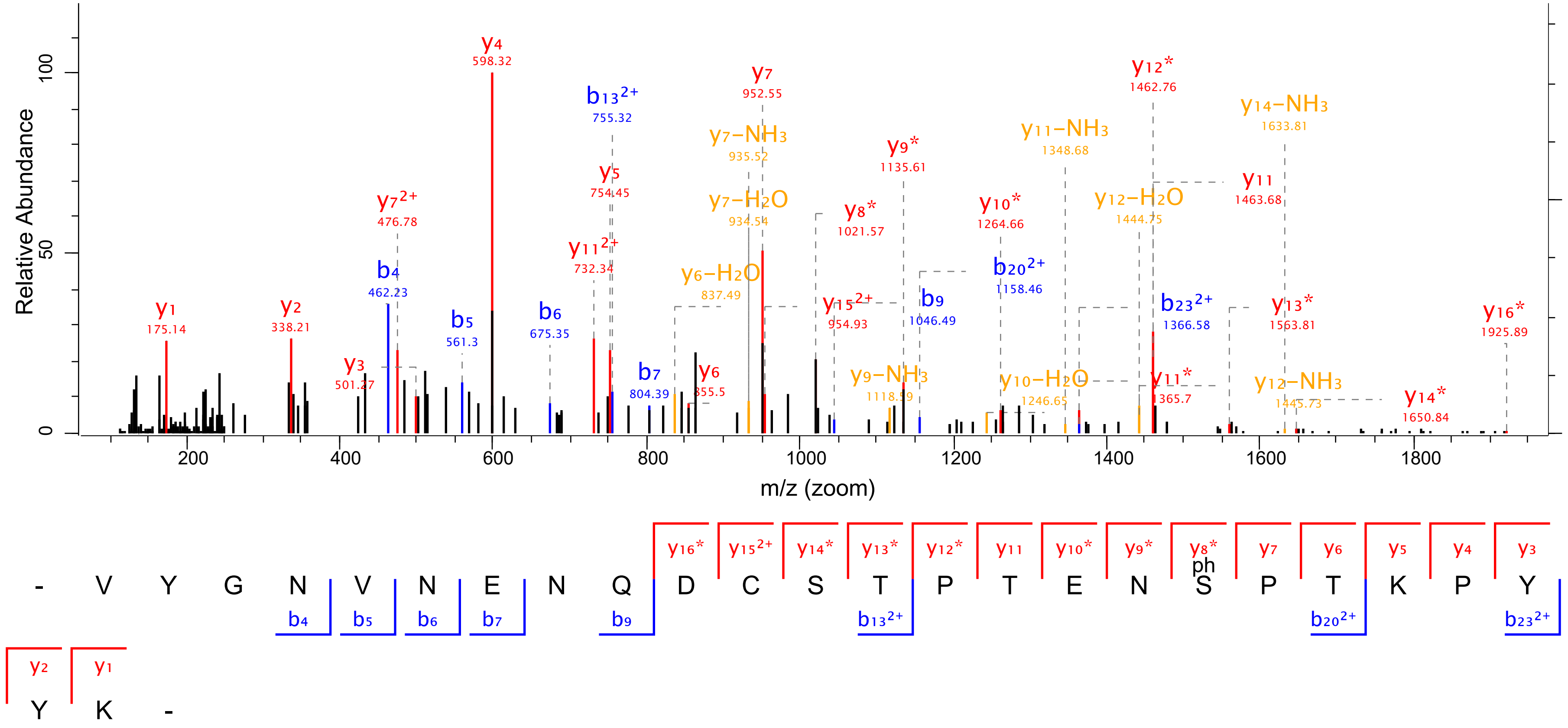

Protein	Accession	Phosphosite
FLAG-TcMET	A6MUT7	S441

Raw File	Scan	Method	Score	m/z
171207_TomN_A1	13533	FTMS; CID	248.46	943.97

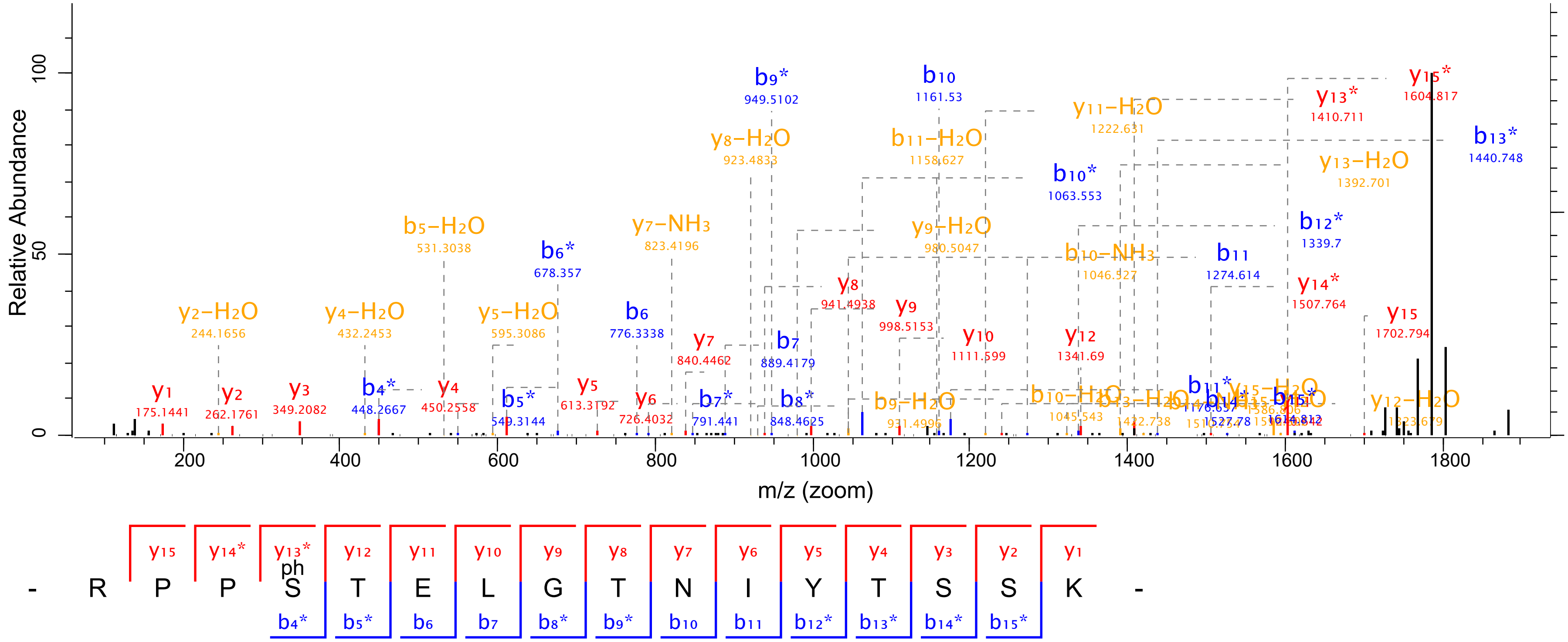

Protein	Accession	Phosphosite
FLAG-TcMET	A6MUT7	S459

Raw File	Scan	Method	Score	m/z
171207_TomN_A3	13654	FTMS; CID	216.16	944.47

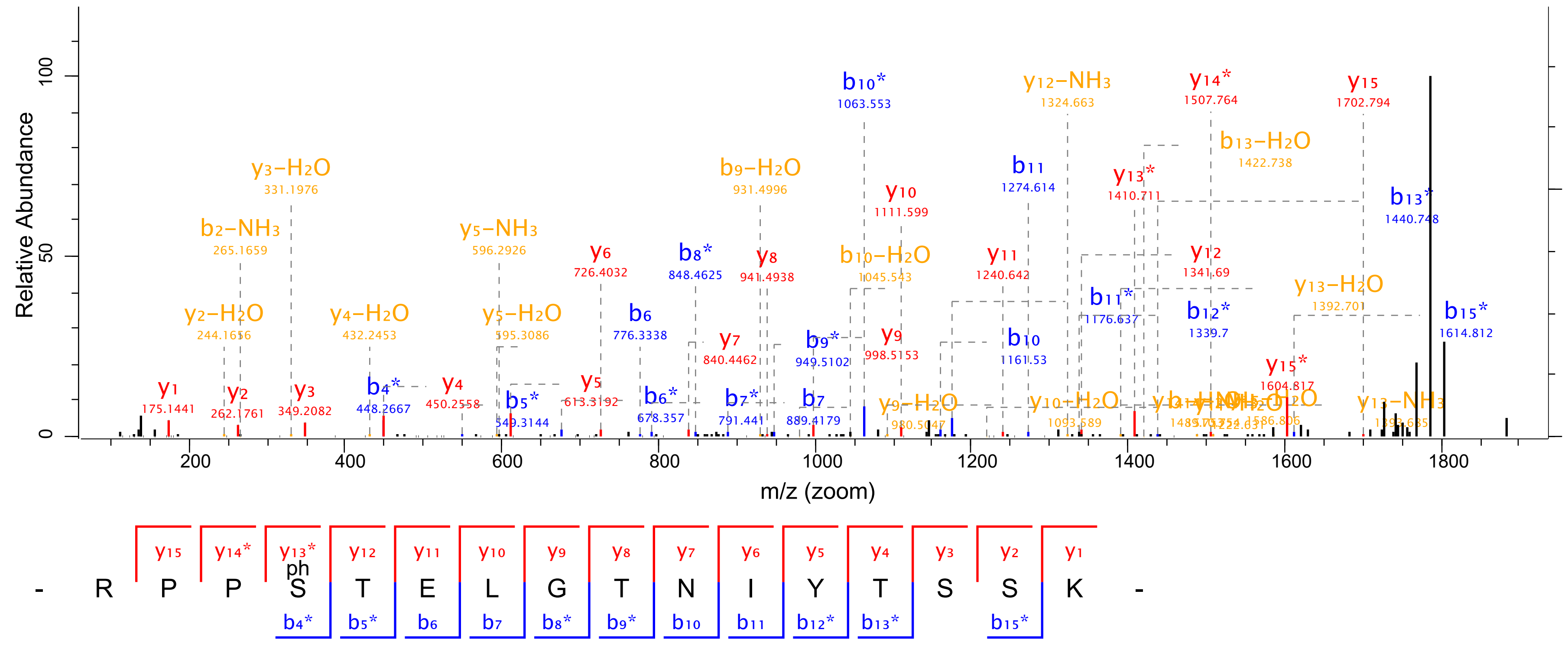

Protein	Accession	Phosphosite
FLAG-TcMET	A6MUT7	S459

Raw File	Scan	Method	Score	m/z
171207_TomN_B3	13589	FTMS; CID	189.87	944.47

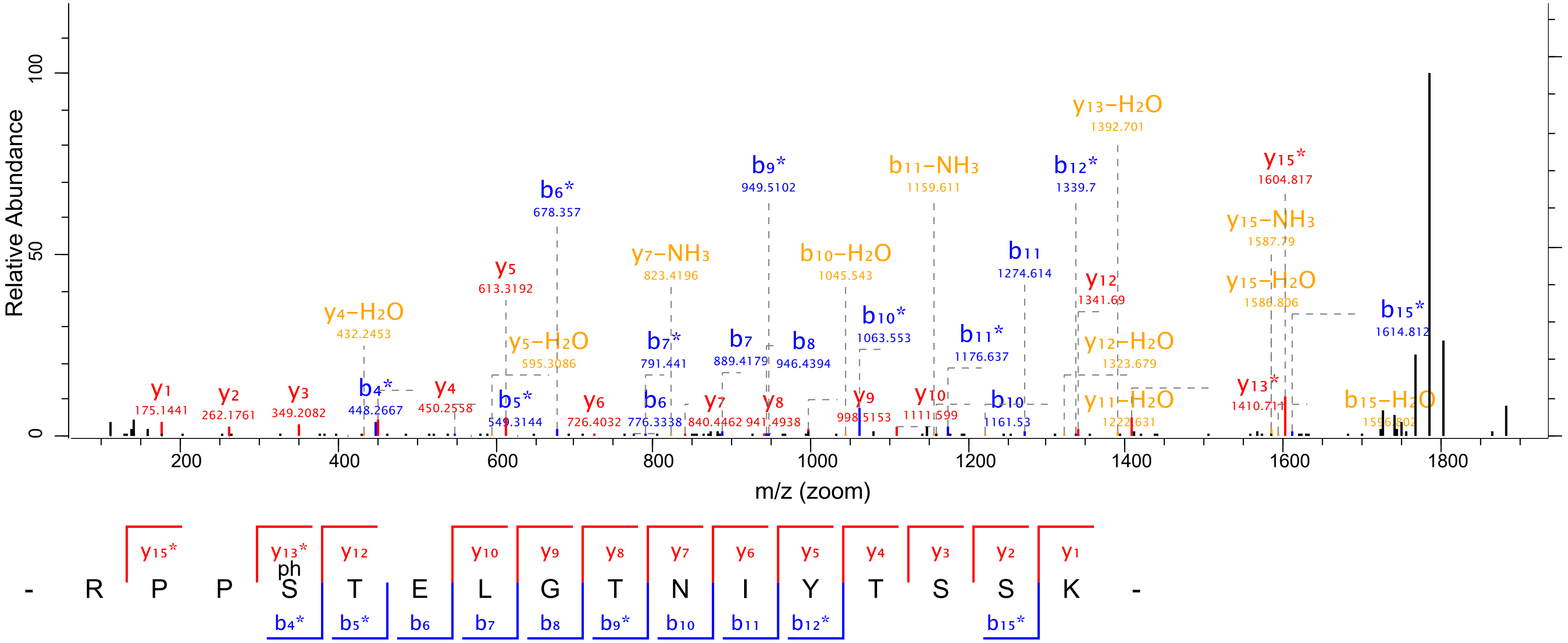

Protein	Accession	Phosphosite
FLAG-TcMET	A6MUT7	S459

Raw File	Scan	Method	Score	m/z
171017_TomN_A2_6uL	26266	ITMS; CID	113.47	944.47

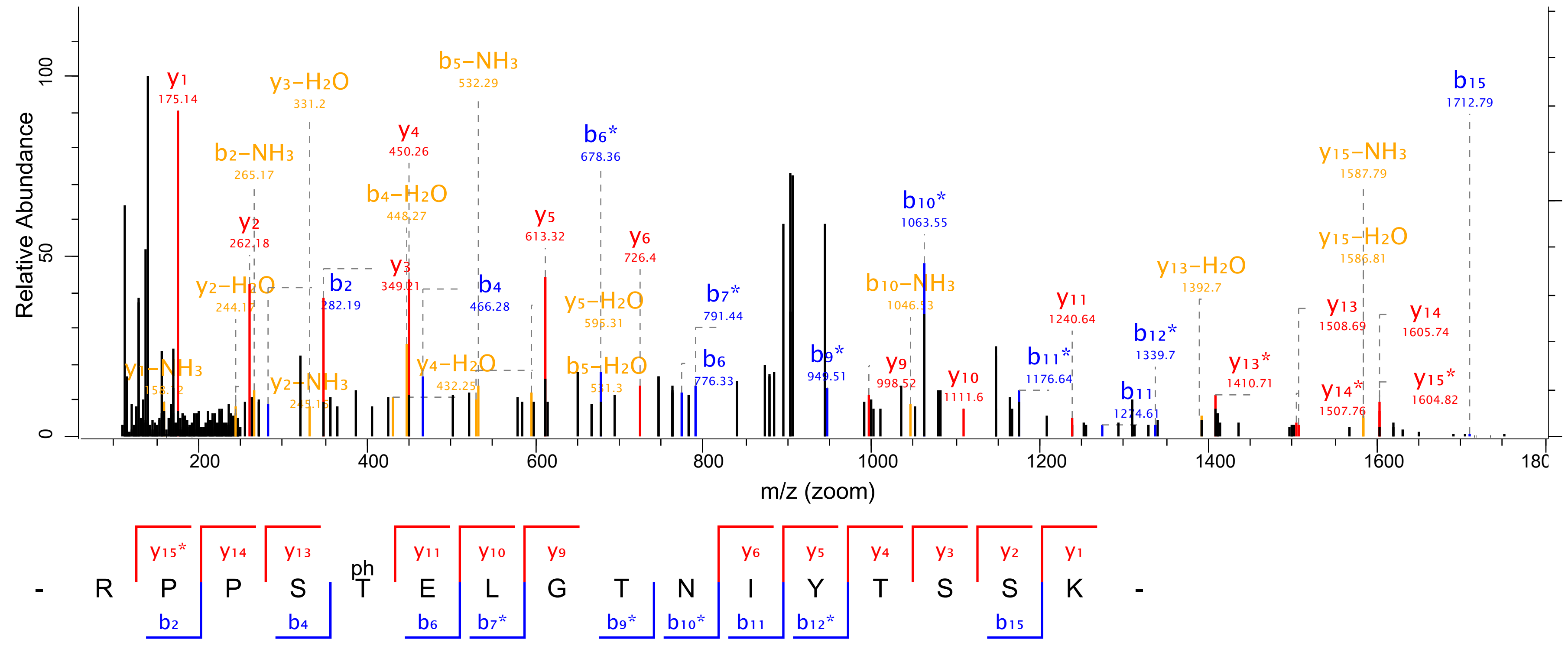

Protein	Accession	Phosphosite
FLAG-TcMET	A6MUT7	T460

Raw File	Scan	Method	Score	m/z
171017_TomN_B1_6uL	24656	ITMS; CID	106.87	943.97

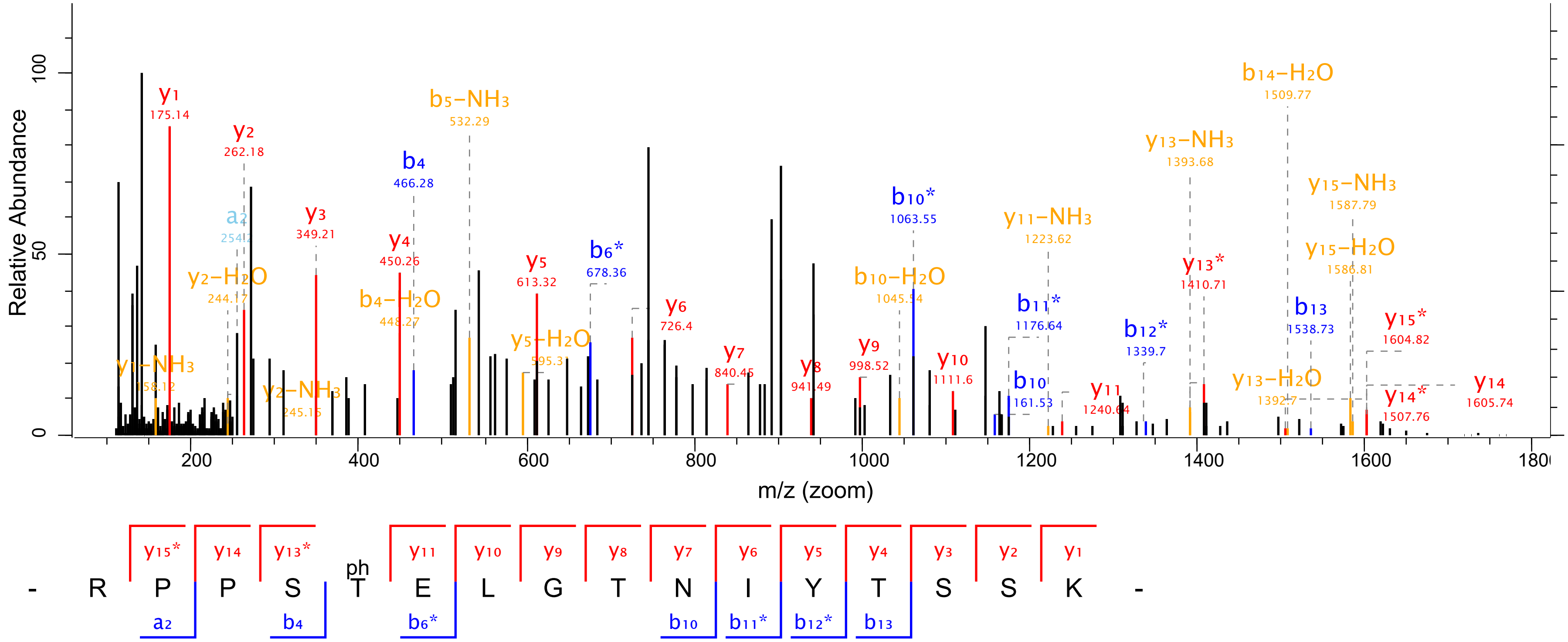

Protein	Accession	Phosphosite
FLAG-TcMET	A6MUT7	T460

Raw File	Scan	Method	Score	m/z
171017_TomN_B1_6uL	24642	ITMS; CID	69.02	633.67

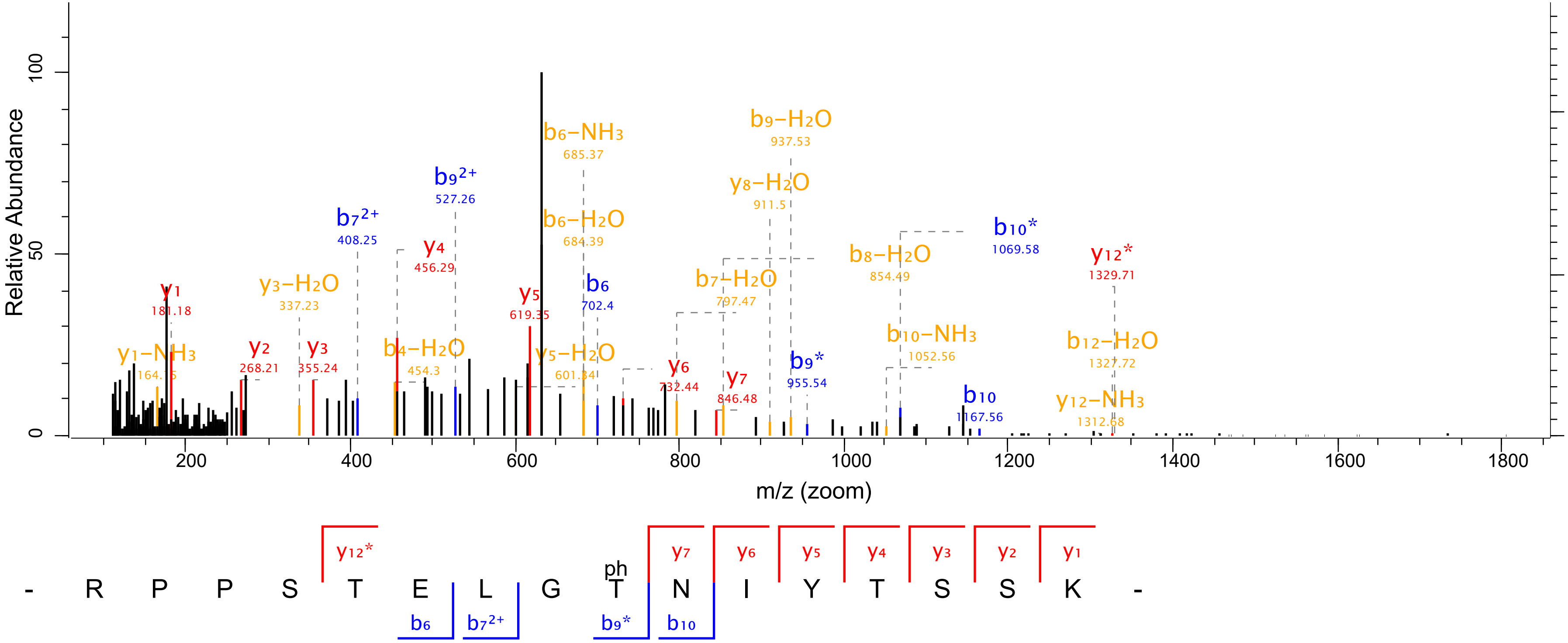

Protein	Accession	Phosphosite
FLAG-TcMET	A6MUT7	T464

Raw File	Scan	Method	Score	m/z
171207_TomN_A3	9158	FTMS; CID	124.2	686.04

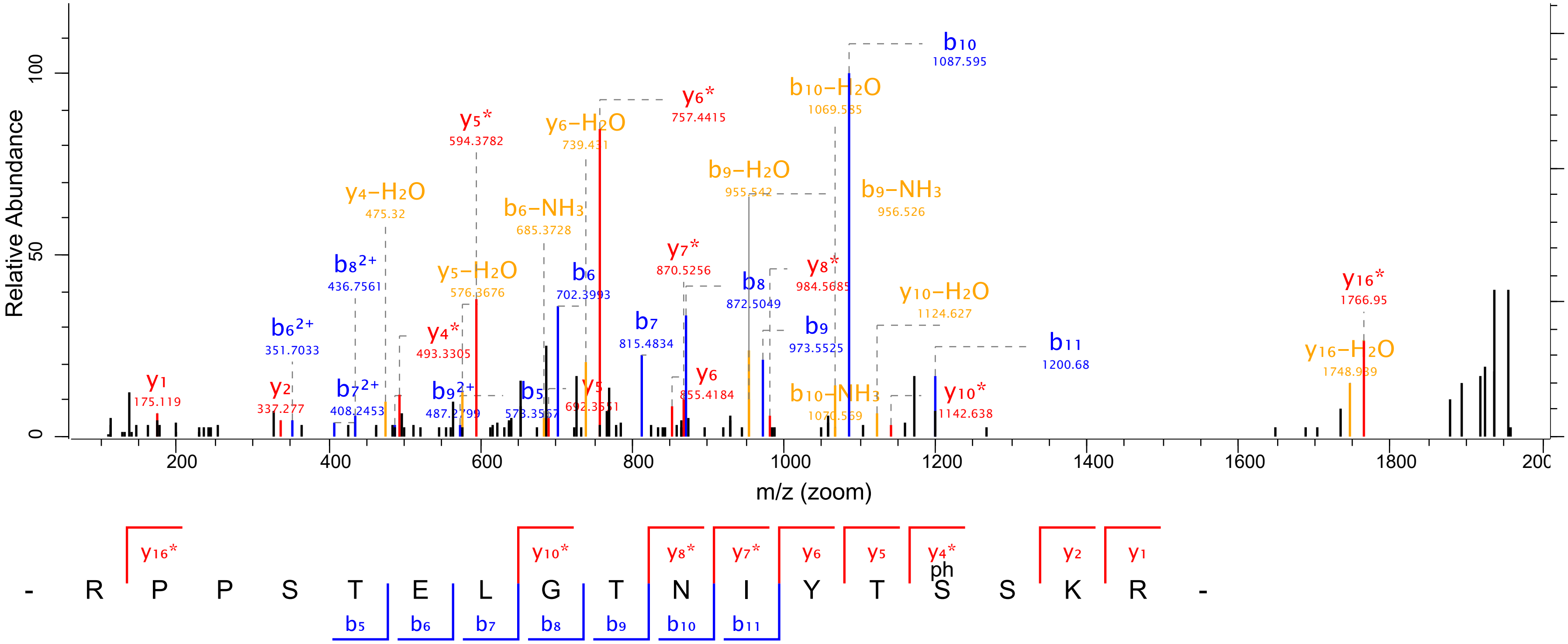

Protein	Accession	Phosphosite
FLAG-TcMET	A6MUT7	S469

Raw File	Scan	Method	Score	m/z
171207_TomN_A3	13211	FTMS; CID	112.56	629.65

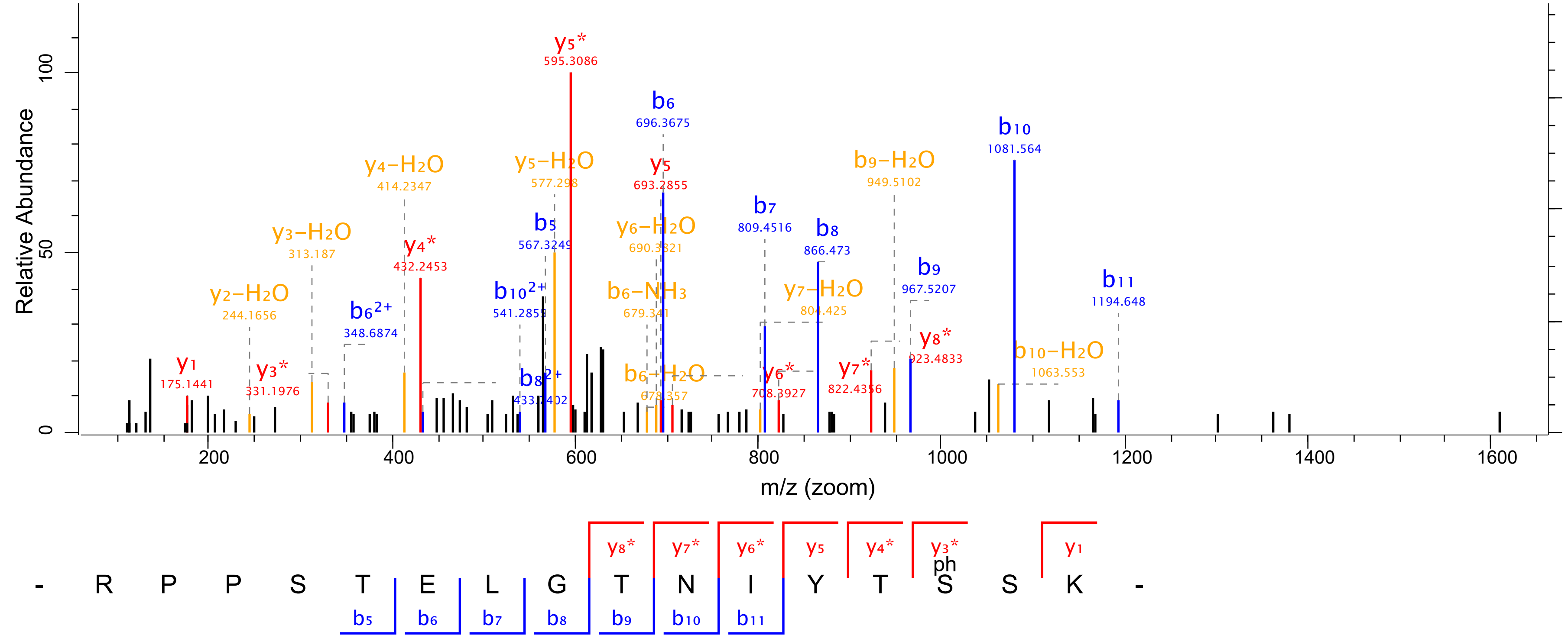

Protein	Accession	Phosphosite
FLAG-TcMET	A6MUT7	S469

Raw File	Scan	Method	Score	m/z
171207_TomN_A1	13073	FTMS; CID	99.09	629.98

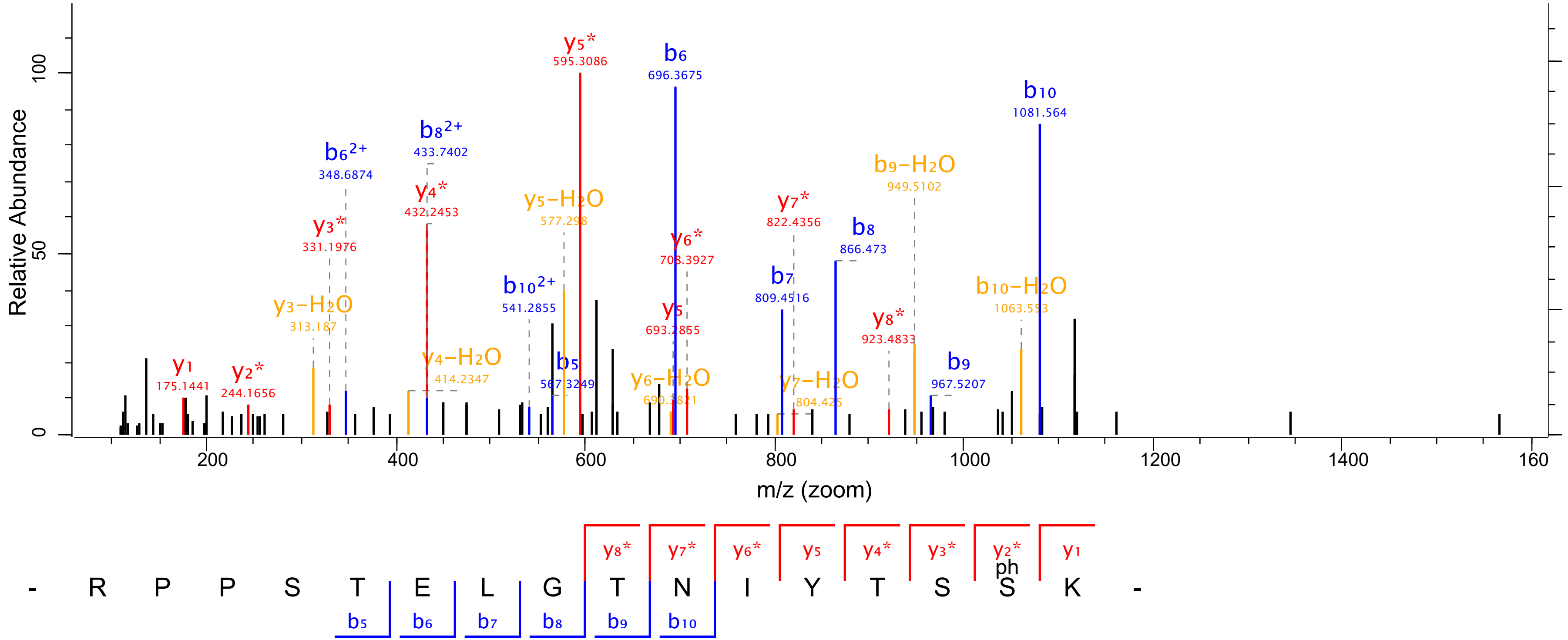

Protein	Accession	Phosphosite
FLAG-TcMET	A6MUT7	S470

Raw File	Scan	Method	Score	m/z
171017_TomN_A2_6uL	22826	ITMS; CID	298.31	990.47

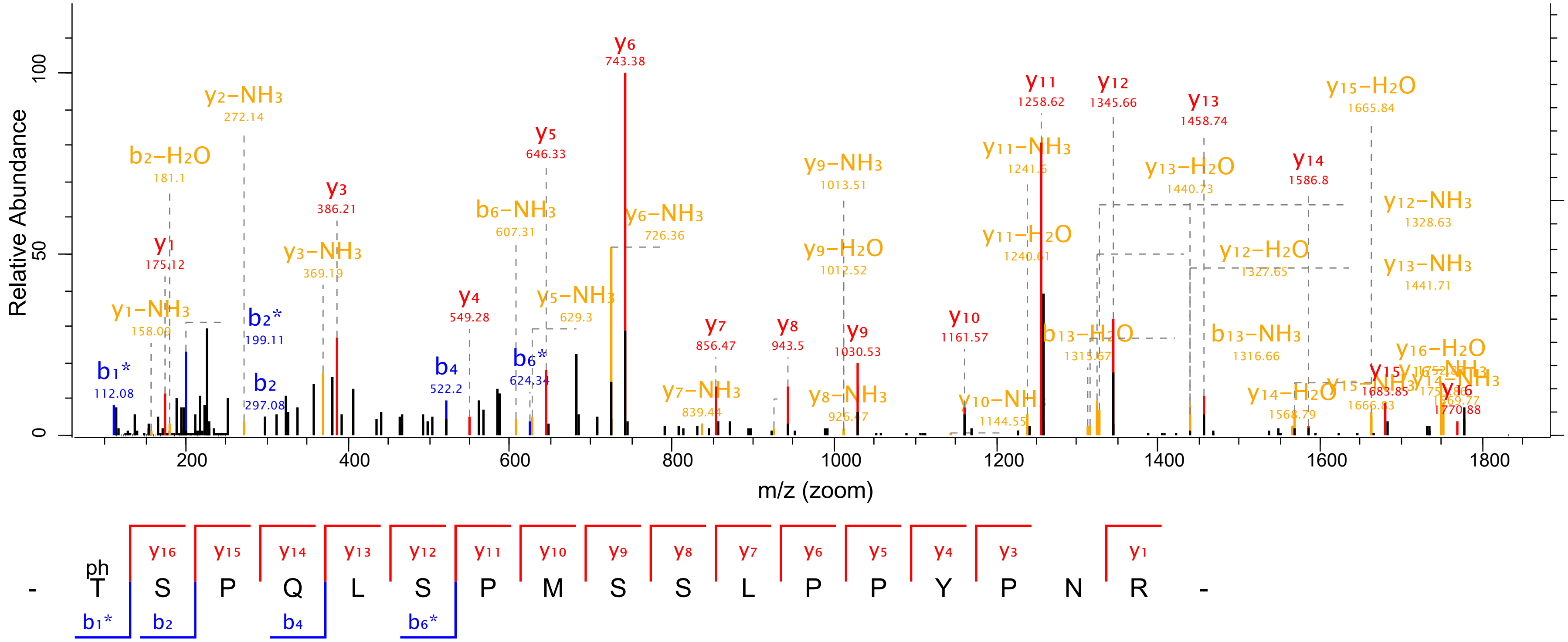

Protein	Accession	Phosphosite
FLAG-TcMET	A6MUT7	T475

Raw File	Scan	Method	Score	m/z
171207_TomN_B2	26391	FTMS; CID	165.7	990.47

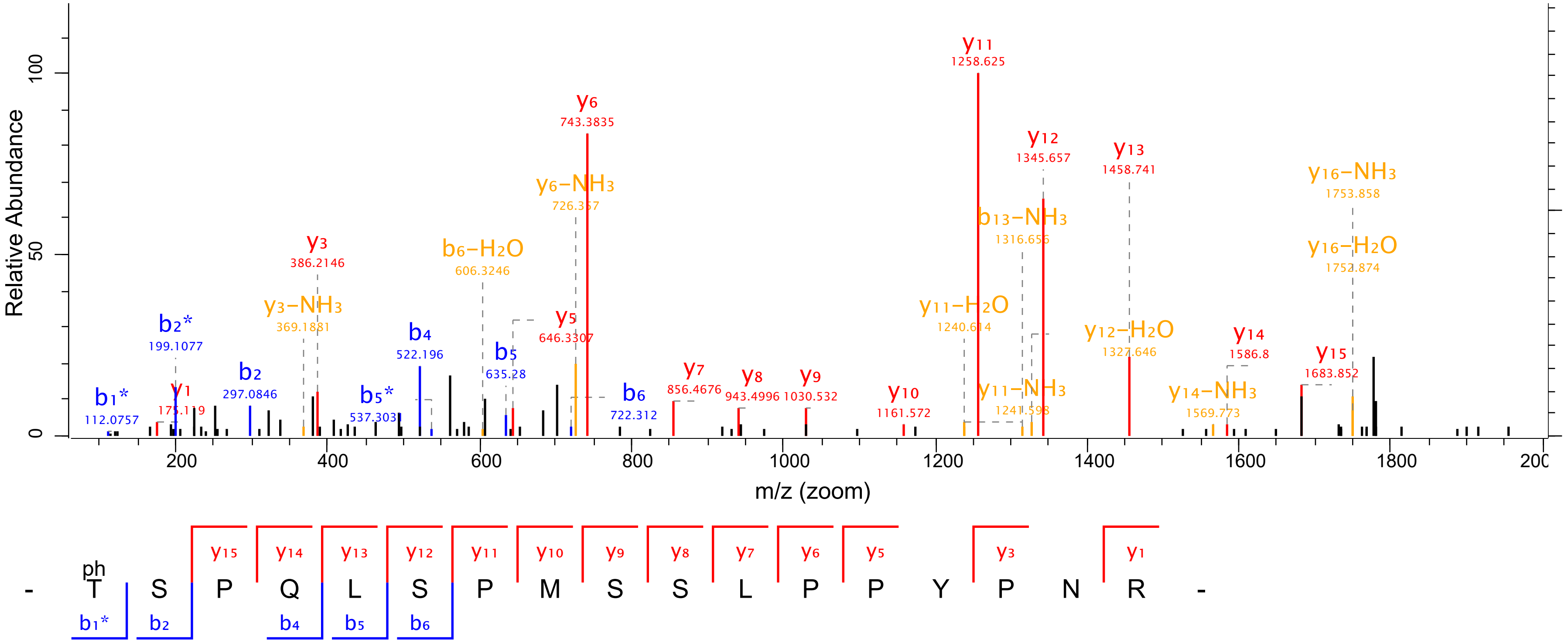

Protein	Accession	Phosphosite
FLAG-TcMET	A6MUT7	T475

Raw File 171017_TomN_B2_6uL | Scan 21614 | Method ITMS; CID | Score 224.36 | m/z 1033.47

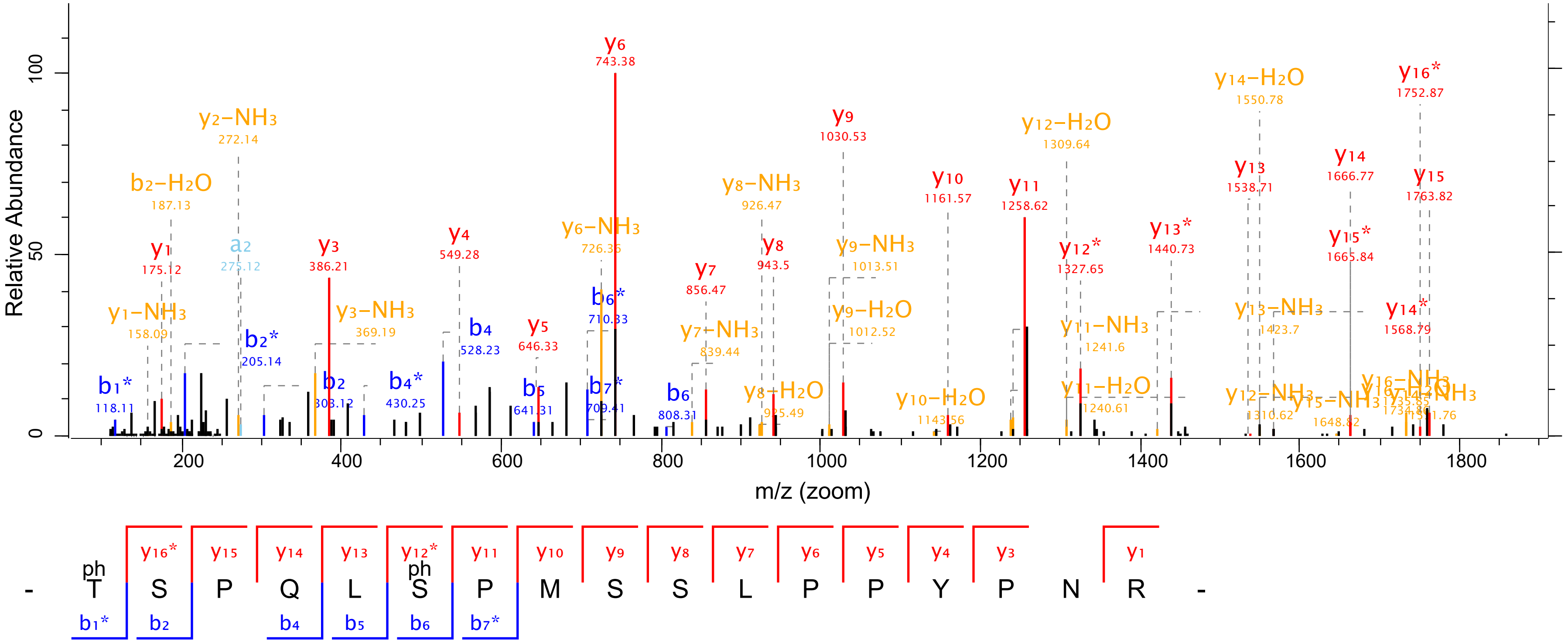

Protein FLAG-TcMET | Accession A6MUT7 | Phosphosite T475, S480

Raw File	Scan	Method	Score	m/z
171207_TomN_A2	30465	FTMS; CID	194.58	1033.47

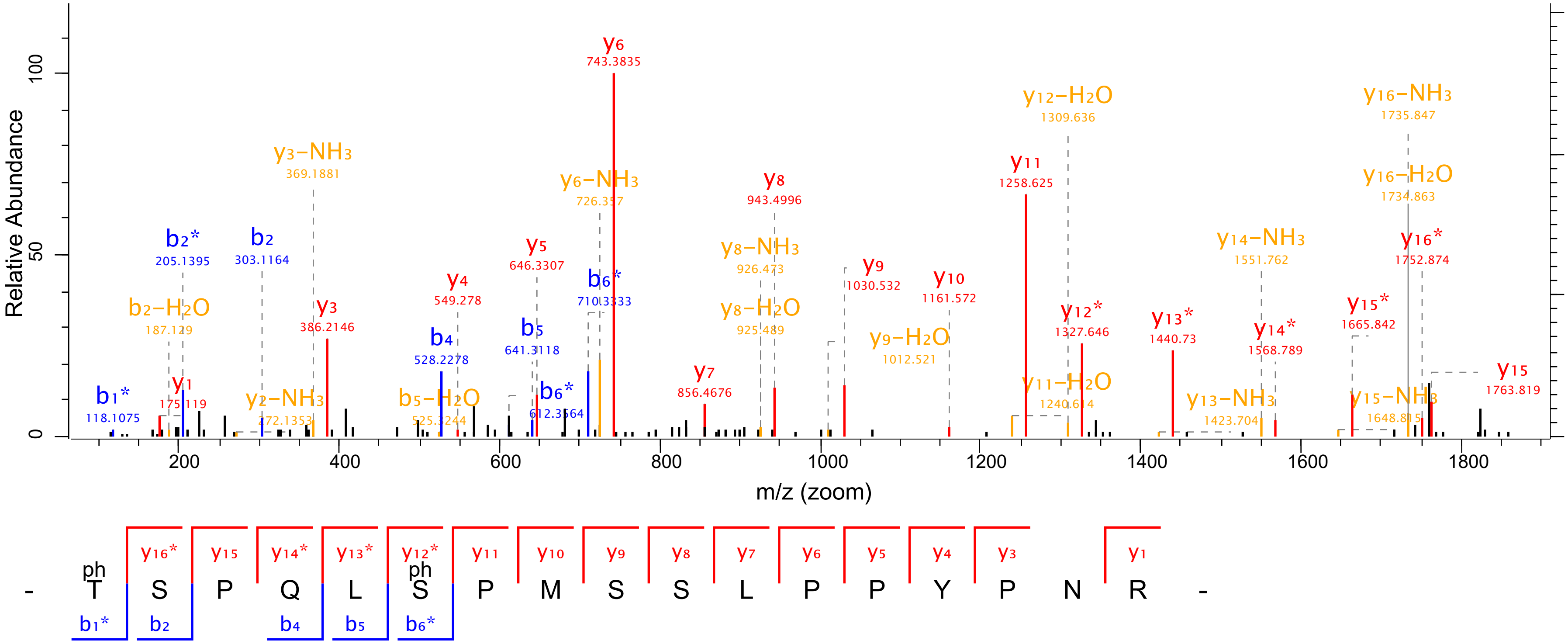

Protein	Accession	Phosphosite
FLAG-TcMET	A6MUT7	T475, S480

Raw File	Scan	Method	Score	m/z
171207_TomN_A2	27348	FTMS; CID	251.98	1041.47

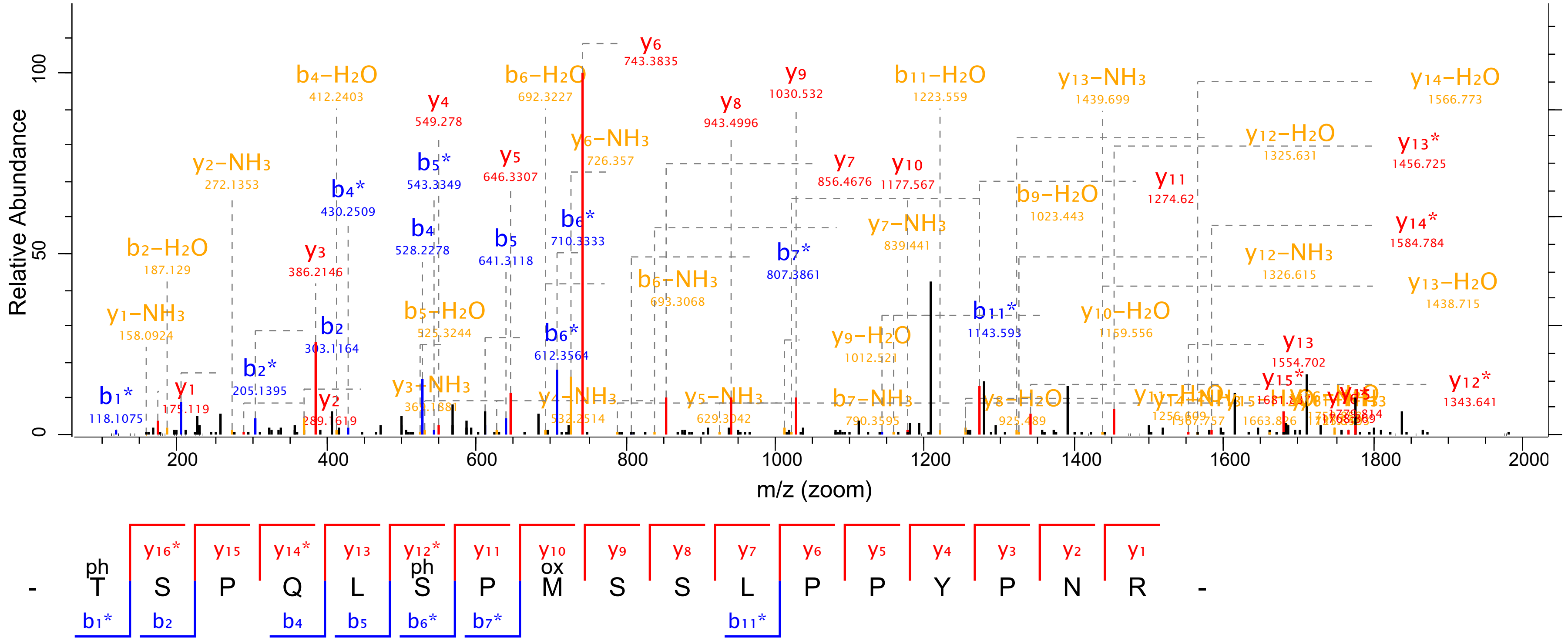

Protein	Accession	Phosphosite
FLAG-TcMET	A6MUT7	T475, S480

Raw File	Scan	Method	Score	m/z
171207_TomN_A2	20947	FTMS; CID	133.47	998.97

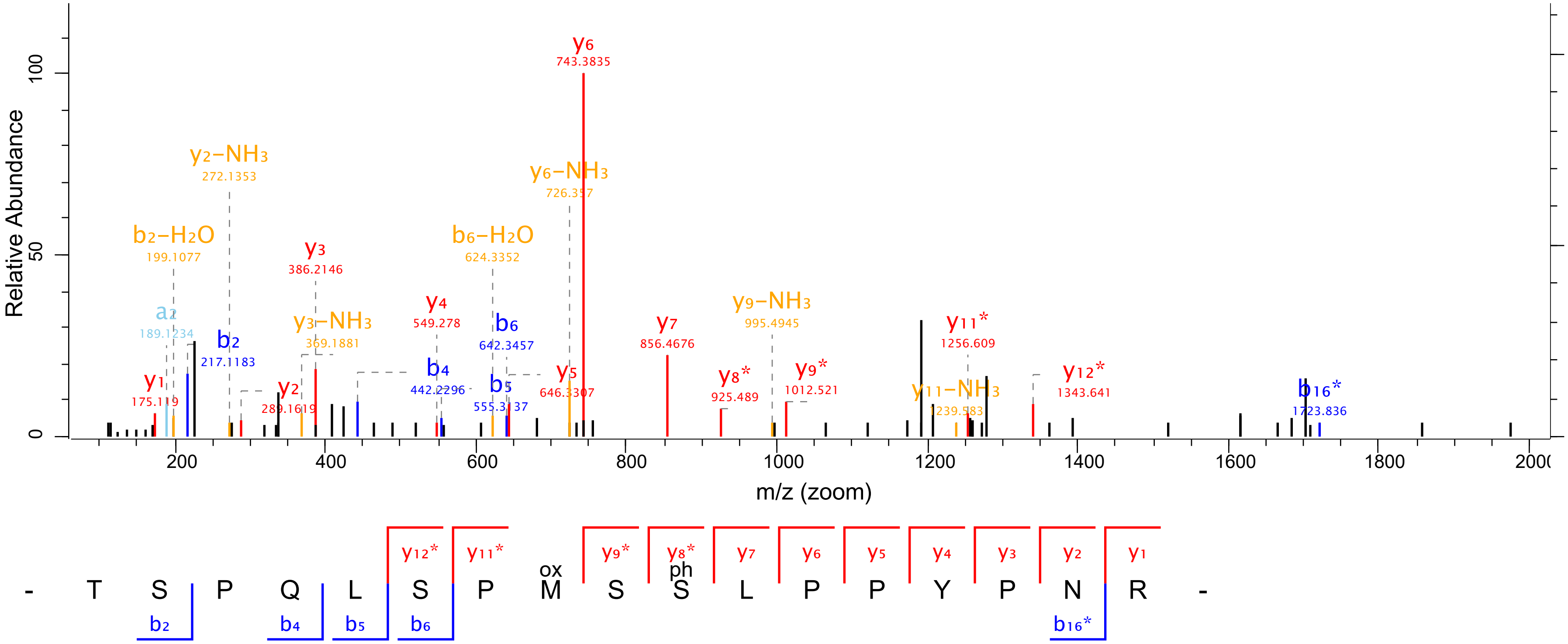

Protein	Accession	Phosphosite
FLAG-TcMET	A6MUT7	S484

Raw File	Scan	Method	Score	m/z
171017_TomN_B3_6uL	23751	ITMS; CID	199.91	1136.07

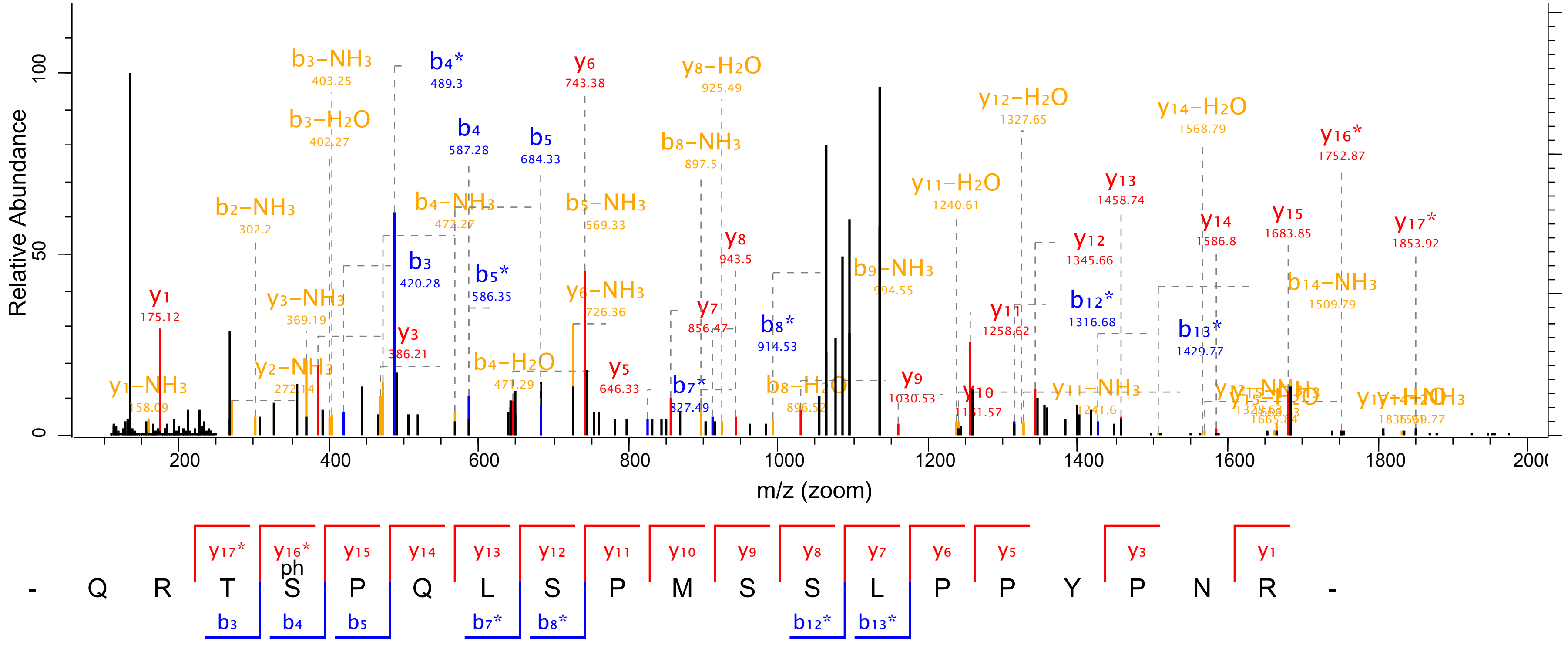

Protein	Accession	Phosphosite
FLAG-TcMET	A6MUT7	S476

Raw File	Scan	Method	Score	m/z
171017_TomN_B2_6uL	18702	ITMS; CID	157.42	1176.05

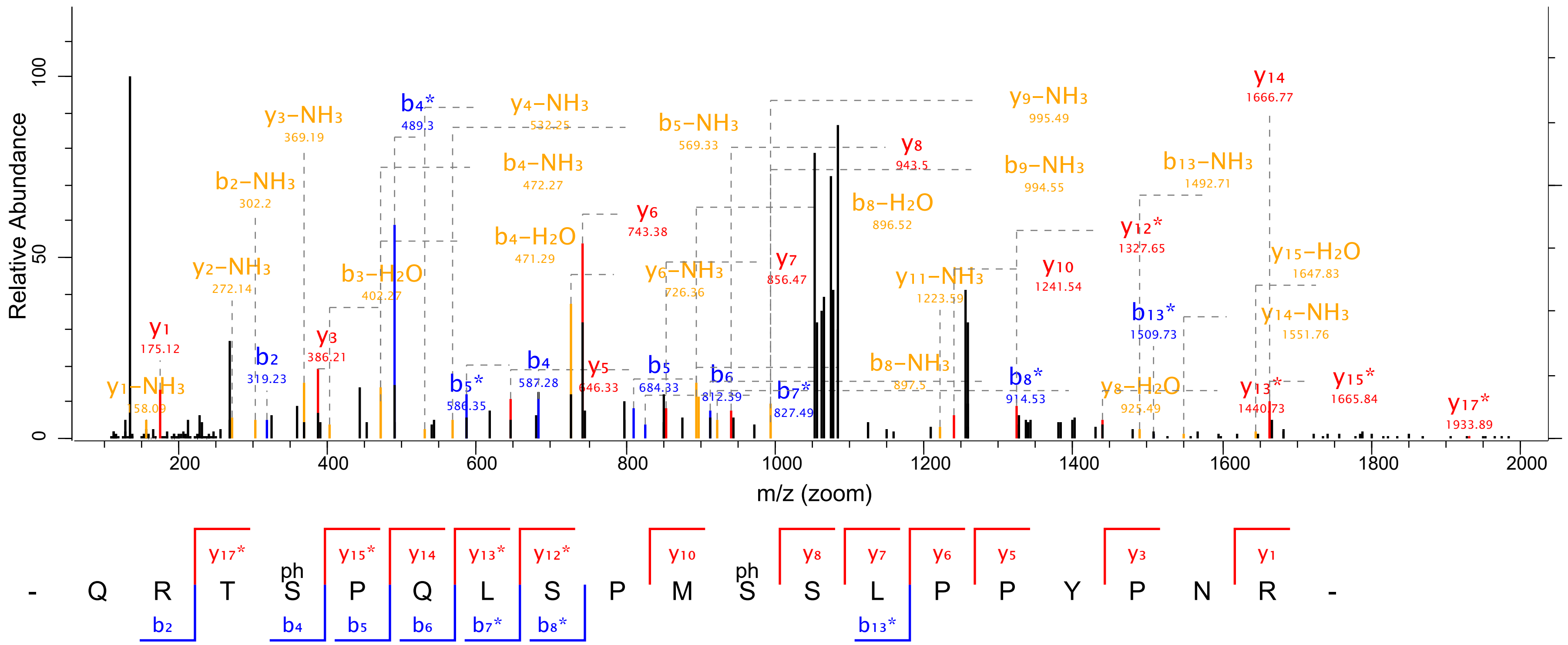

Protein	Accession	Phosphosite
FLAG-TcMET	A6MUT7	S476, S483

Raw File	Scan	Method	Score	m/z
171017_TomN_B3	25081	ITMS; CID	116.85	1172.53

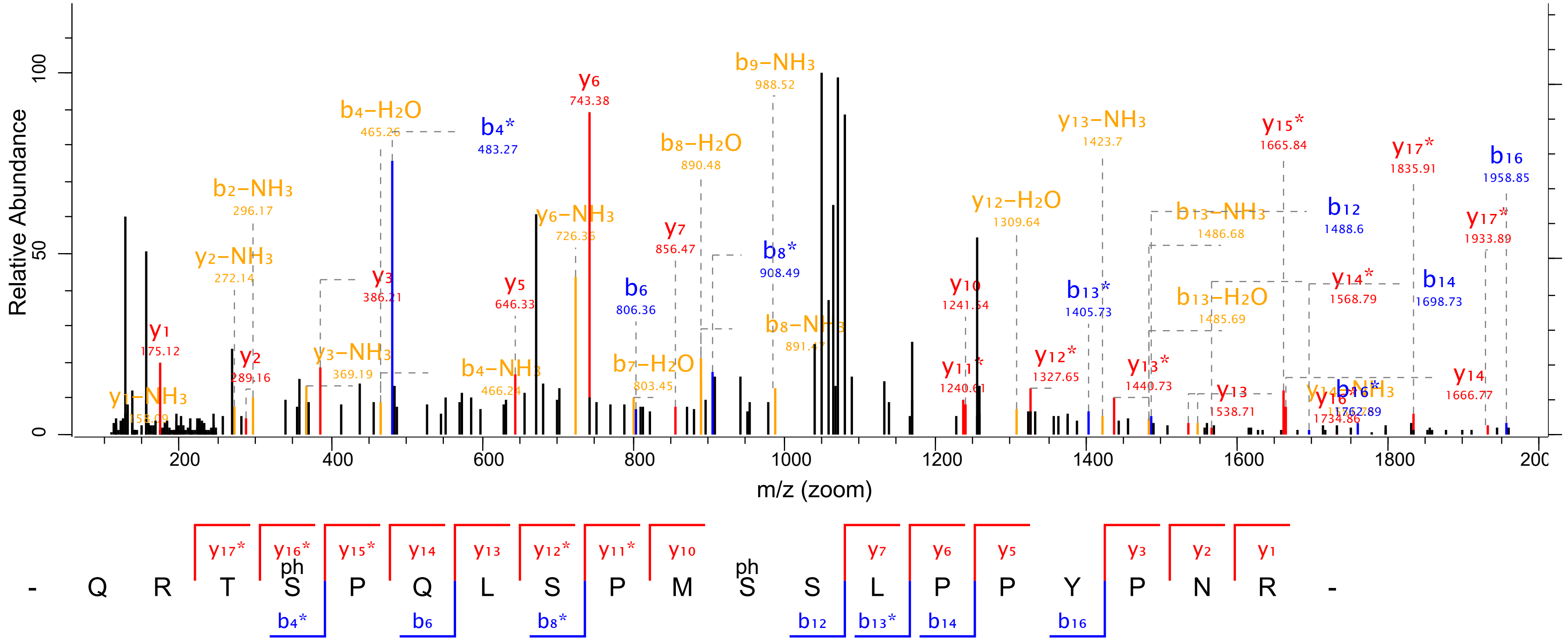

Protein	Accession	Phosphosite
FLAG-TcMET	A6MUT7	S476, S483

Raw File	Scan	Method	Score	m/z
171207_TomN_B2	19962	FTMS; CID	127.91	1181.03

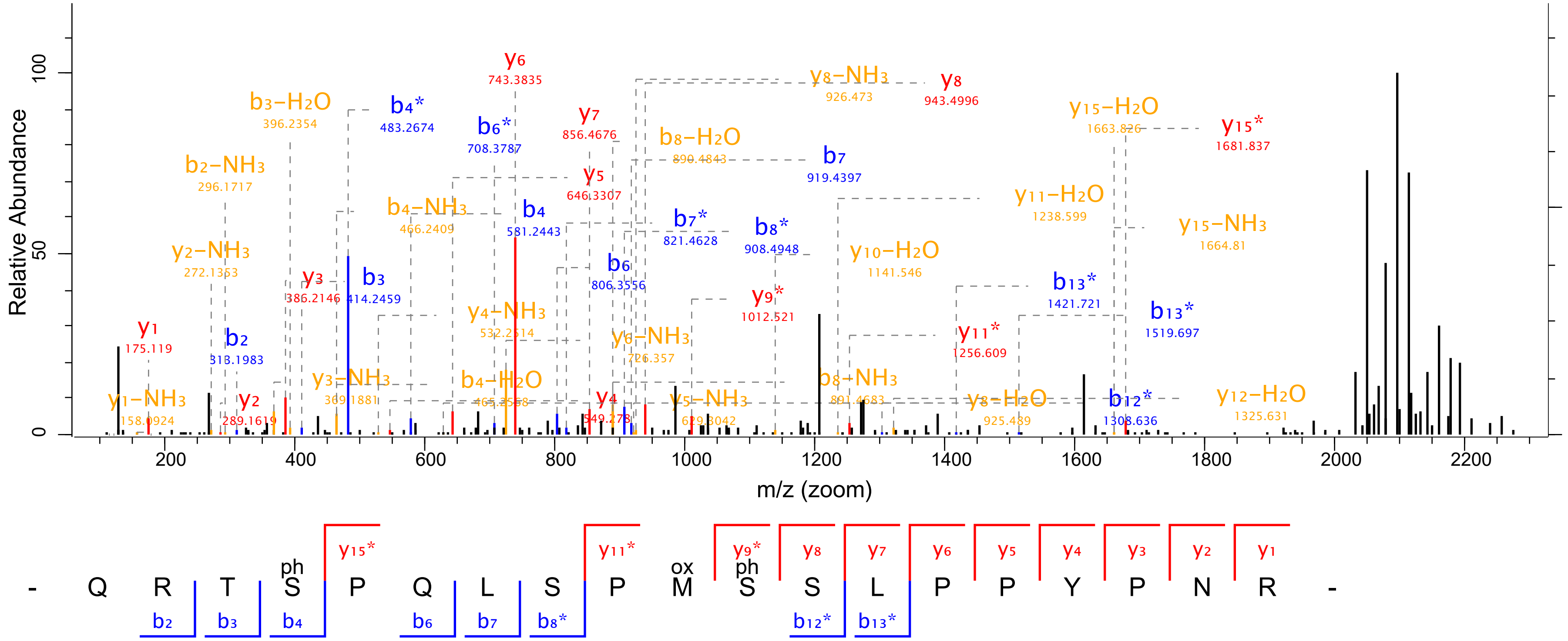

Protein	Accession	Phosphosite
FLAG-TcMET	A6MUT7	S476, S483

Raw File	Scan	Method	Score	m/z
171207_TomN_B2	22627	FTMS; CID	73.29	913.12

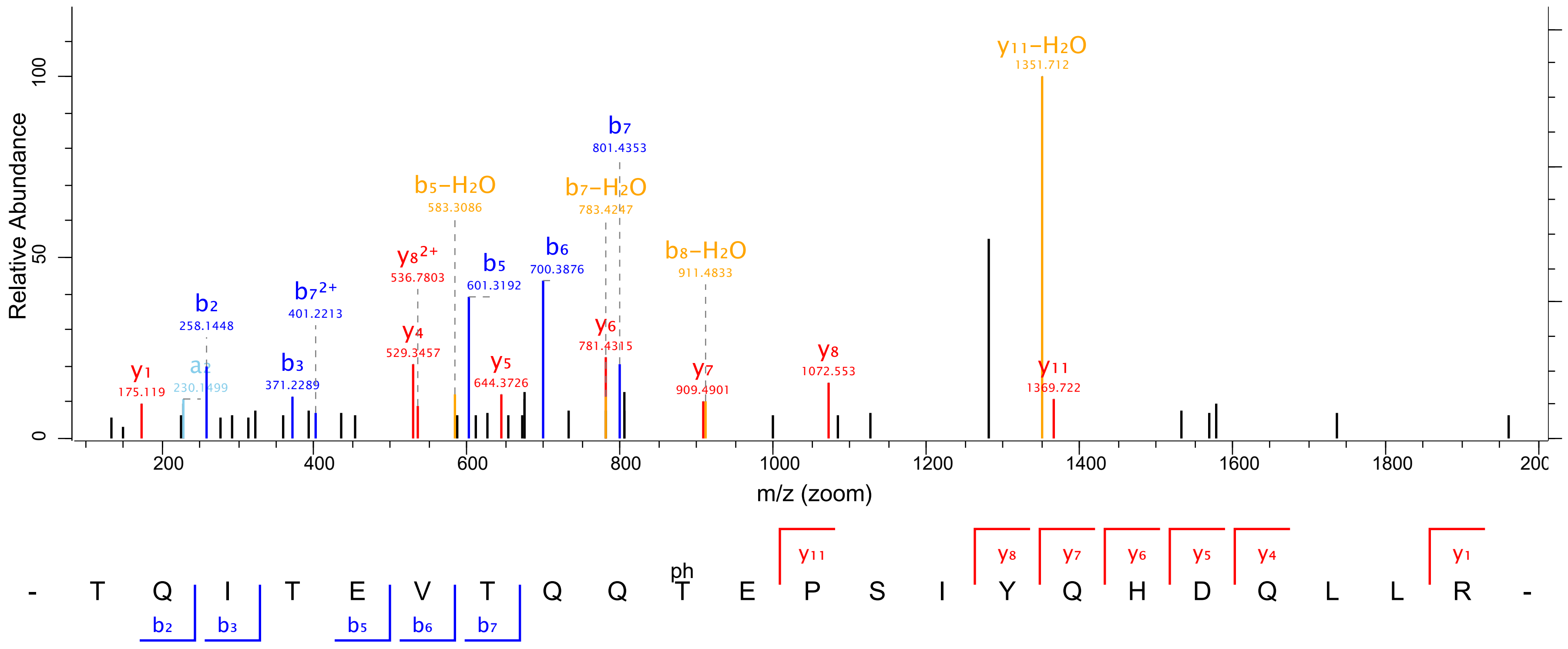

Protein	Accession	Phosphosite
FLAG-TcMET	A6MUT7	T501

Raw File	Scan	Method	Score	m/z
171207_TomN_A3	23103	FTMS; CID	127.38	913.12

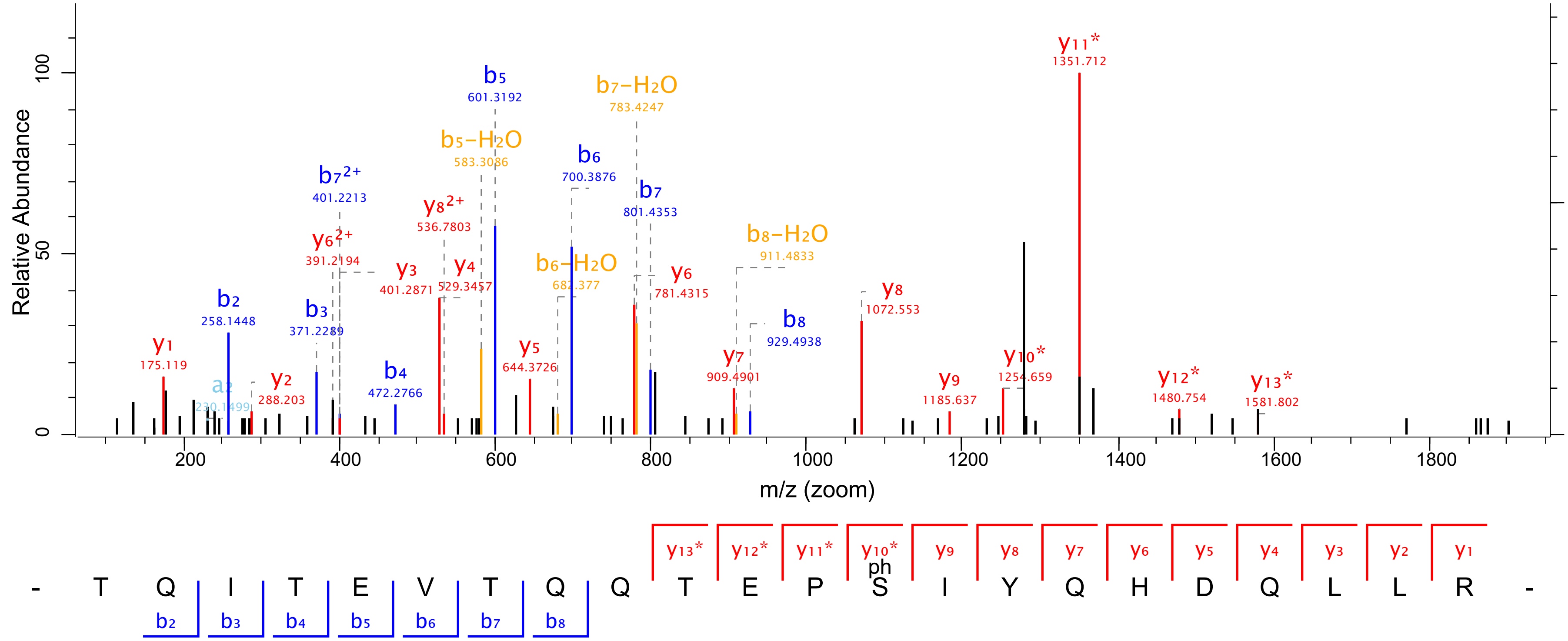

Protein	Accession	Phosphosite
FLAG-TcMET	A6MUT7	S504

Raw File	Scan	Method	Score	m/z
171207_TomN_A2	23091	FTMS; CID	96.02	913.11

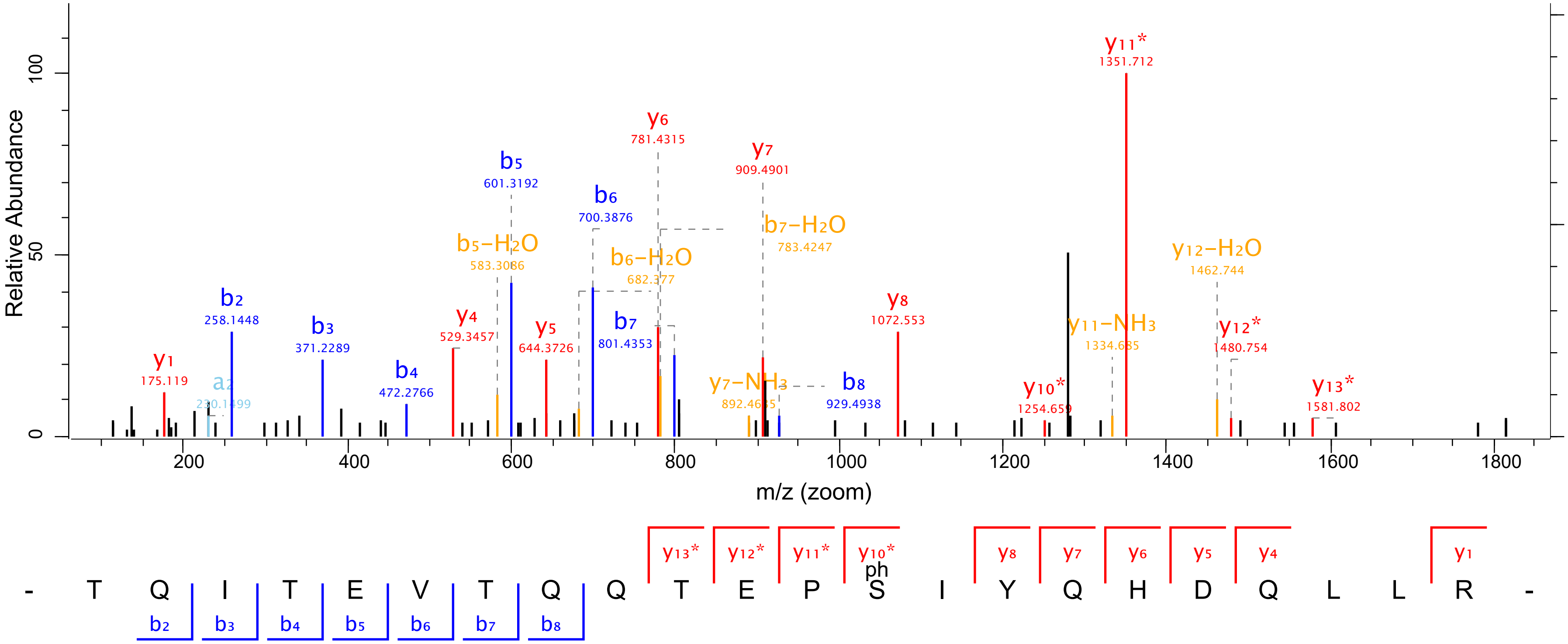

Protein	Accession	Phosphosite
FLAG-TcMET	A6MUT7	S504

Raw File	Scan	Method	Score	m/z
171207_TomN_A3	4771	FTMS; CID	66.27	473.73

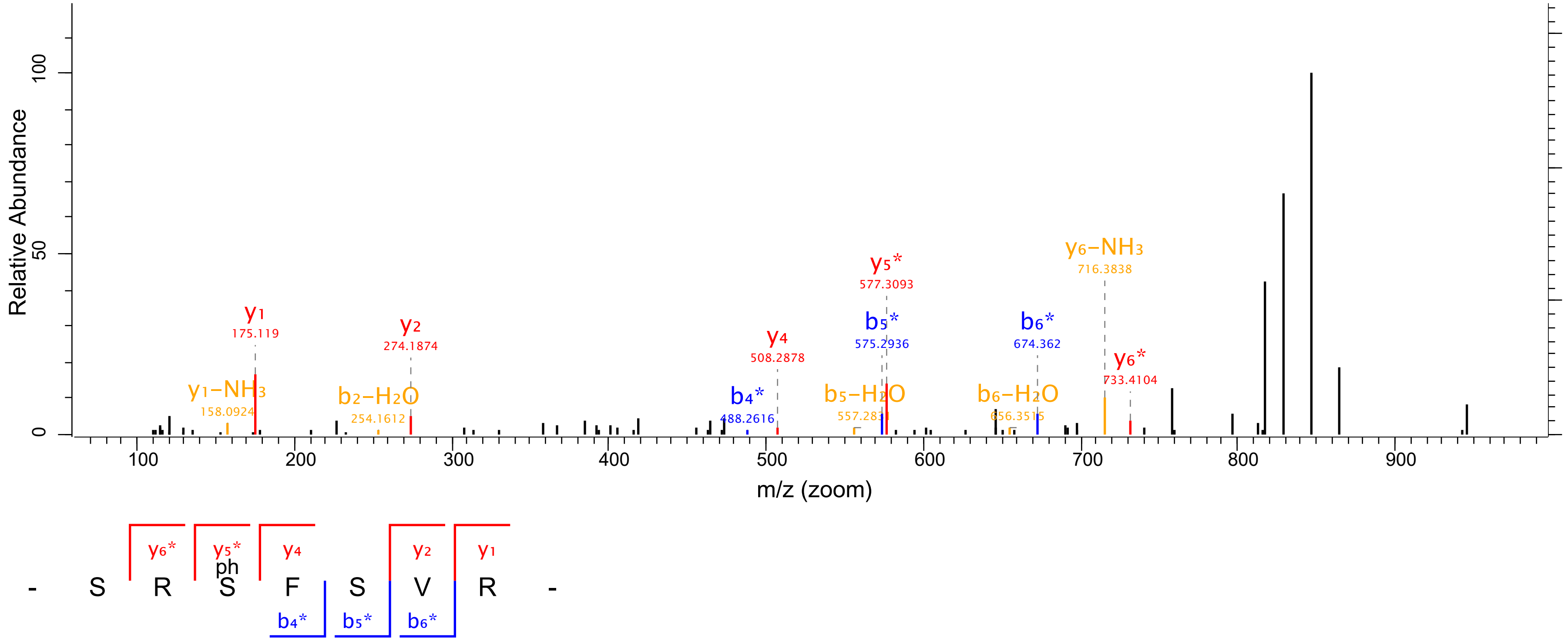

Protein	Accession	Phosphosite
His$_6$-TcTAI	S6B9A5	S227

Raw File	Scan	Method	Score	m/z
171207_TomN_A2	4775	FTMS; CID	60.83	473.73

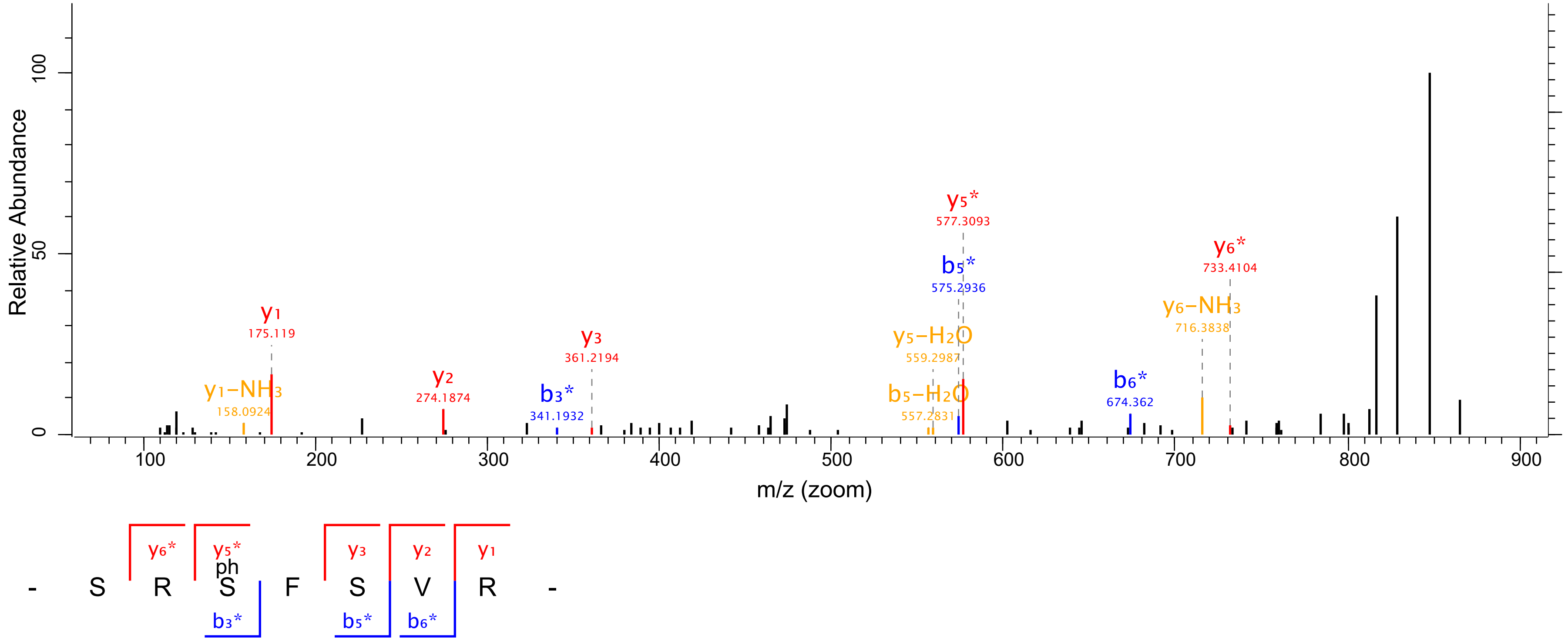

Protein	Accession	Phosphosite
His$_6$-TcTAI	S6B9A5	S227

Raw File	Scan	Method	Score	m/z
171207_TomN_B1	4551	FTMS; CID	50.04	473.73

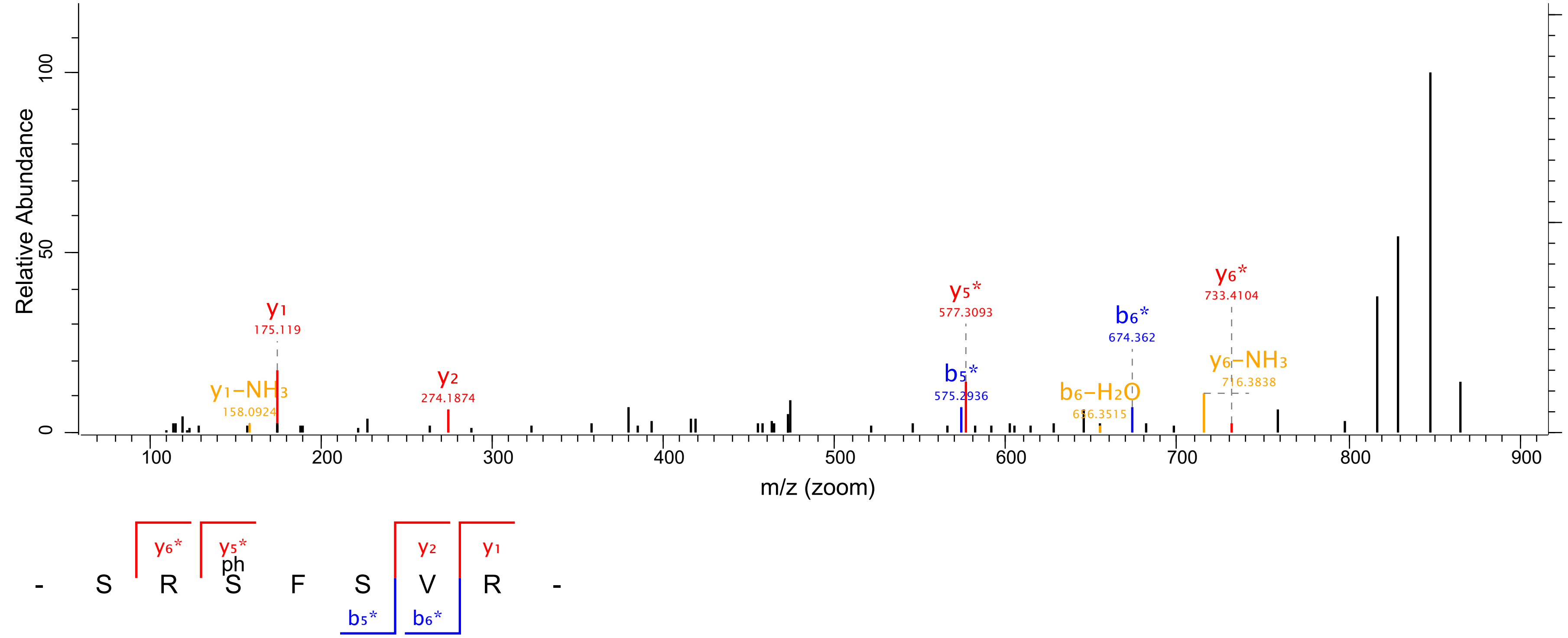

Protein	Accession	Phosphosite
His$_6$-TcTAI	S6B9A5	S227

Raw File	Scan	Method	Score	m/z
171207_TomN_A3	30363	FTMS; CID	126.88	1271.06

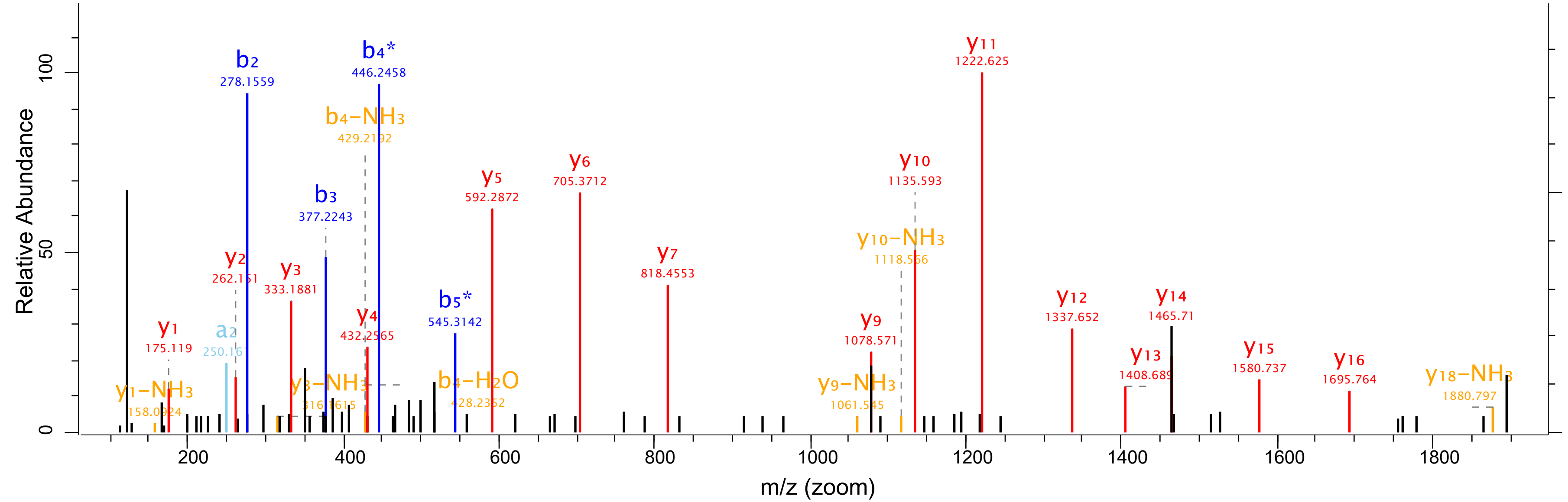

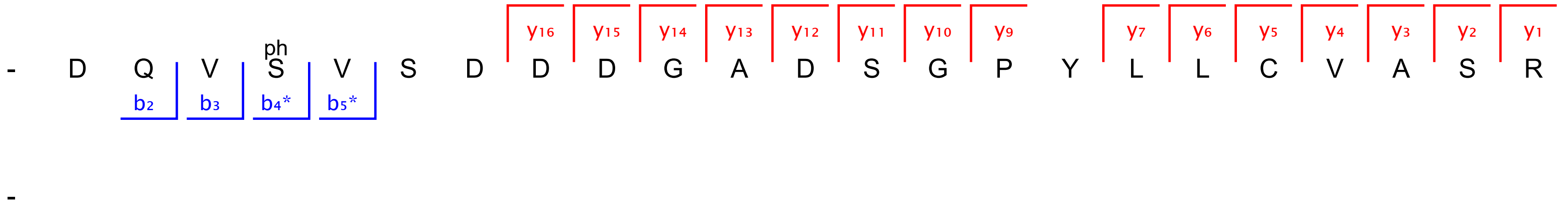

Protein	Accession	Phosphosite
His$_6$-TcTAI	S6B9A5	S269

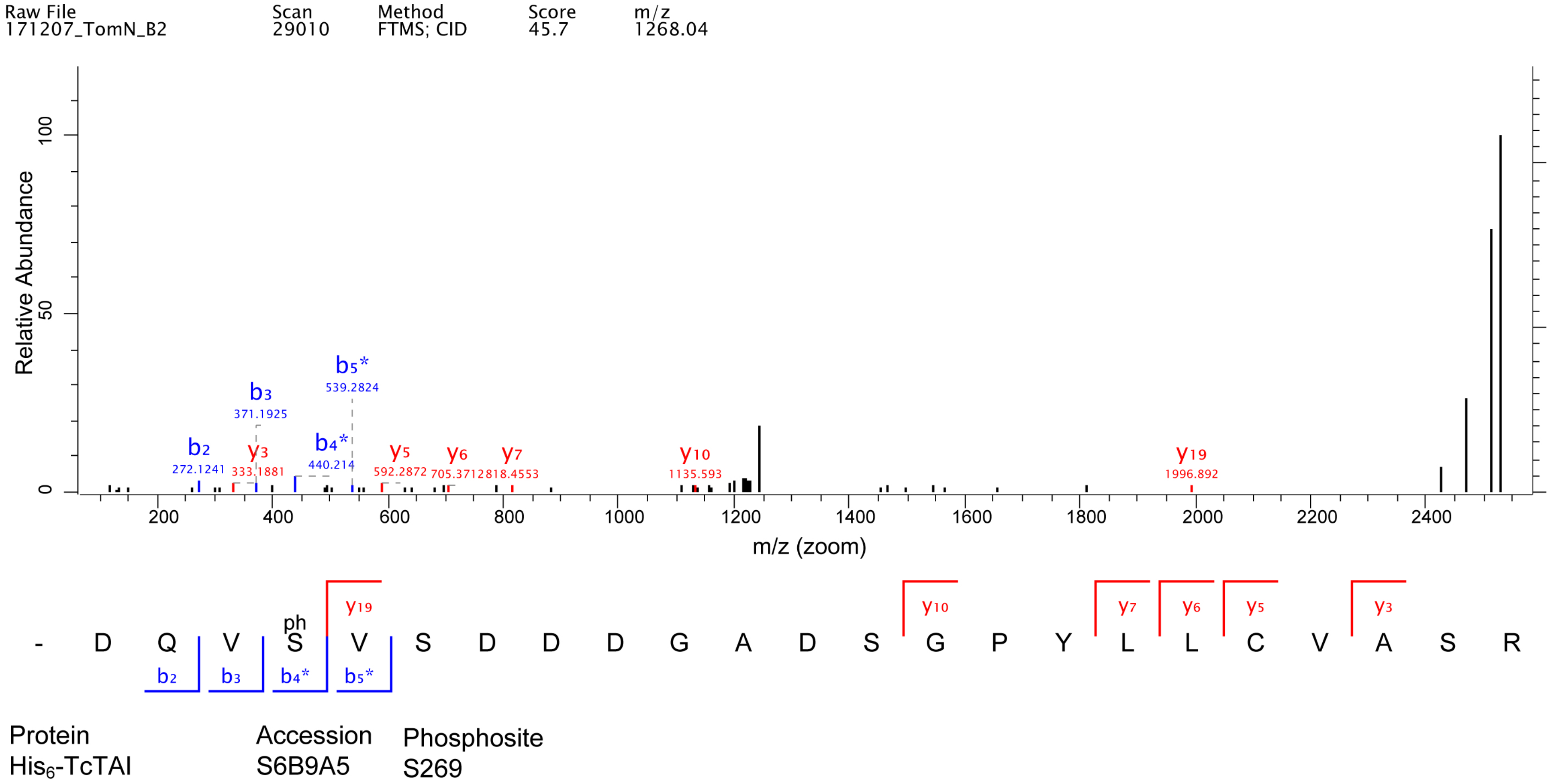

Figure S5. Annotated MS/MS evidence spectra of affinity-purified TcJHR complexes based on quantitative phosphoproteomics analyses. The spectra of isotopically labeled phosphopeptide identifications are based on MaxQuant MS data searches, as summarized in Table 2. Major singly or doubly charged (2+) b-ions and y-ions are highlighted in blue and red and minor a-ions in light blue, respectively. Matching neutral loss of phosphate (*), neutral loss of water (-H_2O), and neutral loss of ammonium (-NH_3) are highlighted in yellow. Column headers (top): Raw File, corresponding MS raw data file name; Scan, corresponding MS/MS scan number; Method, Fourier-transform mass spectrometry (FTMS) collision induced fragmentation (CID); Score, MaxQuant score; m/z, mass over charge of precursor ion.

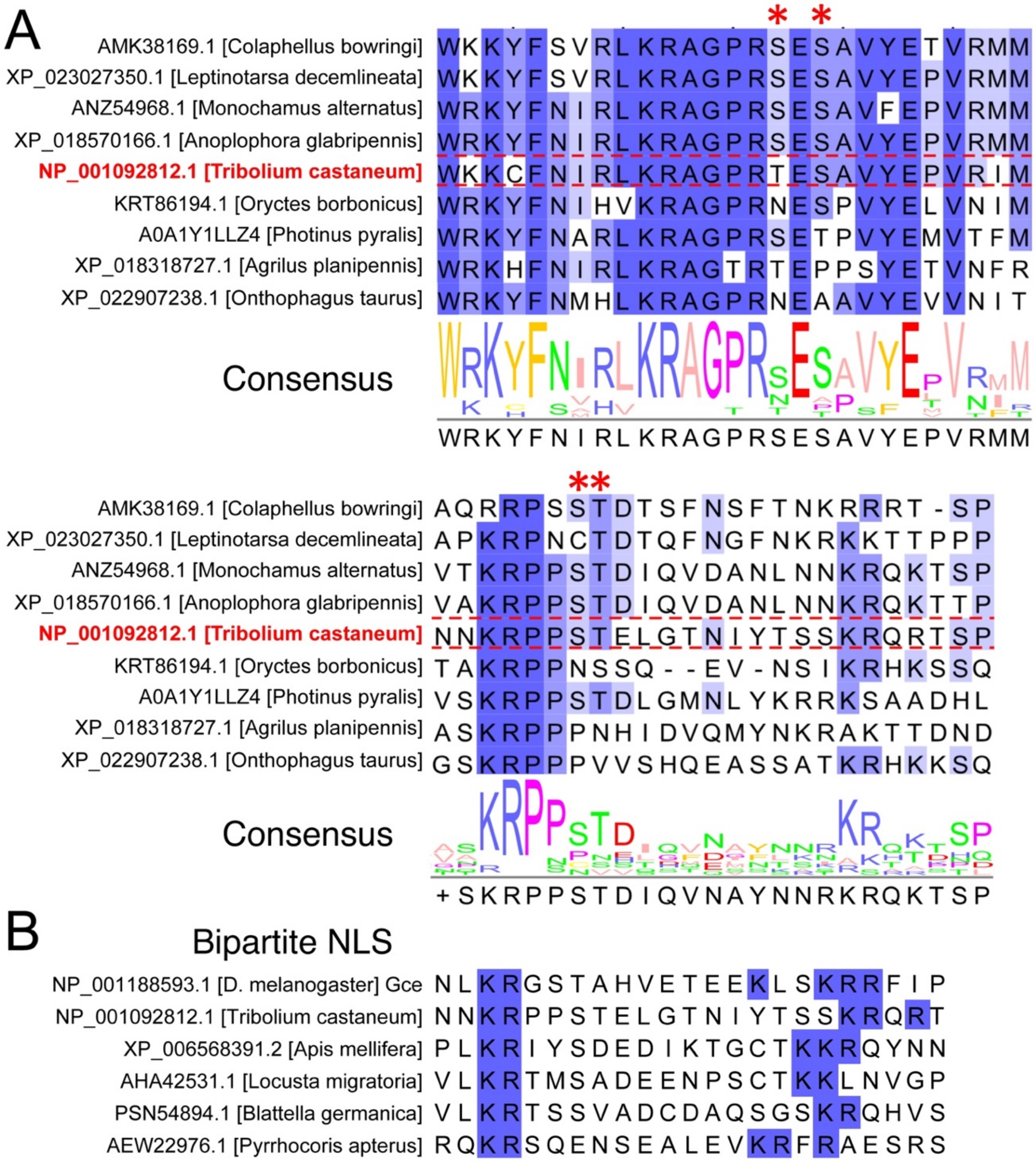

Figure S6. Alignment of MET protein sequences surrounding methoprene-induced phosphorylation sites and a bipartite NLS. *A*, sequences from nine indicated species of beetles (Coleoptera) are aligned around the serine and threonine residues (red asterisks) phosphorylated in response to methoprene treatment in TcMET (outlined in red). The consensus sequences indicate the degree of conservation. Top: sequences within the PAS-A domain; bottom: sequences straddling the bipartite NLS. *B*, bipartite NLSs, functionally confirmed in *D. melanogaster* Gce and TcMET proteins, may be conserved across distant insect orders. Codes preceding all sequences indicate NCBI accession numbers.

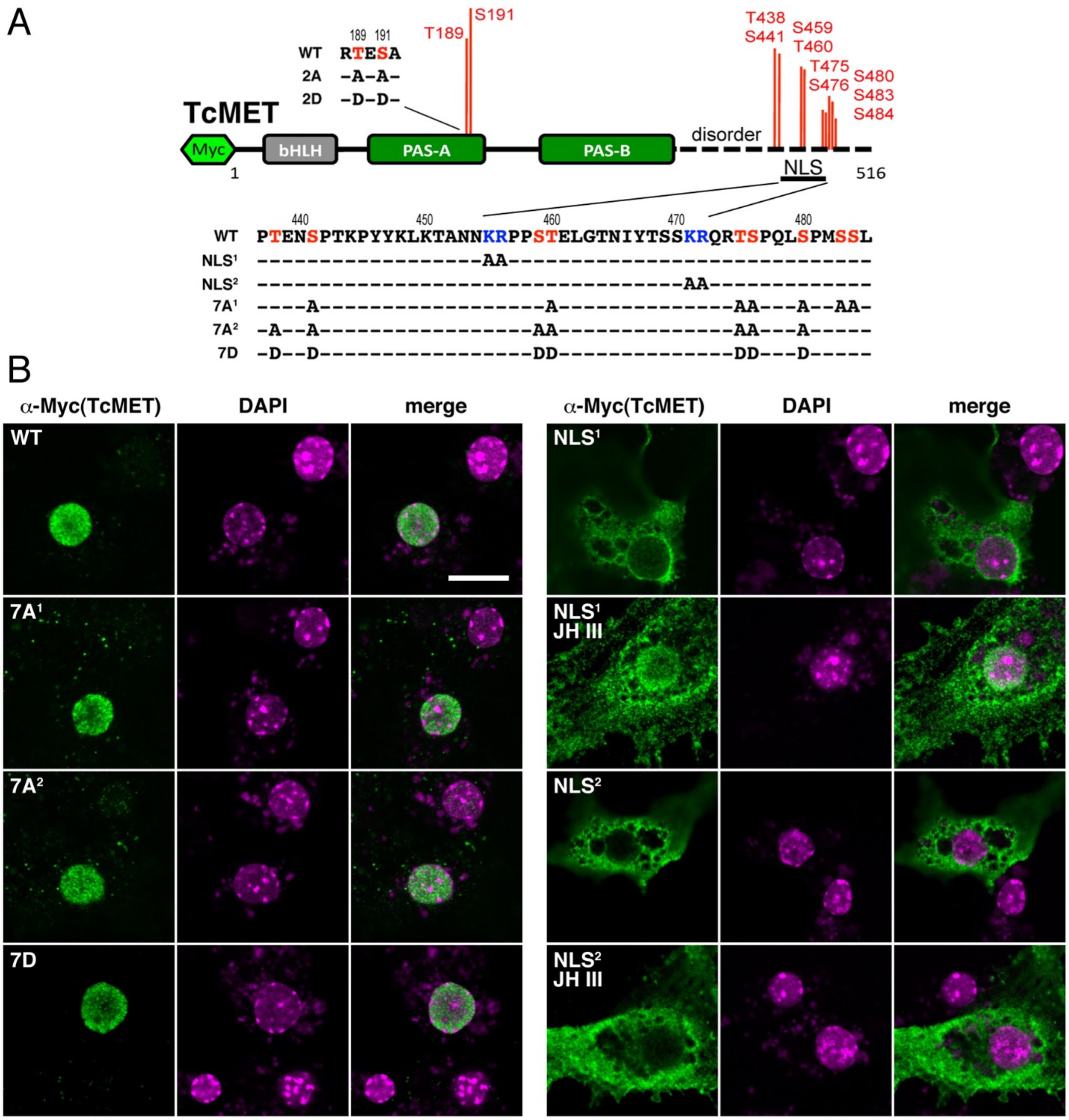

Figure S7. Effects of mutations and JH III on the localization of TcMET in *T. castaneum* TcA cells. *A*, mutated variants of TcMET. Basic residues (KR, in blue) constituting the basic clusters of a bipartite NLS were mutated to A (variants NLS^1 and NLS^2). Subsets of residues that are subject to methoprene-dependent or independent phosphorylation (in red) were substituted either with alanine (variants 2A, $7A^1$, and $7A^2$) or with aspartic acid (2D and 7D). *B*, the WT TcMET or its mutated variants, all carrying an N-terminal Myc epitope, were expressed in transfected TcA cells and detected using an anti-Myc antibody (green); DNA was stained with DAPI (magenta). Representative examples of Myc-TcMET localization are single confocal slices. Scale bar (10 µm) applies to all images.

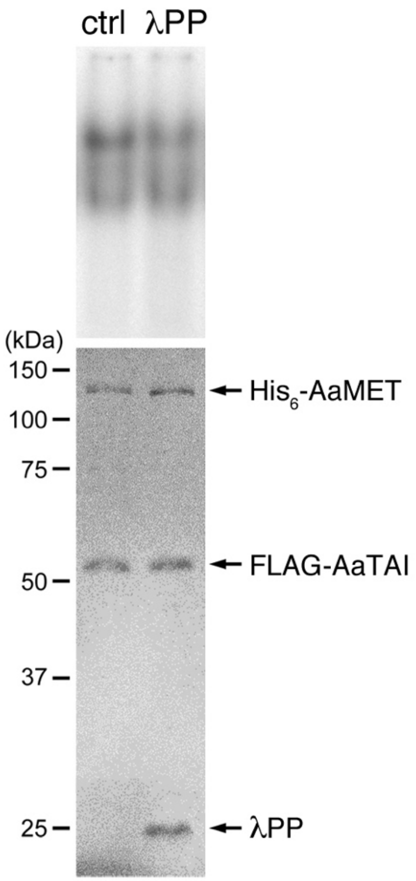

Figure S8. Phosphatase treatment of AaJHR does not prevent its interaction with the JHRE DNA. The EMSA (top) was performed with the recombinant AaJHR protein complex purified from the *Sf9* cells. Prior to incubation with the MFBS1 JHRE DNA probe, the protein was treated with 40 U/μl of λPP for 1 h at 4°C. For control, AaJHR was incubated for 1 h at 4°C in the buffer alone. Integrity of the proteins following the λPP treatment was verified on a Coomassie-stained SDS-PAGE (bottom).

Table S1A. All Mascot peptide identifications of the purified, recombinant TcJHR complex based on shotgun proteomics. Confident peptide identifications reported using Scaffold (Proteome Software) to probabilistically validate Mascot MS/MS search engine results (Matrix Science). The data are available from ProteomeXchange under identifier PXD028394. Column headers: Protein, name of protein construct; Accession, Uniprot identifier; Start, first peptide residue based on sequence of Uniprot entry; Sequence, peptide sequence with indicated S(ph) and T(ph) phosphorylation sites (+80), methionine oxidation (+16), and cysteine carbamidomethylation; Stop, last peptide residue based on sequence of Uniprot entry; Modifications, identified peptide modifications; m/z, mass over charge; Charge, observed precursor charge; Mass, observed precursor mass; ΔMass [ppm], mass error in parts per million; Score, Mascot score; ΔScore, Mascot delta score.

Table S1B. Mascot phosphopeptide identifications of the purified, recombinant TcJHR complex based on shotgun proteomics. Confident phosphopeptide identifications were generated using Scaffold (Proteome Software) to probabilistically validate Mascot MS/MS search engine results (Matrix Science). The data are available from ProteomeXchange under identifier PXD028394. Column headers: Protein, name of protein construct; Accession, Uniprot identifier; Phosphosite, corresponding S/T phosphorylation site of Uniprot entry; Sequence, phosphopeptide sequence with indicated S(ph) and T(ph) phosphorylation sites (+80), methionine oxidation (+16), and cysteine carbamidomethylation; m/z, mass over charge; Charge, observed precursor charge; Mass, observed precursor mass; ΔMass [ppm], mass error in parts per million; Score, Mascot score; ΔScore, Mascot delta score.

Table S2. Quantitative analysis of proteins associating with TcJHR in the presence and absence of methoprene. MaxQuant database searches facilitated confident identification and relative quantification of proteins based on a stable isotope dimethyl labeling proteomics approach. A robust permutation test was used to analyze MaxQuant data and evaluate statistically significant differences in the relative abundance of TcJHR-associated proteins. Data are available from ProteomeXchange under identifier PXD028599. Column headers: Accession, Uniprot identifier; Organism, source organism of protein; Protein names, name of protein; Peptides, number of unique peptide sequence matches; Coverage; protein sequence coverage in %; Length, length of amino acid sequence; MW, molecular weight of protein in kDa; Score, MaxQuant Score; H/L ratio, Normalized H/L ratio based on MaxQuant; P-value, based on robust permutation test using the QPPC software algorithm; H/L Count, number of identified heavy and light labeled peptide isotope features for protein; Significant; a protein is deemed statistically significant (TRUE) or insignificant (FALSE), respectively, if its average H/L ratio is greater or less than the cut value of 2-fold change at a P-value < 0.05; Enrichment, significant enrichment of protein in the presence (+) or absence (-) of methoprene.

Table S3. MaxQuant phosphopeptide identifications of the affinity-purified TcJHR complex based on quantitative phosphoproteomics with phosphopeptide enrichement. Confident phosphopeptide identifications (FDR < 1%) with phosphosite localization and relative quantitation using the MaxQuant search engine. The quantitative phosphoproteomics data are available from ProteomeXchange under identifier PXD028599. Column headers: Leading proteins, Uniprot identifier protein match; Sequence, the identified amino acid sequence of the peptide; Modifications, post-translational modifications contained within the identified peptide sequence; Modified sequence, peptide sequence including the post-translational modifications in brackets after the modified amino acid; Phospho (ST) Probabilities, peptide sequence including the phosphosite positioning probabilities ([0..1], where 1 is best match); Charge, the charge-state of the precursor ion; m/z, the recalibrated mass-over-charge value of the precursor ion; Mass, the predicted monoisotopic mass of the identified peptide sequence; Mass error [ppm], Mass error of the precursor ion in parts per million; H/L ratio normalized, Normalized ratio between heavy and light label partners; Intensity, Summed up extracted ion current (XIC) of all isotopic clusters associated with the identified AA sequence; Intensity L, Summed up XIC of the isotopic cluster belonging to the light label partner; Intensity H, Summed up XIC of the isotopic cluster belonging to the heavy label partner.
